# Risk of fractures in half a million survivors of 20 cancers: a population-based matched cohort study using linked English electronic health records

**DOI:** 10.1016/S2666-7568(23)00285-4

**Published:** 2024-03

**Authors:** Eva Buzasi, Helena Carreira, Garth Funston, Kathryn E Mansfield, Harriet Forbes, Helen Strongman, Krishnan Bhaskaran

**Affiliations:** a Department of Non-Communicable Diseases Epidemiology, London School of Hygiene & Tropical Medicine, London, UK; b Wolfson Institute of Population Health, Barts and The London School of Medicine and Dentistry, Queen Mary University of London, London, UK

## Abstract

**Background:**

A history of multiple myeloma, prostate cancer, and breast cancer has been associated with adverse bone health, but associations across a broader range of cancers are unclear. We aimed to compare the risk of any bone fracture and major osteoporotic fractures in survivors of a wide range of cancers versus cancer-free individuals.

**Methods:**

In this population-based matched cohort study, we used electronic health records from the UK Clinical Practice Research Datalink linked to hospital data. We included adults (aged ≥18 years) eligible for linkage, and we restricted the study start to Jan 2, 1998, onwards and applied administrative censoring on Jan 31, 2020. The cancer survivor group included survivors of the 20 most common cancers. Each individual with cancer was matched (age, sex, and general practice) to up to five controls (1:5) who were cancer-free. The primary outcomes were any bone fracture and any major osteoporotic fracture (pelvic, hip, wrist, spine, or proximal humeral fractures) occurring more than 1 year after index date (ie, the diagnosis date of the matched individual with cancer). We used Cox regression models, adjusted for shared risk factors, to estimate associations between cancer survivorship and bone fractures.

**Findings:**

578 160 adults with cancer diagnosed in 1998–2020 were matched to 3 226 404 cancer-free individuals. Crude incidence rates of fractures in cancer survivors ranged between 8·39 cases (95% CI 7·45–9·46) per 1000 person-years for thyroid cancer and 21·62 cases (20·18–23·18) per 1000 person-years for multiple myeloma. Compared with cancer-free individuals, the risk of any bone fracture was increased in 15 of 20 cancers, and of major osteoporotic fractures in 17 of 20 cancers. Effect sizes varied: adjusted hazard ratios (HRs) were largest for multiple myeloma (1·94, 95% CI 1·77–2·13) and prostate cancer (1·43, 1·39–1·47); HRs in the range 1·20–1·50 were seen for stomach, liver, pancreas, lung, breast, kidney, and CNS cancers; smaller associations (HR <1·20) were observed for malignant melanoma, non-Hodgkin lymphoma, leukaemia, and oesophageal, colorectal, and cervical cancers. Increased risks of major osteoporotic fracture were noted most substantially in multiple myeloma (2·25, 1·96–2·58) and CNS (2·12, 1·56–2·87), liver (1·62, 1·01–2·61), prostate (1·60, 1·53–1·67), and lung cancers (1·60, 1·44–1·77). Effect sizes tended to reduce over time since diagnosis but remained elevated for more than 5 years in several cancers, such as multiple myeloma and stomach, lung, breast, prostate, and CNS cancers.

**Interpretation:**

Survivors of most types of cancer were at increased risk of bone fracture for several years after cancer, with variation by cancer type. These findings can help to inform mitigation and prevention strategies.

**Funding:**

Wellcome Trust.

## Introduction

About half of those diagnosed with cancer in the UK in 2010 and 2011 survived at least 10 years beyond their diagnosis, leading to an increasing population of cancer survivors.^1^ There are concerns that people with a history of cancer might be at high risk of adverse bone-related outcomes due to shared risk factors between cancer and bone disease, such as smoking and physical inactivity; direct adverse bone consequences of cancer, including inflammation-driven loss of bone mineral density (BMD) and metastases; and adverse effects of anticancer treatments such as endocrine therapies that drastically reduce sex hormones essential for bone turnover.^2^

Fractures are associated with a high burden of morbidity and mortality, reduced quality of life, and considerable health-care costs.^3^, ^4^ There is evidence of increased fracture risk in individuals with multiple myeloma, a haematological malignancy involving extensive bone destruction.^5^ For solid cancers, most research on the association between cancer and skeletal outcomes has centred on endocrine-related cancers of the breast and prostate.^6^ Even though hormonal therapies used to treat these cancers often lead to loss of BMD,^2^, ^6^ estimates of the association with fracture risk have been mixed,^7^, ^8^ ranging from no association to a quadrupling of risk in breast cancer survivors.^9^, ^10^, ^11^, ^12^ There is little evidence about bone health for other malignancies. A recent study in the USA found a risk of fracture two-times higher in cancer survivors 1–5 years after diagnosis compared with individuals with no history of cancer, with pronounced risks associated with chemotherapy, but site-specific data were only available for breast, prostate, and colorectal cancers.^13^ A 2009 study in Denmark examined fracture risk in patients with a range of cancers and demonstrated heterogeneous associations between the different cancer types.^11^ Other evidence is largely based on studies that investigated changes in BMD, bone turnover markers, or osteoporosis, rather than fractures. Much of the evidence is also outdated, with the relevance of older estimates unclear in the context of a rapidly changing landscape of cancer diagnosis and treatment.

**Research in context**

**Evidence before this study**

We searched Ovid MEDLINE(R) for epidemiological studies, reviews, and guidelines published in English from database inception (1946) to May 11, 2023, using search terms for bone fractures and cancer (search terms are provided in the appendix p 69), and searched reference lists of relevant articles. We identified articles that provided estimates comparing risks of bone fractures between adult survivors of one or more site-specific cancers and controls without a history of cancer. 19 studies were included; eight focused on risk of bone fracture in breast cancer survivors, eight included other single sites (including cervix, stomach, pelvis, prostate, multiple myeloma, and thyroid) and three included any cancer sites. Relative risk estimates from previous studies were extracted and are displayed in the appendix (pp 63–69). Neither cervical cancer nor thyroid cancer was associated with an increased risk of any fracture. Some evidence was found of increased risks of any or site-specific fracture in patients after breast cancer, while all studies investigating the risk of any fracture in prostate cancer (n=2), gastric cancer (n=1), and multiple myeloma (n=2) found an increased risk. Pelvic cancer was associated with an increased risk of hip fracture in one study.

**Added value of this study**

Our study is, to our knowledge, one of the largest to date to compare risks of fracture between adult survivors of multiple site-specific cancers and controls with no history of cancer, with a consistent methodological approach that allowed us to reveal detailed patterns of risk. We found that survivors of most site-specific cancers had increased risk of any fracture and major osteoporotic fractures, but patterns of risk varied by cancer site. The increased risk of fracture persisted beyond 5 years from diagnosis in several cancers, although the magnitude of association tended to reduce over time since cancer diagnosis.

**Implications of all the available evidence**

The available evidence to date suggests increased risks of fracture among survivors of a wide range of cancers. This increased risk has implications for quality of life, and for downstream health consequences in the growing population of cancer survivors. Impacts might be minimised through raising awareness among patients and clinicians, and through appropriate prevention and management strategies.

To address limitations in the evidence base to date, we aimed to conduct a broad analysis of the association between survivorship from the 20 most common adult cancers in the UK^14^ and adverse bone health outcomes. We used large-scale linked electronic health records databases and compared fractures in survivors of site-specific cancer with cancer-free comparators.

## Methods

### Study design and data source

We conducted a population-based matched cohort study. Data on cancer survivors and cancer-free individuals were extracted from the UK Clinical Practice Research Datalink (CPRD) GOLD (July, 2021 version) and Aurum (January, 2022 version) primary care databases, which include anonymised medical records from consenting general practices that use Vision (GOLD) and EMIS Web (Aurum) software systems. They cover about 20% of the UK population and are broadly representative in respect of age, sex, and ethnicity.^15^, ^16^ The data include information on demographics, lifestyle factors, symptoms, diagnoses and prescriptions, and referrals to secondary care.^15^, ^16^ We also obtained linked data on: (1) hospital admissions from Hospital Episode Statistics (HES) Admitted Patient Care, (2) official death registrations from Office for National Statistics, and (3) postcode-based individual-level Index of Multiple Deprivation (IMD). These linkages were only available for practices in England, which restricted the geographical coverage of our study.

The study protocol (appendix p 2) was approved by the London School of Hygiene & Tropical Medicine Research Ethics Committee (25849) and the CPRD Research Data Governance Committee (21_000405).

### Study population

The study population was drawn from adults (aged ≥18 years) in CPRD GOLD or Aurum, and eligible for linkage. We restricted the study start to the calendar period covered by all linked sources (Jan 2, 1998, onwards) and applied administrative censoring on Jan 31, 2020, due to changes in consultation behaviours during the COVID-19 pandemic.^17^

The cancer survivor group included all individuals with an incident diagnosis of one of the following cancer types as first cancer: oral cavity, oesophageal, stomach, colorectal, liver, pancreatic, lung, malignant melanoma, breast (female), cervical, uterine, ovarian, prostate, kidney, bladder, CNS, thyroid, non-Hodgkin lymphoma, multiple myeloma, and leukaemia. We identified cancers by using Read codes in CPRD GOLD; by a combination of SNOMED, Read, and EMIS Web codes in CPRD Aurum; and International Classification of Diseases version 10 (ICD-10) codes recorded in any diagnostic position in HES. To ensure that cancers were incident, we only included individuals with more than 12 months of follow-up in CPRD before first cancer code. Individuals with cancer were eligible to be selected as controls before the date of their first cancer (defined as the index date).

For each individual with cancer, we randomly selected up to five controls from the overall population with no history of cancer on the index date (ie, the diagnosis date of the matched individual with cancer), matched on index date, year of birth (±3 years), sex, and primary care practice; controls had to be under follow-up and were required to have at least 12 months of continuous registration before index date, mirroring the requirement for cancer survivors.

### Outcomes

The primary outcomes were any bone fracture and any major osteoporotic fracture (pelvic, hip, wrist, spine, or proximal humeral fractures) occurring more than 1 year after index date. Secondary outcomes were site-specific major osteoporotic fractures more than 1 year after index date. Fractures were identified using Read, SNOMED, and EMIS Web codes in primary care and ICD-10 codes in HES.

### Covariates

Demographics and covariates available other than age, sex, and general practice (matching factors) were ethnicity, calendar year at index date, socioeconomic deprivation (IMD quintile), fracture as an adult pre-dating index date, previous ever use of hormone replacement therapy, oral corticosteroids, and bisphosphonate (from primary care prescriptions), lifestyle factors (BMI [from weight and height records], smoking, alcohol consumption, or problematic alcohol drinking), autoimmune disorders (coeliac disease, inflammatory bowel disease, systemic lupus erythematosus, and rheumatoid arthritis), chronic kidney disease, liver disease, and epilepsy (appendix p 11). Study variables were evaluated at index date. Code lists are available online.

### Statistical analysis

Follow-up started 1 year after index date and ended at the earliest of: fracture outcome, any cancer diagnosis in the control group, a second primary cancer in those with history of cancer, end of registration with primary care practice, end of data collection from practice, death, or end of the study period. By censoring the competing risk of death, our analysis focused on the cause-specific hazard.^18^

We described demographic characteristics overall and by cancer site at the index date. All analyses were carried out separately by cancer site, restricting to individuals with a history of the specific cancer and their matched controls. We calculated crude fracture incidence rates in cancer and control groups. We then estimated hazard ratios (HRs) using Cox regression stratified by matched set, with time since index date as the underlying timescale. Initially, we estimated minimally adjusted HRs (implicitly controlling for matching variables). We then estimated fully adjusted HRs by additionally adjusting for problem drinking, smoking status, BMI (four-knot restricted cubic splines), quintile of IMD, chronic kidney disease, autoimmune disorder, liver disease, epilepsy, and ever use of corticosteroids, hormone replacement therapy, and bisphosphonates, subject to data sparsity checks. People with missing smoking or BMI data were omitted from models using those covariates (complete case analysis) as missingness levels were low. Ethnicity and alcohol consumption both had substantial missingness, so we excluded these from our main models and used them in sensitivity analyses only (see below). We instead controlled for alcohol-related factors in our main model using a binary problematic alcohol drinking variable. We checked for proportional hazards by testing for a non-zero slope of the scaled Schoenfeld residuals over time; we also fitted an interaction with time-updated time since index date in a secondary analysis (coded as 1–1·9, 2–4·9 years, and ≥5 years). For the primary outcomes only, we evaluated interactions between exposure (cancer diagnosis) and prespecified key covariates (one at a time), namely age group at index date (18–59 years, 60–79 years, and ≥80 years), sex (male and female), ethnicity (White, South Asian, Black, and Others, with minority ethnic grouped when the data were sparse), BMI (classified as obese *vs* non-obese for interaction analysis), and previous history of fracture as an adult (yes or no).

We created cumulative incidence curves standardised for the covariates' distribution of the cancer survivor group, to adjust for confounders.^19^ We fitted a Royston-Parmar model with the covariates of the fully adjusted Cox model, with the baseline hazard modelled using a spline with three degrees of freedom.^20^ The survival function was predicted from this model for every cancer survivor and averaged to produce the respective curve. To produce the standardised curve for the non-cancer controls, the survival functions were predicted and averaged again but with cancer survivorship status set to 0.

We tested the robustness of our findings in multiple sensitivity analyses: (1) repeating the analyses using CPRD Aurum only; (2) limiting to those without missing ethnicity data and additionally adjusting for ethnicity; (3) limiting to those with alcohol consumption data and replacing problematic alcohol drinking with alcohol consumption; (4) excluding people with a previous fracture in adulthood; (5) restricting to those with index date after 2006, when completeness of some variables was higher;^16^ (6) including follow-up in the first year after index date; (7) excluding individuals with comorbidities at baseline.

All data management and analyses were carried out in STATA version 17.

### Role of the funding source

The funder of the study had no role in study design, data collection, data analysis, data interpretation, or writing of the report.

## Results

578 160 adults with incident cancer were matched to 3 226 404 individuals with no cancer history (appendix pp 70–71). Median follow-up from the index date was 4·39 years (IQR 2·25–8·13) in the cancer survivor group and 5·72 years (3·03–9·74) in the cancer-free control group. Demographic and lifestyle-related factors were broadly similar between cancer survivors and people without a history of cancer (table; appendix p 14). However, cancer survivors were less likely than controls to be current smokers and more likely to be ex-smokers. 126 021 (21·80%) cancer survivors had a history of fracture compared with 672 188 (20·83%) controls. Cancer survivors were also more likely than controls to have chronic kidney disease and inflammatory bowel disease, and to have been prescribed oral corticosteroids. Characteristics of the matched cohorts for individual cancer sites are available in the appendix (pp 16–54).

**Table:** Characteristics of cancer survivors and matched controls from the general population (all cancer sites)

	Cancer survivors (n=578 160)	Cancer-free control population (n=3 226 404)
Time from index date*to end of follow-up, years		
Mean (SD)	5·76 (4·40)	6·88 (4·69)
Median (IQR)	4·39 (2·25–8·13)	5·72 (3·03–9·74)
Range	1·00–21·07	1·00–21·08
**Overall**		
Total person-years (million years)†	3 331 130·8	22 183 583·0
Age, years		
Mean (SD)	65·71 (13·75)	66·05 (13·65)
Median (IQR)	67 (57–76)	67 (57–76)
**Age group at cancer diagnosis**‡		
18–59 years	174 606 (30·20%)	941 980 (29·20%)
60–79 years	311 935 (53·95%)	1 756 415 (54·44%)
≥80 years	91 619 (15·85%)	528 009 (16·37%)
**Sex**‡		
Women	300 957 (52·05%)	1 659 284 (51·43%)
Men	277 203 (47·95%)	1 567 120 (48·57%)
**Patient-level Index of Multiple Deprivation (general practice postcode-based)**§		
1 (least deprived)	141 305 (24·44%)	770 052 (23·87%)
2	133 541 (23·10%)	734 716 (22·77%)
3	114 955 (19·88%)	646 738 (20·05%)
4	102 733 (17·77%)	584 732 (18·12%)
5 (most deprived)	85 153 (14·73%)	487 441 (15·11%)
**Calendar year of cancer diagnosis**		
1999–2003	113 856 (19·69%)	639 852 (19·83%)
2004–08	141 683 (24·51%)	801 735 (24·85%)
2009–13	156 158 (27·01%)	873 755 (27·08%)
2014–19	166 463 (28·79%)	911 062 (28·24%)
**Ethnicity**‡		
White	369 851 (63·97%)	2 090 130 (64·78%)
South Asian	10 421 (1·80%)	82 167 (2·55%)
Black	10 229 (1·77%)	54 355 (1·68%)
Other	4824 (0·83%)	32 112 (1·00%)
Unknown	182 835 (31·62%)	967 640 (29·99%)
**BMI**‡		
Underweight (<18·5 kg/m^2^)	11 042 (1·91%)	50 645 (1·57%)
Normal weight (18·5–24·9 kg/m^2^)	196 527 (33·99%)	1 026 894 (31·83%)
Overweight (25·0–29·9 kg/m^2^)	205 596 (35·56%)	1 099 742 (34·09%)
Obese (≥30·0 kg/m^2^)	129 777 (22·45%)	698 308 (21·64%)
Unknown	35 218 (6·09%)	350 815 (10·87%)
**Alcohol consumption**		
Non-drinker	54 722 (9·46%)	317 126 (9·83%)
Current drinker	323 431 (55·94%)	1 721 534 (53·36%)
Ex-drinker	25 448 (4·40%)	123 676 (3·83%)
Unknown	174 559 (30·19%)	1 064 068 (32·98%)
Problematic drinking	16 939 (2·93%)	89 040 (2·76%)
**Smoking status**		
Non-smoker	183 580 (31·75%)	1 075 246 (33·33%)
Current smoker	80 652 (13·95%)	574 121 (17·79%)
Ex-smoker	304 522 (52·67%)	1 497 252 (46·41%)
Unknown	9406 (1·63%)	79 785 (2·47%)
**Prescribed**		
Oral corticosteroids	121 941 (21·09%)	538 821 (16·70%)
Hormone replacement therapy	103 513 (17·90%)	502 970 (15·59%)
Bisphosphonate therapy	17 577 (3·04%)	89 348 (2·77%)
**History of**		
Fracture¶	126 021 (21·80%)	672 188 (20·83%)
Osteoporosis	31 375 (5·43%)	150 341 (4·66%)
**Comorbidities**		
Eating disorder	2417 (0·42%)	12 034 (0·37%)
Coeliac disease	2325 (0·40%)	12 453 (0·39%)
Inflammatory bowel disease	29 545 (5·11%)	104 467 (3·24%)
Systemic lupus erythematosus	1452 (0·25%)	6629 (0·21%)
Rheumatoid arthritis	12 664 (2·19%)	60 566 (1·88%)
Chronic kidney disease	140 568 (24·31%)	658 123 (20·40%)
Liver disease	13 455 (2·33%)	31 121 (0·96%)
Epilepsy	12 192 (2·11%)	55 634 (1·72%)

Data are n (%) unless otherwise stated. The characteristics of the study participants were measured before or near the index date. When information on BMI, height and weight, alcohol consumption, and smoking status was unavailable before the index date, we used information recorded at any point in the clinical record. We ran a sensitivity analysis using only pre-index data.

* For individuals in the cancer-free cohort, the index date (ie, date of study start) was the date of cancer diagnosis of the matched cancer survivors. Cancer-free people were individually matched on year of birth (±3 years), sex, and general practice to participants in the cancer survivor cohort.

† Index date to end of follow-up.

‡ Interactions investigated. Effect modification by ethnicity was studied in secondary analysis due to missing data. For interaction analysis, BMI was classified as obese versus non-obese.

§ Patient-level Index of Multiple Deprivation is an area-based proxy for socioeconomic status.

¶ In adulthood.

**Figure 1:**
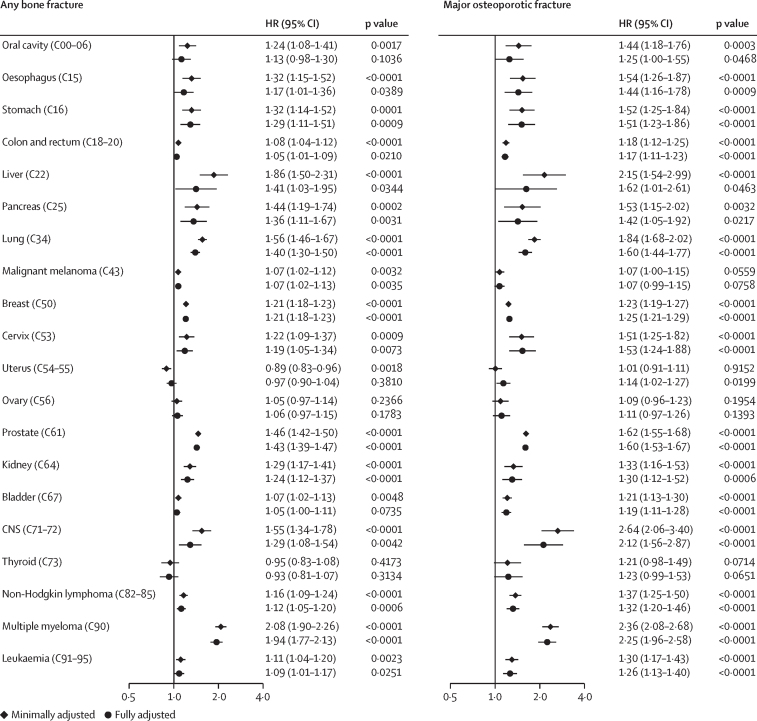
Associations between cancer survivorship and any bone fracture, and major osteoporotic fractures Black diamonds represent results from minimally adjusted models. Black circles represent results from fully adjusted models. Cox proportional hazards regression model was used to estimate the HRs. Minimally adjusted estimates were controlled for the matching factors only (age, sex, and general practice). Fully adjusted models also included problem drinking, smoking status, BMI, patient-level postcode-based Index of Multiple Deprivation, chronic kidney disease, autoimmune disorders, liver disease, epilepsy, and use of corticosteroids, hormone replacement therapy, and bisphosphonates. Codes are International Classification of Diseases-10 codes. HR=hazard ratio.

Crude incidence rates of fractures in cancer survivors ranged between 8·39 cases (95% CI 7·45–9·46) per 1000 person-years for thyroid cancer and 21·62 cases (20·18–23·18) per 1000 person-years for multiple myeloma (appendix pp 56–61). HRs comparing fracture risk in cancer survivors to controls in minimally adjusted (implicitly for the matching factors of age, sex, and general practice) and fully adjusted models were largely similar (figure 1). Compared with non-cancer controls, after fully adjusting, we found evidence of increases in bone fractures in cancer survivors for 15 of 20 cancer sites (oesophagus, stomach, colorectal, liver, pancreas, lung, malignant melanoma, breast, cervix, prostate, kidney, CNS, non-Hodgkin lymphoma, multiple myeloma, and leukaemia; p<0·05 in each case), and increased major osteoporotic fracture risk in cancer survivors for 17 of 20 cancer sites (oral cavity, oesophagus, stomach, colorectal, liver, pancreas, lung, breast, cervix, uterus, prostate, kidney, bladder, CNS, non-Hodgkin lymphoma, multiple myeloma, and leukaemia; p<0·05 in each case). Effect sizes for the association between cancer survivorship and any bone fracture ranged from HR 1·05 (95% CI 1·01–1·09) in colorectal cancer survivors to 1·94 (1·77–2·13) in multiple myeloma survivors. Precision was lower for the site-specific fracture outcomes, but we found evidence of increased risks of pelvic, hip, and spine fractures across multiple cancer survivor groups; fewer associations were seen for wrist and proximal humeral fractures. However, notably, prostate cancer survivors had increased risks of both fractures (appendix p 72).

**Figure 2:**
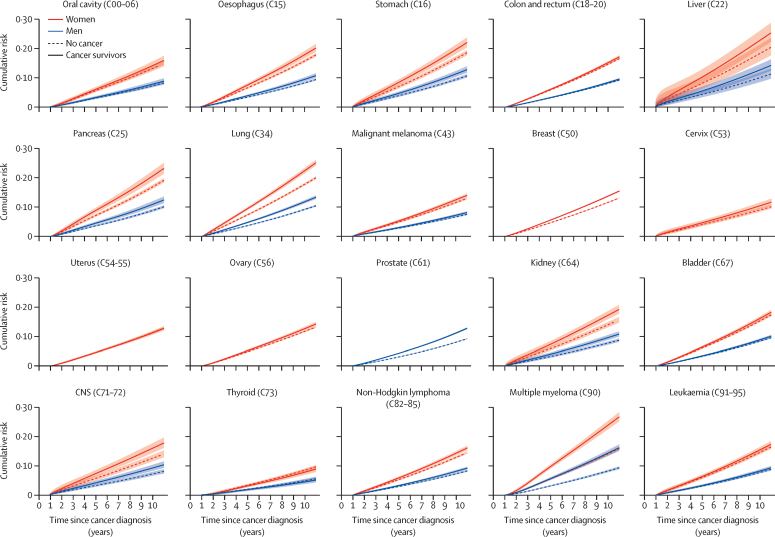
Cumulative incidence of any fracture during the study period in cancer survivors and non-cancer controls by sex, with 95% CIs Cumulative incidence predicted from a Royston-Parmar model including age, sex (when applicable), BMI (cubic spline), Index of Multiple Deprivation, smoking, problematic alcohol use, use of corticosteroids, bisphosphonates, and hormone replacement therapy, and history of chronic kidney disease, liver disease, epilepsy, and autoimmune conditions, with the baseline hazard parametrised as a three-degrees-of-freedom cubic spline; predictions standardised to the covariate distribution of the cancer survivor group. Codes are International Classification of Diseases-10 codes.

Figure 2 shows standardised cumulative incidence estimates for any fracture, stratified by sex; estimates for major osteoporotic fractures are provided in the appendix (p 73). Point estimates of 5-year and 10-year cumulative incidence are also provided in the appendix (p 62). Women had a higher risk of fractures for all cancers, with the highest 10-year incidences observed for multiple myeloma, and lung and liver cancers. The largest differences in the risk of fractures between survivors and people with no history of cancer were noted, for both men and women, in multiple myeloma and lung cancers.

**Figure 3:**
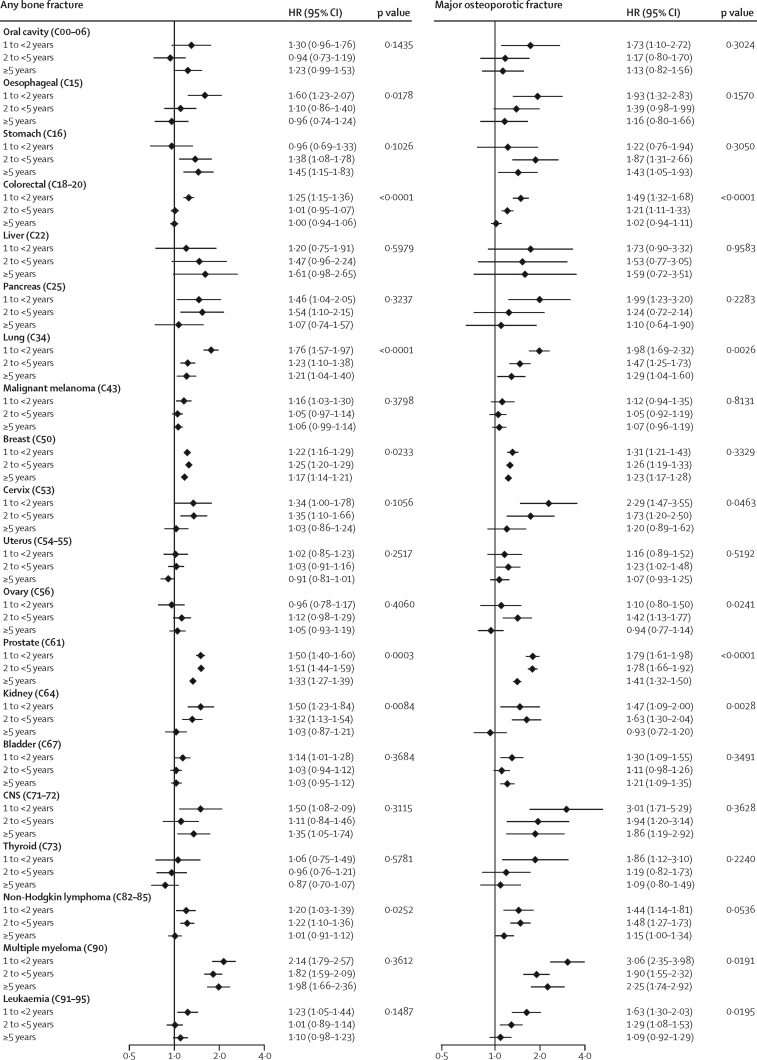
Role of time since cancer diagnosis in the associations between cancer survivorship and bone fracture and major osteoporotic fracture outcomes in individuals with history of cancer compared with cancer-free individuals Time since diagnosis split in intervals of 1 year to less than 2 years, 2 years to less than 5 years, and 5 years or more. Data are stratum-specific HRs (95% CI) and p values for interaction. Codes are International Classification of Diseases-10 codes. HR=hazard ratio..

The estimates for associations reduced with time since cancer diagnosis for some cancers, most notably non-Hodgkin lymphoma and oesophagus, colorectal, pancreas, cervix, kidney, and bladder cancers, all of which had estimated HRs for any fracture that were close to 1 by at least 5 years from index (figure 3). However, there was evidence of continuing increased risks of first fracture beyond 5 years for survivors of multiple myeloma and stomach, lung, breast, prostate, and CNS cancers.

The association between cancer survivorship and risk of bone fracture was less pronounced at older ages for lung, breast, and uterine cancers and multiple myeloma, but the opposite was seen for prostate cancer (appendix p 74). We also found strong evidence of differences by sex for multiple myeloma and liver and lung cancers; in all cases, the HRs for bone fracture were stronger for male survivors. There was little evidence for effect modification by obesity status, except for breast cancer where women with obesity had higher risk of fractures. There was little variation of fracture risk by ethnicity, except that White and South Asian survivors of prostate cancer had an increased risk of fracture, which was not seen in Black or other ethnic groups. For some cancers (oesophageal, pancreas, lung, and CNS), the risk of fracture was higher for people with no previous fracture than for those with a previous fracture, and the opposite was true for survivors of cervical cancer.

Proportional hazards were implicitly checked in our estimation of HRs stratified by time since index date (figure 3). Tests of Schoenfeld residuals led to similar conclusions, with no statistical evidence of non-proportionality for most cancer sites, and some non-proportionality for prostate cancer and breast cancer, for which statistical power is higher (data not shown). There was no indication of collinearity of adjustment factors based on observed changes in standard errors between unadjusted and adjusted models (data not shown).

Sensitivity analyses, repeating the analysis in CPRD Aurum, adjusting for ethnicity, adjusting for alcohol consumption rather than problematic alcohol drinking, restricting to patients with no previous fracture, restricting to data from 2006 onwards, restricting to patients with no comorbidities, and including the year after cancer diagnosis in the main analysis, resulted in no meaningful changes in the HRs (pp 76–77).

## Discussion

In this population-based cohort study, we found an increased risk of bone fracture among survivors of 15 of 20 cancers studied, and an increased risk of major osteoporotic fractures for 17 of 20 cancers, after accounting for shared risk factors. The size of risk increase varied by cancer type; multiple myeloma and prostate cancer had the largest increased risks of fracture overall, while survivors of multiple myeloma, and CNS, liver, prostate, and lung cancers had the most pronounced increases for major osteoporotic fracture. Observed effect sizes tended to reduce over time since diagnosis, but risks of fracture remained elevated after 5 years for several cancers. There was variation in the associations by age at cancer diagnosis and sex for some cancers: HRs for fracture associated with lung, breast, and uterus cancer, and multiple myeloma survivorship were larger in younger individuals, while for lung cancer, liver cancer, and multiple myeloma, increased risks were more pronounced in men than women.

The varying patterns of fracture risk observed across cancer sites might indicate distinct underlying mechanisms. For CNS tumours, the potential causes are likely to include direct consequences of the cancer or its treatment on bone health, on balance, and on motor deficits such as gait impairment that affect fall risk.^21^ The increased risk of bone fracture in breast and prostate cancer survivors might be related to the tendency of these cancers to spread to the spine and pelvis, in addition to lowered sex hormone levels induced by cancer treatments.^2^ The increased susceptibility to fractures observed with other genital organ cancers such as ovarian cancer might also be attributed to the influence of treatment-related sex steroid deficiencies. Additionally, surgery may interfere with mechanisms closely tied to bone physiology. For example, in patients with bladder cancer, radical cystectomy and urinary diversion result in chronic metabolic acidosis, which then causes bone loss through enhanced bone resorption and loss of urinary calcium.^22^ The limited impacts of covariate adjustment suggest that the shared risk factors considered in the study were not important drivers of the observed associations, although we cannot rule out a role for other unmeasured factors.

Our results are consistent with a Danish study^11^ with pre-2000 data that showed increased fracture risks of several cancer sites, and with a recent US study that estimated higher risks of frailty-related fractures in people with a history of any cancer, particularly at 1–5 years from diagnosis and for those with advanced stage at diagnosis.^13^ The US Women's Health Initiative Observational Study also looked at multiple cancer groups, reporting an increased total fracture risk with an HR of 1·33 (95% CI 1·18–1·49, p<0·001) for cancers of the colon, rectum, lung, and uterus, and melanoma and non-Hodgkin lymphoma combined.^23^ We found significantly elevated risks of any fracture and of major osteoporotic fracture across these cancer types. Other studies of individual cancer sites have largely focused on breast and prostate cancer, generally finding increased fracture risks in survivors of these cancers, in keeping with our results although with some variation in estimated effect sizes.^10^, ^24^, ^25^, ^26^ There are few studies on survivors of other types of cancer; a study in South Korea among women with previous cervical cancer did not find a significantly increased risk of fracture in contrast with our study,^27^ while studies of multiple myeloma survivors found increased risks of fracture consistent with our data.^28^, ^29^

This study has several strengths, including our population-based data source containing prospectively collected routine health-care records for large numbers of individuals, with vast clinical and demographic information and long follow-up; this allowed a well powered and detailed investigation across a range of cancer survivor groups. We conducted several sensitivity analyses, demonstrating that our results were robust. Our primary fracture outcome should be well ascertained in routine clinical records as most fractures are likely to come to medical attention. Moreover, diagnoses data from CPRD have good validity in general^30^ as well as for cancer,^31^ and the data were enhanced further through linkages to key national datasets. CPRD is broadly representative of the UK population in terms of age, sex, and ethnicity,^15^, ^16^ increasing confidence in the generalisability of our findings to the wider UK population and comparable settings. However, our study also has limitations. There is a possibility of outcome-detection bias if cancer survivors had more regular contact with health-care services, closer follow-up care, or more frequent imaging, which would increase the likelihood of fracture diagnosis, especially the discovery of asymptomatic vertebral fractures. It was not possible to ascertain the site of fracture in all cases for the secondary outcome of site-specific fractures. We relied on fracture location to define major osteoporotic fractures because clinical codes did not consistently specify fracture context or aetiology. Therefore, we might have misclassified a small proportion of fractures due to trauma or metastatic disease as osteoporotic. Most fractures in older people are related to osteoporosis so the impact of this is likely to be minimal.^3^ Consistent with all observational epidemiology, unmeasured confounding is possible, particularly due to our inability to robustly capture physical activity and diet using electronic health records. However, adjustment for BMI might have partly controlled for these factors. Furthermore, we only had basic smoking data, with no information on quantity of smoking; we also relied on smoking being accurately reported and recorded by the doctor. Our deprivation measure was postcode-based and might not accurately reflect individual-level conditions. We did not have data on cancer stage, treatment, or progression, so we could not explore the role of these factors. We also lacked information regarding menopausal status in women, although age matching should have taken some account of this. We did not account for dose or length of use of hormone replacement therapy, oral corticosteroids, and bisphosphonates because all patients were required to have 1 year of data before index date, which is insufficient to accurately study these factors. There were missing data on smoking (2·3%) and BMI (10·2%), which were handled by using complete-case analysis. This would be unbiased providing that missingness is conditionally independent of the outcome.^32^ However, missingness was quite low, which reassures against any meaningful bias. Missingness was higher for ethnicity (30·2%) and alcohol consumption (32·6%) but adjusting for these shared risk factors in sensitivity analyses made little difference to effect estimates. Our analysis across 20 cancer types and 2 primary outcomes means that a small number of significant associations might have been expected by chance. Associations with weak statistical evidence should therefore be interpreted with caution.

We have found that most of the common cancer types are associated with some degree of increased risk of bone fracture. However, the limited guidance currently available in this area primarily focuses on only a few tumour types (breast, prostate, lung, and multiple myeloma), for which there are known risks of bone involvement or treatment-related loss of bone health.^33^ Notably, these guidelines do not address the increased risks of bone fracture we have observed across a much wider range of cancers in this study. Thus, our findings should increase awareness of increased fracture risk in survivors of multiple types of cancer and emphasise the need for prevention and effective treatment of bone complications in this large and growing population. Our detailed quantification of risks in different cancer survivor groups could be used to inform the design of targeted mitigation strategies, which might include routine assessment for fracture risk or closer monitoring of bone health and initiation of preventive treatments in high-risk groups other than those with breast and prostate cancer.^33^ In all groups of patients with cancer, multidisciplinary approaches are needed to support interventions that consider the interplay between cancer type, treatment regimen, bone health, and overall wellbeing. This holistic approach demands collaboration among oncologists, primary care physicians, endocrinologists, nutritionists, physiatrists, physiotherapists, and other relevant specialists.^34^, ^35^ From a research perspective, further work needs to be done to investigate the drivers of the increased fracture risk, particularly in relation to cancer treatment, and potential mediators of increased risks including changes in BMD, physical activity, and fall risk.

In conclusion, we found that survivors of most site-specific cancers had higher risks of any bone fracture and major osteoporotic fractures than people without history of a cancer, with a varying magnitude of risk by cancer type. These findings can help to inform mitigation and prevention strategies.

## Data sharing

This study is based in part on data from the Clinical Practice Research Datalink (CPRD) obtained under licence from the UK Medicines and Healthcare products Regulatory Agency. The terms of our licence to access the data preclude us from sharing individual patient data with third parties. The raw data may be requested directly from the CPRD following their usual procedures.
