# Supplementary material for: Risk of fractures in half a million survivors of 20 cancers: a population-based matched cohort study using linked English electronic health records
Source: Lancet Healthy Longev. 2024 Mar;5(3):e194–203. doi: 10.1016/S2666-7568(23)00285-4 (PMC10904352; doi:10.1016/S2666-7568(23)00285-4)

THE LANCET
Healthy Longevity

Supplementary appendix

This appendix formed part of the original submission and has been peer reviewed. We post it as supplied by the authors.

Supplement to: Buzasi E, Carreira H, Funston G, et al. Risk of fractures in half a million survivors of 20 cancers: a population-based matched cohort study using linked English electronic health records. *Lancet Healthy Longev* 2024; published online Feb 6. https://doi.org/10.1016/S2666-7568(23)00285-4.

SUPPLEMENTARY MATERIAL

Supplement to: Buzasi et al. Risk of fractures in half a million survivors of 20 cancers: a population-based matched cohort study using linked UK electronic health records databases.

	Page
**Pre-specified study protocol**	2
**Table S1: Definition and derivation of the study variables**	11
**Table S2: Characteristics of cancer survivors and matched controls from the general population in CPRD Aurum and GOLD databases separately (all cancer sites).***	14
**Tables S3-S22: Characteristics of cancer survivors and matched controls from the general population (site specific cancer/control cohorts)**	
Table S3: Oral Cavity (C00-06)	16
Table S4: Oesophagus (C15)	18
Table S5: Stomach (C16)	20
Table S6: Colorectal (C18-20)	22
Table S7: Liver (C22)	24
Table S8: Pancreas (C25)	26
Table S9: Lung (C34)	28
Table S10: Malignant melanoma (C43)	30
Table S11: Breast (C50)	32
Table S12: Cervix (C53)	34
Table S13: Uterus (C54-55)	36
Table S14: Ovary (C56)	38
Table S15: Prostate (C61)	40
Table S16: Kidney (C64)	42
Table S17: Bladder (C67)	44
Table S18: Central nervous system (CNS, C71-72)	46
Table S19: Thyroid (C73)	48
Table S20: Non-Hodgkin Lymphoma (NHL, C82-85)	50
Table S21: Multiple myeloma (C90)	52
Table S22: Leukaemia (C95)	54
**Tables 23-28: Bone fracture incidence in survivors of the 20 most common types of cancers and cancer-free controls.**	
Table S23: Any bone fracture and major osteoporotic fractures	56
Table S24: Pelvic fracture	57
Table S25: Hip fracture	58
Table S26: Wrist fracture	59
Table S27: Spine fracture	60
Table S28: Proximal humerus fracture	61
**Table S29: Estimated cumulative risk of any bone fracture and major osteoporotic fracture in cancer survivors at 5 and 10 years after cancer diagnosis.**	62
**Table S30: Results from systematic review.**	63
**Figure S1A: Flowchart of the selection of the cohorts used in analysis: CPRD GOLD**	70
**Figure S1B: Flowchart of the selection of the cohorts used in analysis: CPRD Aurum**	71
**Figure S2: Associations between cancer survivorship and fractures of the pelvis, hip, spine, wrist, and proximal humerus.**	72
**Figure S3: Cumulative incidence (%) of major fractures in cancer survivors and non-cancer controls by sex, with 95% confidence intervals.**	73
**Figure S4: Forest plots of the association between cancer survivorship and any fractures, stratified by potential effect modifiers.**	74
**Figure S5: Forest plots of the association between cancer survivorship and major osteoporotic fractures, stratified by potential effect modifiers.**	75
**Figure S6: Results of sensitivity analysis on the association between cancer survivorship and any fracture.**	76
**Figure S7: Results of sensitivity analysis on the association between cancer survivorship and major osteoporotic fractures.**	77

Pre-specified study protocol (as approved by CPRD Research Data Governance Committee on 23rd April 2021)

5

Protocol information

Lay Summary

Over the past decades, earlier detection of cancer through screening programmes and better cancer treatments have improved survival for many people with cancer. However, there are concerns that some cancer treatments may have long-term effects on bone health, leading to more broken bones and consequent diminished quality of life, increased risk of death and costs to healthcare services.

We will use information that is routinely collected in general practices and hospitals. We will identify people who have had cancer and a group of people of a similar age and sex who have never had cancer. We will then compare how often people with cancer experience broken bones compared to people without cancer.

We want to investigate whether some, or all, cancer survivors are at risk of developing bone problems. Understanding cancer survivors' risk of broken bones will help health services target interventions to prevent fractures, and prioritise areas for more research on how specific cancers can lead to poor bone health.

Technical Summary

Over the past decades, advances in cancer detection and treatment have resulted in improved survival, whilst raising concerns about long-term adverse consequences. It has been suggested that people receiving cancer therapy may experience higher rates of bone loss, osteoporosis and bone fracture. However, there is currently little population-based evidence on this. The relationship between cancer survival and bone health is important to understand considering the high burden of morbidity and mortality associated with fracture and consequent reduced quality of life, in addition to the substantial financial costs to the health service.

Our matched cohort study will use data from CPRD GOLD and Aurum primary care databases and linked Hospital Episodes Statistics (HES) admitted patient care data to identify cancer survivors and age/sex/GP-matched controls. Linked ONS mortality data will be used to censor deaths. We will describe the incidence of fracture and osteoporosis in cancer survivors (ascertained using primary care and linked HES data). We will use Cox regression analysis to compare the risk of bone fracture in individuals with a history of cancer (stratified by cancer site) to matched controls with no history of cancer. Linked patient-level deprivation data will allow adjustment for socioeconomic deprivation. We will also assess whether the association between having a history of cancer and developing adverse bone health outcomes changes with key individual-level factors.

The study will help to identify whether some, or all, cancer survivors are at higher risk of developing bone problems, will help health services target interventions to prevent fractures, and will help identify priority areas for more detailed research on how specific cancers can lead to poor bone health.

Outcomes to be measured

The primary outcome is bone fracture. Secondary/exploratory outcomes are major osteoporotic fractures, and diagnosed osteoporosis.

Objectives, specific aims & rationale

There is a lack of evidence on the risk of osteoporosis and fractures in people with a history of cancer. The overall aim of this study is to understand and quantify fracture risk in adult survivors of site-specific cancers and compare that risk to the risk of similar people who never had cancer.

Specific objectives of the study

Overall objective:
• to compare risk of bone fracture (primary outcome), major osteoporotic fractures (secondary outcome) and osteoporosis (secondary exploratory outcome) in individuals with a history of cancer (stratified by site) versus people with no history of cancer

Specific aims:
• to describe and compare characteristics of people in the study population with and without a history of cancer
• to describe the age/sex-stratified incidence of bone fracture, major osteoporotic fracture and osteoporosis in people with a history of cancer
• to compare risk of fracture and osteoporosis in those with and without a history cancer, controlling for key confounders in the association
• to investigate key potential effect modifiers (age, sex, ethnicity, menopausal status, BMI and prior history of fracture) for the association between cancer history and outcomes.

Study background

Over the past decades, early-stage cancer diagnosis (through screening programmes) and considerable advances in cancer treatments have resulted in improved survival for many individuals with cancer. Despite the favourable effects on their cancer, the hormonal and chemotherapies these individuals receive, raise concerns about the long-term adverse consequences. Anticancer treatments have been shown to reduce bone mineral density at rates of up to ten-fold higher than normal (Guise et al, 2006). Bone loss greatly increases the risk of osteoporosis ultimately leading to higher fracture risk in a population who are already likely to have ongoing bone changes due to the aging process (Khan et al, 2011). Pathological changes in bone and fractures can have serious adverse effects on quality of life, lead to increased risk of mortality and overall healthcare costs (Guise et al, 2006).

A 2011 study in the (then) General Practice Research Database found an elevated incidence of osteoporosis in breast cancer (HR 1.26, 95% CI 1.13–1.40), prostate cancer (HR 2.49, 95% CI 1.93–3.22) and colorectal cancer survivors (HR 1.41, 95% CI 1.15–1.73) (Khan et al, 2011). However, cancer treatments have changed rapidly, and the current relevance of these estimates is unclear. In addition, the long-term effects on bone health in the broader population of survivors from cancers in other sites remains unclear.

More recent studies from other settings have had mixed findings, but suggest increased risks of bone health problems in various cancer survivor groups: a Korean study found lower bone mineral density in young cancer survivors compared with healthy controls (Kim et al, 2020); while a study in use Veterans' Affairs data showed a raised incidence of osteoporosis, but not fracture, in those with thyroid cancer (Papaleontiou et al, 2019). The drug tamoxifen, used to prevent breast cancer recurrence, was hypothesised to have adverse effects on bone health, but data from Korea found no evidence of association between tamoxifen use and osteoporosis/osteoporotic fracture in breast cancer survivors, although confidence intervals were wide (Lee et al, 2020).

With a limited consensus in the literature to date on the link between cancer survivors and fractures/osteoporosis, we aim, in a population-based cohort study, to conduct a broad analysis of the association between cancer survivorship, stratified by cancer site, and adverse bone outcomes, and to identify potential areas of concern for future research on bone health in cancer survivors.

Study type

This is a hypothesis-testing study to quantify the association between cancer history and bone fracture and osteoporosis in adults (18 years old). The null hypothesis is that there is no association between having a history cancer, and adverse bone outcomes.

Study design

This is a retrospective comparative matched cohort study of adults (18 years old) treated in routine UK care settings, including those under hospital care and in registered primary care practices contributing to CPRD.
This study will use CPRD linked to Hospital Episode Statistics (HES) data. The study will be restricted to the coverage period of HES, and we will additionally censor follow-up at end of January 2020 since consultations and recording of outcomes during the coronavirus pandemic may have been atypical. Individuals with incident cancer at any of the 20 most common cancer sites (see study population) during the study period will be individually matched 1:5 with individuals without cancer on age, sex and GP practice. The matched cohort without cancer will be selected from the total population of adults registered in CPRD who meet the inclusion criteria (see Section L) on the basis of age, sex and GP practice.
Individuals who meet the inclusion criteria (see relevant section) will be assigned an index date.
- In those with prior cancer, index date will be assigned as the date of cancer diagnosis.
- In the matched cohort without cancer, the index date will be assigned to each individual as the date of cancer diagnoses of their matched cancer patient.
- The primary analysis will start follow-up at 1 year post-index date, since our focus is on medium to long-term risks, and acute cancer treatments in the first year, as well as bone involvement in the cancer itself might increase the short-term risk of fractures.

Individuals will be followed-up until the earliest of the following dates:
• Death (identified using ONS mortality data)
• Transfer out of practice date
• Practice last collection date
• Date of end of data collection in HES
• First fracture event during follow-up

Feasibility counts

Feasibility counts presented below are based on the July 2020 versions of the CPRD GOLD primary care databases. The projected total assumes that Aurum contributes 1.75 times the data to GOLD (based on the number of acceptable patients in the two datasets - ~35 million versus 20 million), and that 50% of GOLD practices and 100% of Aurum practices are linked. The counts show that we should have over 800,000 cancer survivors in total, ranging from 5528 for thyroid cancer up to 167,051 for breast cancer.

Counts for site specific cancers in CPRD GOLD/Projected total in GOLD + AURUM with linkages
Bladder 17,701/39827
Breast 74,245/167051
Cervix 3,895/8763
Central nervous system 6,754/15196
Colorectal 45,878/103225
Gastric 7,149/16085
Kidney 4,807/10815
Leukaemia 12,672/28512
Liver 5,311/11949
Lung 46,773/105239
Malignant skin melanoma 19,956/44901
Multiple myeloma 6,667/15000
Non-Hodgkin lymphoma 15,540/34965
Oesophagus 12,599/28347
Oral cavity 3,599/8097
Ovary 8,657/19478
Pancreas 8,838/19885
Prostate 55,647/125205
Thyroid 2,457/5528
Uterus 7,732/17397
TOTAL 366877/825473

A comparison group of individuals without cancer (5 controls per individual with cancer) will be randomly selected from the same data source (i.e., GOLD or Aurum), same primary care practice and of the same sex and within a 3-year age range.

Sample size considerations

Assuming a 5:1 matching ratio, an overall fracture incidence rate of around 10 per 1000 person years (Bergh et al, PLoS One 2020), and an average follow-up of 5 years: - for the least common cancer group (thyroid) we expect to include 5528*6 = 33168 individuals (based on the feasibility count above, and 100% successful matching), and 33168*5 = 165840 person years, with 165840*10/1000 = 1658 fractures observed. This gives 80% power to detect a hazard ratio of 1.2 (at 5% alpha). - For the largest cancer group (breast) we expect to include 74245*6 = 445,470 individuals after matching, and 2.23 million person years, leading to 22273 fracture events. This gives 80% power to detect a hazard ratio of 1.05 (at 5% alpha). This shows that we have adequate power across the range of cancers to find clinically relevant effects for our primary outcome.

Planned use of linked data and benefit to patients in England and Wales

Linkage to HES Admitted Patient Care will provide more complete information on the study exposure (cancer) and bone fracture. They will also provide additional information on the demographic characteristics of patients. ONS mortality data will allow for appropriate censoring at death. The use of the linked HES/ONS data will limit the study population to individuals registered with consenting general practices in England, and to the time period covered by all linked sources. Individuals' follow-up will be censored at the end of data availability, therefore the use of linked HES/ONS data will also restrict the amount of follow-up time available (we will additionally censor at end January 2020 to avoid the coronavirus pandemic period). Patient-level Index of Multiple Deprivation will be used to capture socioeconomic deprivation.

The outputs of this study will benefit patients in England and Wales by helping health services to target appropriately interventions to prevent fractures, and by helping to identify priority areas for more detailed research on how specific cancers can lead to poor bone health, which will in turn aid prevention and health service planning.

Definition of the study population

The study population will be a matched cohort of individuals with and without a history of one of the 20 most common cancers (recorded in primary care or hospital admission records) identified from adults registered with CPRD GOLD or Aurum practices eligible for HES linkage. Only those individuals who contribute data to CPRD during the study period and the data that meets CPRD practice- and patient-level (in GOLD) quality control standards (assessed using up-to-standard quality marker in GOLD) will be included.

Inclusion criteria for the exposed (cancer survivors) group:

• Adults (18 years old).
• Individuals with an incident diagnosis of one of the following cancer types as a first cancer, at least 12 months after the start of CPRD follow-up: bladder, breast, cervix, central nervous system, colorectal, gastric, kidney, leukaemia, liver, lung, malignant melanoma, multiple myeloma, non-Hodgkin lymphoma, oesophagus, oral cavity, ovary, pancreas, prostate, thyroid, uterus cancer - comprehensive site-specific cancer code lists have been developed for our previous studies in the LSHTM Beyond Cancer group (e.g. Strongman et al, Lancet 2020) (ISAC protocol 16_274) and will be updated. We have also mapped these for use in Aurum in ongoing projects (e.g. ISAC 15_185R).

Exclusion criteria:
• Individuals previously diagnosed with other types of cancer will be excluded from our site-specific cancer cohorts.
• Individuals have <12 months of (up-to-standard in GOLD) follow-up before index date.

For the exploratory outcome of osteoporosis we will also exclude those with a prior diagnosis of osteoporosis/osteopenia or who have received bisphosphonates.

Selection of comparison groups/controls

The matched cohort will be selected from the total population of adults registered in CPRD eligible for HES linkage. The comparison group (without any history of cancer at index date) will be individually matched 5:1 with individuals from the exposed group on year of birth (+/- 3 years), sex and GP practice. The same inclusion and exclusion criteria will apply to controls as to the exposed group. Cases will be eligible to serve as controls until the date of their cancer diagnosis.

Exposures, outcomes and covariates

Exposures:

• History of (site-specific) cancer identified by Read codes in CPRD GOLD, SNOMED, Read and EMIS Web software-specific codes in CPRD Aurum, and ICD-10 codes in any of the diagnosis fields in HES (updated from those developed for our previous work (8), published online at https://datacompass.lshtm.ac.uk/id/eprint/1546/).

Primary outcome:

• Any bone fracture identified by relevant Read/SNOMED/EMIS codes in CPRD and ICD-10 codes in any of the diagnosis fields in HES (based on code lists from a previous project within our group: https://datacompass.lshtm.ac.uk/id/eprint/1156/).

Secondary outcome:

• Major osteoporotic fractures (of the hip, pelvis, spine and wrist) individually and combined
• (Exploratory only, due to high risk of differential ascertainment) Diagnosis of osteoporosis identified by relevant Read codes in CPRD and ICD-10 in any of the diagnosis fields in HES (based on code lists published on clinicalcodes.org: https://clinicalcodes.rss.mhs.man.ac.uk/medcodes/article/9/codelist/osteoporosis/ which will be updated using systematic keywords coding dictionary searches).

Covariates will be defined (at index date):

• (Age, sex and general practice as matching factors)
• Ethnicity
• Calendar year of cancer diagnosis/index date
• Socioeconomic deprivation (measured using twentiles of IMD)
• Smoking (non-smoker, ex-smoker, current smoker)
• Drinking status (a) categorised as non-drinker, ex-drinker, current drinker and (b) categorised as presence or absence of a code for problem drinking)
• BMI (categorised as underweight, normal, overweight, obese for descriptive purposes and modelled as a continuous spline; calculated from height and weight data)
• Oral steroid use (never, >1 year before index date, <=1 year before index date)
• Hormone replacement therapy use
• Prior (pre-index date) history of fracture as an adult
• Conditions associated with raised risk of osteoporosis: coeliac disease, inflammatory bowel disease, systemic lupus erythematosus, rheumatoid arthritis, amenorrhoea, eating disorders, chronic kidney disease, liver disease, epilepsy (NB. covariate selection will be guided by directed acyclic graph [DAG] development for each site-specific cancer analysis).

Data/statistical analysis

We will initially present demographic and comorbidity baseline characteristics (see list of covariates above) for individuals with and without a history of cancer, overall and by cancer site.

Assessing the risk of fracture in people with a history of cancer compared to matched controls

The primary and secondary outcomes will be assessed separately. The number of events and person-years for each of the outcomes will be reported. For each cancer site, crude outcome incidence rates with 95% confidence intervals will be calculated in the cohorts with and without a history of the cancer in question, both stratified by sex, and for male/female combined. We will assess if risk of each outcome is greater in people with a history of cancer compared to those without, using Cox proportional hazards regression stratified by matched set with time since index date as the underlying timescale, to estimate minimally-adjusted hazard ratios (HRs); these models will implicitly adjust for the matching variables and underlying timescale. Then, we will calculate adjusted hazard ratios, adjusting for covariates (from the list in Section N) informed by DAGs developed for site-specific cancer cohorts. We will assess collinearity by comparing standard errors in partially and fully adjusted models. We will also describe the degree of confounding arising from each variable by examining models fitted for one covariate at a time. We will explore the role of time since cancer diagnosis by fitting a time-updated "time since index date" term in a secondary analysis (coded as <1 year, 1-4.9 years, 5+ years). In a further secondary analysis we will describe the number of fractures during follow-up in the two groups (initially assuming fracture records within 3 months to be the same event – this time window will be explored descriptively and amended if needed), and use Poisson regression allowing for multiple events (with adjustment for person-level clustering) to calculate incidence rate ratios.

Bone fracture/osteoporosis and individual-level factors

To estimate how individual-level factors affect the association between having a history of cancer and developing adverse bone health outcomes, we will evaluate interactions between the exposure (cancer diagnosis) and key covariates (one at a time). Specifically, we will look at the following pre-specified interactions with exposure: age, sex, ethnicity, BMI (classified as obese vs non-obese for interaction analysis) and prior history of fracture as an adult.

Additional/sensitivity analyses

We will conduct the following additional/sensitivity analyses:

- Excluding people with a prior fracture as an adult
- Including the first year since index date (i.e., the period likely to include cancer treatment)
- Varying our missing data approach (see Missing data section)
- Restricting to data from 2006 onwards when completeness of some variables (e.g ethnicity) was higher

Plan for addressing confounding

Cancer survivors will be matched to randomly selected individuals with no history of cancer under follow-up on the date of the cancer diagnosis. Individuals will be matched on sex, year of birth (± 3 years), and general practice in order to ensure that the groups are comparable with respect to these variables.

A list of covariates that could potentially have a confounding effect on the outcome has been listed above and a DAG based on these variables will be developed to inform final model covariate selection for analysis of each site-specific cancer cohort. Bisphosphonate therapy, ascertained from primary care prescription data, will also be included as a potential confounder.

Plans for addressing missing data

The number of missing data will be reported for each variable. Missing data are anticipated in the smoking, drinking, BMI and ethnicity variables. Where the proportion of missingness is sufficiently low (<25%) we will conduct complete case analysis. For variables with higher levels of missingness, we will omit them from the primary model, and fit in a sensitivity analysis only. In additional sensitivity analyses we will take the alternative approach of classifying missing smoking as non-smoker and missing BMI as non-obese, on the basis that non-recording of these variables is more likely if there are no associated health concerns, and we will also use presence or absence of problem drinking to capture confounding by alcohol use; we will omit ethnicity from this analysis, which will then include all patients.

Patient or user group involvement

This study will not have any patient or user group involvement at the design/analysis stages due to funding restrictions. However, we will engage with relevant groups and charities (e.g., CRUK, Blood Cancer UK, Macmillian) to offer lay-friendly summaries of our findings at the dissemination stage.

Plans for disseminating & communicating

It is intended that results of this study will be published in peer-reviewed journals and presented at conferences.

Conflict of interest statement

There are no conflicts of interest to declare.

Limitations of study design

Osteoporosis is only included as an exploratory secondary outcome, because the diagnosis of osteoporosis is likely to be susceptible to ascertainment bias, with only those deemed more at-risk receiving appropriate testing, and this may differ by cancer history. On the other hand, our primary fractures outcome should be well ascertained as most fractures are likely to result in a consultation and record. It may not be possible to ascertain site of fracture in all cases, for our secondary outcome of specific fractures.

We are not linking to detailed anti-cancer treatment data in this study, so will not be able to explore the role of specific treatments in driving any excess risk.

There is a possibility that we will not have reliable information on important variables, for example, data on smoking, alcohol consumption which usually relies on patients self-reporting accurately to their GP. Furthermore, there may be no data available on menopausal status, although age matching should to some extent take account of menopausal status.

As this is an observational study, residual or unobserved confounding may remain, even after adjusting for available covariates in the datasets. Using a matched study design will help reduce the degree of confounding.

References

Bergh C, Wennergren D, Möller M, Brisby H (2020) Fracture incidence in adults in relation to age and gender: A study of 27,169 fractures in the Swedish Fracture Register in a well-defined catchment area. PLOS ONE 15(12): e0244291. https://doi.org/10.1371/journal.pone.0244291

Guise TA. Bone loss and fracture risk associated with cancer therapy. Oncologist. 2006;11(10):1121-31.

Khan NF, Mant D, Carpenter L, Forman D, Rose PW. Long-term health outcomes in a British cohort of breast, colorectal and prostate cancer survivors: a database study. Br J Cancer. 2011;105 Suppl 1(Suppl 1):S29-37.

Kim H, Yoo S, Park SG. Young cancer survivors have lower bone mineral density compared with healthy controls: a nationwide population-based study in Korea. Sci Rep. 2020 Jan 20;10(1):654. doi: 10.1038/s41598-020-57503-y. PMID: 31959794; PMCID: PMC6971234.

Lee J, Alqudaihi HM, Kang MS, Kim J, Lee JW, Ko BS, Son BH, Ahn SH, Lee JE, Han SW, Kim Z, Hur SM, Lee JS, Chung IY. Effect of Tamoxifen on the Risk of Osteoporosis and Osteoporotic Fracture in Younger Breast Cancer Survivors: A Nationwide Study. Front Oncol. 2020 Mar 20;10:366. doi: 10.3389/fonc.2020.00366. PMID: 32266146; PMCID: PMC7098996.

Papaleontiou M, Banerjee M, Reyes-Gastelum D, Hawley ST, Haymart MR. Risk of Osteoporosis and Fractures in Patients with Thyroid Cancer: A Case-Control Study in U.S. Veterans. Oncologist. 2019 Sep;24(9):1166-1173. doi: 10.1634/theoncologist.2019-0234. Epub 2019 Jun 4. PMID: 31164453; PMCID: PMC6738319.

Strongman H, Gadd S, Matthews A, Mansfield KE, Stanway S, Lyon AR, et al. Medium and long-term risks of specific cardiovascular diseases in survivors of 20 adult cancers: a population-based cohort study using multiple linked UK electronic health records databases. The Lancet. 2019;394(10203):1041-54.

Appendices

Grant ID

Table S1: Definition and derivation of the study variables

Type of variable	Covariate	Definition and use	Derivation
Exposure	Cancer	20 common cancers in the UK:<br>oral cavity, oesophageal, stomach, colorectal, liver, pancreatic, lung, malignant melanoma, breast (female), cervical, uterine, ovarian, prostate, kidney, bladder, central nervous system, thyroid, non-Hodgkin lymphoma, multiple myeloma and leukaemia.	First ever record of cancer, except for non-melanoma skin cancer, in CPRD primary care data linked to hospital admissions data (Hospital Episodes Statistics - Admitted Patient Care database).<br><br>Patients had to have at least 12 months of follow up before their cancer record to be eligible, to ensure the cancer was incident.<br><br>Informed by: The identification of incident cancers in UK primary care databases: a systematic review - PubMed (nih.gov)
Socio-demographic	Age at index date	matching variable (+/- 3 years) and categorical potential effect modifier (18-59, 60-79, ≥80)	Year of index date minus year of birth. (Exact date of birth not collected by CPRD to maintain the de-identified nature of the data)
Socio-demographic	Sex	Sex (male, female); matching variable and potential effect modifier	N/A. As provided by CPRD
Socio-demographic	Patient area-based deprivation	Categorical covariate (quintiles) and potential effect modifier used as a proxy for socio-economic status.	Index of multiple deprivation (IMD) quintile identified by CPRD through third party linkage to patient postcode
Socio-demographic	Ethnicity	Categorical covariate used in sensitivity analysis and potential effect modifier (White, South Asian, Black, Others; or White and non-white when data were sparse)	Algorithm to ascertain individual's ethnicities in CPRD primary care data, informed by: Completeness and usability of ethnicity data in UK-based primary care and hospital databases - PubMed (nih.gov)<br><br>In further sensitivity analysis, this variable was supplemented with the data available in the Hospital Episodes Statistics – Admitted Patient Care database.
Lifestyle factor	Body mass index	Potential confounder, continuous variable modelled with 3 knotted restricted cubic spline. Categorical potential effect modifier (non-obese vs. obese) based on WHO categories.	Calculated from nearest structured record of weight and height entered at any time before baseline or up to 30 days after. If this was missing, records post 30 days after baseline were used to improve completeness. Implausible measurements excluded.<br><br>Informed by: Representativeness and optimal use of body mass index (BMI) in the UK Clinical Practice Research Datalink (CPRD) - PubMed (nih.gov)
Lifestyle factor	Smoking	Categorical shared risk factor and potential effect modifier (non-smoker, smoker, ex-smoker).<br>People with missing data excluded from all models.	Main analysis: Nearest coded record of smoking status to baseline entered at any time before this date or up to 30 days after. If this was missing, smoking records post 30 days after baseline were used to improve completeness. Non-smokers re-categorised as ex-smoker if there was a previous record of smoking.

Type of variable	Covariate	Definition and use	Derivation
Lifestyle factor	Current or previous heavy drinker	Binary shared risk factor.	Read/SNOMED/EMIS-coded record of problem drinking or heavy drinking at any time prior to baseline.
Lifestyle factor	Alcohol status	Used in sensitivity analysis only, as a categorical shared risk factor [non-drinker, ex-drinker, current drinker] to replace 'current or previous heavy drinker' in models. People with missing data excluded from alcohol sensitivity models only.	Nearest coded record of alcohol status to baseline entered at any time before this date or up to 30 days after. If this was missing, smoking records post 30 days after baseline were used to improve completeness. Non-drinkers re-categorised as ex-drinkers if there was a previous record of drinking.
Primary outcome	Fracture	Binary categorical variable; primary study outcome	ICD-10 code for a fracture recorded in the Hospital Episodes Statistics - Admitted Patient Care database or Read/SNOMED/EMIS-coded record of fracture in the CPRD primary care databases.<br><br>Lowe KE, Mansfield KE, Delmestri A, Smeeth L, Roberts A, Abuabara K, et al. Atopic eczema and fracture risk in adults : A population-based cohort study. Journal of Allergy and Clinical Immunology. 2019;
Primary outcome	Major osteoporotic fracture	Binary categorical variable; primary study outcome	Same definition as the fracture, but including only fractures of the pelvis, hip, wrist, spine and proximal humerus.
Secondary outcome	Pelvic fracture	Binary categorical variable; secondary outcome	Same definition as the fracture but including only fractures of the pelvis.
Secondary outcome	Hip fracture	Binary categorical variable; secondary outcome	Same definition as the fracture but including only fractures of the hip.
Secondary outcome	Wrist fracture	Binary categorical variable; secondary outcome	Same definition as the fracture but including only fractures of the wrist.
Secondary outcome	Spinal fracture	Binary categorical variable; secondary outcome	Same definition as the fracture but including only fractures of the spine.
Secondary outcome	Proximal humeral fracture	Binary categorical variable; secondary outcome	Same definition as the fracture but including only fractures of the proximal humerus.
Covariate	History of fractures	Potential effect modifier	Same definition as the primary outcome, but with a record that pre-dated the study baseline. Fractures in childhood were not considered.

Type of variable	Covariate	Definition and use	Derivation
Covariate	Hormone replacement therapy	Categorical shared risk factor	At least one prescription of hormone replacement therapy prior to baseline (past use). Derived from primary care prescription data.
Covariate	Oral corticosteroids	Categorical shared risk factor	At least one prescription of corticosteroids prior to baseline (past use). Derived from primary care prescription data.
Covariate	Bisphosphonate use	Categorical shared risk factor	At least one record of bisphosphonate prescription prior to baseline (past use). Derived from primary care prescription data.
Covariate	Autoimmune disorders	Categorical shared risk factor. Included: coeliac disease, inflammatory bowel disease, systemic lupus erythematosus, and rheumatoid arthritis.	ICD-10 code in the Hospital Episodes Statistics - Admitted Patient Care database, or Read/SNOMED/EMIS-coded record of in the CPRD primary care databases.
Covariate	Chronic kidney disease	Categorical shared risk factor	ICD-10 code in the Hospital Episodes Statistics - Admitted Patient Care database, or Read/SNOMED/EMIS-coded record of in the CPRD primary care databases. Serum creatinine levels used to calculate an estimated glomerular filtration rate.
Covariate	Liver disease	Categorical shared risk factor	ICD-10 code in the Hospital Episodes Statistics - Admitted Patient Care database, or Read/SNOMED/EMIS-coded record of in the CPRD primary care databases.
Covariate	Epilepsy	Categorical shared risk factor	ICD-10 code in the Hospital Episodes Statistics - Admitted Patient Care database, or Read/SNOMED/EMIS-coded record of in the CPRD primary care databases.

Table S2: Characteristics of cancer survivors and matched controls from the general population in CPRD Aurum and GOLD databases separately (all cancer sites).*

	CPRD Aurum				CPRD GOLD			
	Cancer survivors		Cancer-free controls		Cancer survivors		Cancer-free controls	
**All participants**	514,300		277,6693		63,860		449,711	
**Time from index date† to end of follow-up (years)**								
Mean (SD)	5.85 (4.47)		7.03 (4.78)		5.05 (3.68)		5.92 (3.91)	
Median (IQR)	4.45 (2.26- 8.28)		5.86 (3.09-10.00)		3.95 (2.12- 7.03)		4.98 (2.76- 8.27)	
Range	1.00-21.07		1.00-21.08		1.00-21.00		1.00-21.08	
**Total person-years ‡**								
	3008594.2		19521014		322536.59		2662569	
**Age (years)**								
Mean (SD)	65.65 (13.79)		65.73 (13.68)		66.23 (13.42)		68.05 (13.26)	
Median (IQR)	67 (57-76)		67 (57-76)		67 (58-76)		69 (60-78)	
**Age group at cancer diagnosis (years)**								
18 - 59	156,202	(30.37)	831,944	(29.96)	18,404	(28.82)	110,036	(24.47)
60 - 79	276,920	(53.84)	1,508,566	(54.33)	35,015	(54.83)	247,849	(55.11)
≥80	81,178	(15.78)	436,183	(15.71)	10,441	(16.35)	91,826	(20.42)
**Sex**								
Women	267,807	(52.07)	1,434,985	(51.68)	33,150	(51.91)	224,299	(49.88)
Men	246,493	(47.93)	1,341,708	(48.32)	30,710	(48.09)	225,412	(50.12)
**Patient-level Index of Multiple Deprivation (practice postcode-based)**								
1 (least deprived)	125,532	(24.41)	665,735	(23.98)	15,773	(24.70)	104,317	(23.20)
2	117,885	(22.92)	626,889	(22.58)	15,656	(24.52)	107,827	(23.98)
3	101,353	(19.71)	549,719	(19.80)	13,602	(21.30)	97,019	(21.57)
4	91,018	(17.70)	499,362	(17.98)	11,715	(18.34)	85,370	(18.98)
5 (most deprived)	78,063	(15.18)	432,418	(15.57)	7,090	(11.10)	55,023	(12.24)
**Calendar year of cancer diagnosis**								
1999 - 2003	100,327	(19.51)	539,564	(19.43)	13,529	(21.19)	100,288	(22.30)
2004 - 2008	120,036	(23.34)	647,870	(23.33)	21,647	(33.90)	153,865	(34.21)
2009 - 2013	136,855	(26.61)	740,803	(26.68)	19,303	(30.23)	132,952	(29.56)
2014 - 2019	157,082	(30.54)	848,456	(30.56)	9,381	(14.69)	62,606	(13.92)
**Ethnicity**								
White	342,261	(66.55)	1,895,041	(68.25)	27,590	(43.20)	195,089	(43.38)
South Asian	9,968	(1.94)	77,656	(2.80)	453	(0.71)	4,511	(1.00)
Black	9,863	(1.92)	51,824	(1.87)	366	(0.57)	2,531	(0.56)
Other	4,627	(0.90)	30,244	(1.09)	197	(0.31)	1,868	(0.42)
Unknown	147,581	(28.70)	721,928	(26.00)	35,254	(55.21)	245,712	(54.64)
**Body mass index**								
Underweight (<18.5)	10,052	(1.95)	45,761	(1.65)	990	(1.55)	4,884	(1.09)
Normal weight (18.5-24.9)	174,762	(33.98)	916,116	(32.99)	21,765	(34.08)	110,778	(24.63)
Overweight (25.0-29.9)	182,392	(35.46)	984,665	(35.46)	23,204	(36.34)	115,077	(25.59)
Obese (≥30.0)	115,975	(22.55)	630,840	(22.72)	13,802	(21.61)	67,468	(15.00)
Unknown	31,119	(6.05)	199,311	(7.18)	4,099	(6.42)	151,504	(33.69)
**Alcohol consumption**								
Non-drinker	48,339	(9.40)	278,474	(10.03)	6,383	(10.00)	38,652	(8.59)
Current drinker	275,389	(53.55)	1,484,754	(53.47)	48,042	(75.23)	236,780	(52.65)

Ex-drinker	20,543	(3.99)	101,626	(3.66)	4,905	(7.68)	22,050	(4.90)
Unknown	170,029	(33.06)	911,839	(32.84)	4,530	(7.09)	152,229	(33.85)
**Problem drinker**								
	15,525	(3.02)	82,232	(2.96)	1,414	(2.21)	6,808	(1.51)
**Smoking status**								
Non-smoker	155,324	(30.20)	868,699	(31.29)	28,256	(44.25)	206,547	(45.93)
Current smoker	70,104	(13.63)	503,736	(18.14)	10,548	(16.52)	70,385	(15.65)
Ex-smoker	280,733	(54.59)	1,338,278	(48.20)	23,789	(37.25)	158,974	(35.35)
Unknown	8,139	(1.58)	65,980	(2.38)	1,267	(1.98)	13,805	(3.07)
**Prescribed**								
Oral corticosteroids	110,984	(21.58)	468,239	(16.86)	10,957	(17.16)	70,582	(15.69)
Hormone replacement therapy	103,482	(20.12)	502,754	(18.11)	31	(0.05)	216	(0.05)
Bisphosphonate therapy	14,457	(2.81)	66,853	(2.41)	3,120	(4.89)	22,495	(5.00)
**History of**								
Fracture §	114,746	(22.31)	591,156	(21.29)	11,275	(17.66)	81,032	(18.02)
Osteoporosis	29,064	(5.65)	132,894	(4.79)	2,311	(3.62)	17,447	(3.88)
Eating disorder	2,142	(0.42)	10,120	(0.36)	275	(0.43)	1,914	(0.43)
**Comorbidities**								
Coeliac disease	2,146	(0.42)	11,166	(0.40)	179	(0.28)	1,287	(0.29)
Inflammatory bowel disease	28,666	(5.57)	98,718	(3.56)	879	(1.38)	5,749	(1.28)
Systemic lupus erythematosus	1,317	(0.26)	5,873	(0.21)	135	(0.21)	756	(0.17)
Rheumatoid arthritis	11,685	(2.27)	53,503	(1.93)	979	(1.53)	7,063	(1.57)
Chronic kidney disease	132,533	(25.77)	604,856	(21.78)	8,035	(12.58)	53,267	(11.84)
Liver disease	13,145	(2.56)	29,719	(1.07)	310	(0.49)	1,402	(0.31)
Epilepsy	11,145	(2.17)	48,851	(1.76)	1,047	(1.64)	6,783	(1.51)

SD = standard deviation; IQR = interquartile range; CPRD = Clinical Practice Research Datalink.
* Data are n (%) unless otherwise stated. The characteristics of the study participants were measured prior to or near the index date†. When information on body mass index, height and weight, alcohol consumption, and smoking status was unavailable prior to the index date, we used information recorded at any point in the clinical record. We ran a sensitivity analysis using only pre-index data.
† For cancer survivors, the index date was the date of their cancer diagnosis. In the matched cancer-free cohort, the matched cancer survivor's index date was allocated to each individual. Cancer-free people were individually matched on year of birth (+/- 3 years), sex and general practice to participants in the cancer survivor cohort.
‡ Index date to end of follow-up.
§ In adulthood.

Table S3: Characteristics of cancer survivors and matched controls from the general population (oral cavity cancer; ICD-10 C00-06).*

	Cancer survivors		Cancer-free controls	
**All participants**	5,266		31,631	
**Time from index date† to end of follow-up (years)**				
Mean (SD)	5.66 (4.28)		6.97 (4.73)	
Median (IQR)	4.40 (2.23- 8.04)		5.81 (3.10- 9.86)	
Range	1.00-21.06		1.00-21.07	
**Total person-years ‡**	29811.78		220388.12	
**Age (years)**				
Mean (SD)	62.61 (13.68)		62.70 (13.37)	
Median (IQR)	62 (53-72)		62 (54-72)	
**Age group at cancer diagnosis (years)**				
18 - 59	2,189	(41.57)	13,128	(41.50)
60 - 79	2,446	(46.45)	14,807	(46.81)
≥80	631	(11.98)	3,696	(11.68)
**Sex**				
Women	2,093	(39.75)	12,280	(38.82)
Men	3,173	(60.25)	19,351	(61.18)
**Patient-level Index of Multiple Deprivation (practice postcode-based)**				
1 (least deprived)	1,045	(19.84)	6,790	(21.47)
2	1,101	(20.91)	6,789	(21.46)
3	997	(18.93)	6,192	(19.58)
4	1,053	(20.00)	6,158	(19.47)
5 (most deprived)	1,070	(20.32)	5,665	(17.91)
**Calendar year of cancer diagnosis**				
1999 - 2003	1,042	(19.79)	6,074	(19.20)
2004 - 2008	1,226	(23.28)	7,368	(23.29)
2009 - 2013	1,479	(28.09)	8,966	(28.35)
2014 - 2019	1,519	(28.85)	9,223	(29.16)
**Ethnicity**				
White	3,305	(62.76)	20,358	(64.36)
South Asian	181	(3.44)	1,004	(3.17)
Black	43	(0.82)	530	(1.68)
Other	24	(0.46)	365	(1.15)
Unknown	1,713	(32.53)	9,374	(29.64)
**Body mass index**				
Underweight (<18.5)	242	(4.60)	437	(1.38)
Normal weight (18.5-24.9)	2,068	(39.27)	9,337	(29.52)
Overweight (25.0-29.9)	1,659	(31.50)	11,066	(34.98)
Obese (≥30.0)	883	(16.77)	7,088	(22.41)
Unknown	414	(7.86)	3,703	(11.71)
**Alcohol consumption**				
Non-drinker	437	(8.30)	2,885	(9.12)
Current drinker	3,226	(61.26)	17,380	(54.95)
Ex-drinker	274	(5.20)	1,123	(3.55)

Unknown	1,329	(25.24)	10,243	(32.38)
**Problem drinker**				
	572	(10.86)	1,158	(3.66)
**Smoking status**				
Non-smoker	1,237	(23.49)	10,357	(32.74)
Current smoker	1,165	(22.12)	6,097	(19.28)
Ex-smoker	2,757	(52.35)	14,216	(44.94)
Unknown	107	(2.03)	961	(3.04)
**Prescribed**				
Oral corticosteroids	1,212	(23.02)	4,756	(15.04)
Hormone replacement therapy	630	(11.96)	3,633	(11.49)
Bisphosphonate therapy	153	(2.91)	658	(2.08)
**History of**				
Fracture §	1,474	(27.99)	6,844	(21.64)
Osteoporosis	260	(4.94)	1,154	(3.65)
Eating disorder	33	(0.63)	89	(0.28)
**Comorbidities**				
Coeliac disease	28	(0.53)	108	(0.34)
Inflammatory bowel disease	256	(4.86)	1,018	(3.22)
Systemic lupus erythematosus	20	(0.38)	53	(0.17)
Rheumatoid arthritis	146	(2.77)	484	(1.53)
Chronic kidney disease	893	(16.96)	4,903	(15.50)
Liver disease	250	(4.75)	338	(1.07)
Epilepsy	186	(3.53)	561	(1.77)

SD = standard deviation; IQR = interquartile range.
* Data are n (%) unless otherwise stated. The characteristics of the study participants were measured prior to or near the index date†. When information on body mass index, height and weight, alcohol consumption, and smoking status was unavailable prior to the index date, we used information recorded at any point in the clinical record. We ran a sensitivity analysis using only pre-index data.
† For cancer survivors, the index date was the date of their cancer diagnosis. In the matched cancer-free cohort, the matched cancer survivor's index date was allocated to each individual. Cancer-free people were individually matched on year of birth (+/- 3 years), sex and general practice to participants in the cancer survivor cohort.
‡ Index date to end of follow-up.
§ In adulthood.

Table S4: Characteristics of cancer survivors and matched controls from the general population (oesophageal cancer; ICD-10 C15).*

	Cancer survivors		Cancer-free controls	
**All participants**	8,048		66,543	
**Time from index date† to end of follow-up (years)**				
Mean (SD)	3.60 (3.49)		6.83 (4.57)	
Median (IQR)	2.10 (1.38- 4.35)		5.75 (3.08- 9.64)	
Range	1.00-20.96		1.00-21.07	
**Total person-years ‡**	29004.864		454276.05	
**Age (years)**				
Mean (SD)	68.39 (11.38)		68.53 (11.28)	
Median (IQR)	69 (61-77)		69 (61-77)	
**Age group at cancer diagnosis (years)**				
18 - 59	1,749	(21.73)	14,034	(21.09)
60 - 79	4,880	(60.64)	40,678	(61.13)
≥80	1,419	(17.63)	11,831	(17.78)
**Sex**				
Women	2,676	(33.25)	20,174	(30.32)
Men	5,372	(66.75)	46,369	(69.68)
**Patient-level Index of Multiple Deprivation (practice postcode-based)**				
1 (least deprived)	1,719	(21.36)	14,999	(22.54)
2	1,761	(21.88)	15,165	(22.79)
3	1,635	(20.32)	13,573	(20.40)
4	1,566	(19.46)	12,487	(18.77)
5 (most deprived)	1,363	(16.94)	10,267	(15.43)
**Calendar year of cancer diagnosis**				
1999 - 2003	1,695	(21.06)	13,773	(20.70)
2004 - 2008	2,112	(26.24)	17,703	(26.60)
2009 - 2013	2,205	(27.40)	18,185	(27.33)
2014 - 2019	2,036	(25.30)	16,882	(25.37)
**Ethnicity**				
White	4,236	(52.63)	41,579	(62.48)
South Asian	88	(1.09)	1,215	(1.83)
Black	47	(0.58)	763	(1.15)
Other	27	(0.34)	467	(0.70)
Unknown	3,650	(45.35)	22,519	(33.84)
**Body mass index**				
Underweight (<18.5)	461	(5.73)	862	(1.30)
Normal weight (18.5-24.9)	3,113	(38.68)	18,558	(27.89)
Overweight (25.0-29.9)	2,527	(31.40)	23,523	(35.35)
Obese (≥30.0)	1,229	(15.27)	13,678	(20.56)
Unknown	718	(8.92)	9,922	(14.91)
**Alcohol consumption**				
Non-drinker	661	(8.21)	5,650	(8.49)
Current drinker	4,336	(53.88)	36,432	(54.75)
Ex-drinker	443	(5.50)	2,396	(3.60)
Unknown	2,608	(32.41)	22,065	(33.16)

**Problem drinker**				
	413	(5.13)	1,969	(2.96)
**Smoking status**				
Non-smoker	1,829	(22.73)	20,928	(31.45)
Current smoker	1,331	(16.54)	12,136	(18.24)
Ex-smoker	4,606	(57.23)	31,532	(47.39)
Unknown	282	(3.50)	1,947	(2.93)
**Prescribed**				
Oral corticosteroids	2,126	(26.42)	11,040	(16.59)
Hormone replacement therapy	763	(9.48)	5,011	(7.53)
Bisphosphonate therapy	265	(3.29)	1,877	(2.82)
**History of**				
Fracture §	2,064	(25.65)	14,248	(21.41)
Osteoporosis	423	(5.26)	2,574	(3.87)
Eating disorder	80	(0.99)	145	(0.22)
**Comorbidities**				
Coeliac disease	63	(0.78)	235	(0.35)
Inflammatory bowel disease	592	(7.36)	2,001	(3.01)
Systemic lupus erythematosus	25	(0.31)	98	(0.15)
Rheumatoid arthritis	215	(2.67)	1,175	(1.77)
Chronic kidney disease	1,979	(24.59)	13,546	(20.36)
Liver disease	359	(4.46)	623	(0.94)
Epilepsy	176	(2.19)	1,181	(1.77)

SD = standard deviation; IQR = interquartile range.
* Data are n (%) unless otherwise stated. The characteristics of the study participants were measured prior to or near the index date†. When information on body mass index, height and weight, alcohol consumption, and smoking status was unavailable prior to the index date, we used information recorded at any point in the clinical record. We ran a sensitivity analysis using only pre-index data.
† For cancer survivors, the index date was the date of their cancer diagnosis. In the matched cancer-free cohort, the matched cancer survivor's index date was allocated to each individual. Cancer-free people were individually matched on year of birth (+/- 3 years), sex and general practice to participants in the cancer survivor cohort.
‡ Index date to end of follow-up.
§ In adulthood.

Table S5: Characteristics of cancer survivors and matched controls from the general population (stomach cancer; ICD-10 C16).*

	Cancer survivors		Cancer-free controls	
**All participants**	5,436		41,253	
**Time from index date† to end of follow-up (years)**				
Mean (SD)	4.26 (3.91)		6.88 (4.56)	
Median (IQR)	2.57 (1.51- 5.59)		5.81 (3.15- 9.66)	
Range	1.00-21.01		1.00-21.08	
**Total person-years ‡**				
	23163.535		283697.26	
**Age (years)**				
Mean (SD)	70.75 (12.46)		70.69 (12.41)	
Median (IQR)	73 (64-80)		72 (64-80)	
**Age group at cancer diagnosis (years)**				
18 - 59	946	(17.40)	7,271	(17.63)
60 - 79	3,111	(57.23)	23,554	(57.10)
≥80	1,379	(25.37)	10,428	(25.28)
**Sex**				
Women	2,134	(39.26)	15,673	(37.99)
Men	3,302	(60.74)	25,580	(62.01)
**Patient-level Index of Multiple Deprivation (practice postcode-based)**				
1 (least deprived)	963	(17.72)	8,227	(19.94)
2	1,107	(20.36)	8,577	(20.79)
3	1,076	(19.79)	8,158	(19.78)
4	1,109	(20.40)	8,257	(20.02)
5 (most deprived)	1,172	(21.56)	8,014	(19.43)
**Calendar year of cancer diagnosis**				
1999 - 2003	1,408	(25.90)	10,339	(25.06)
2004 - 2008	1,436	(26.42)	11,582	(28.08)
2009 - 2013	1,405	(25.85)	10,824	(26.24)
2014 - 2019	1,187	(21.84)	8,508	(20.62)
**Ethnicity**				
White	2,831	(52.08)	24,567	(59.55)
South Asian	122	(2.24)	1,015	(2.46)
Black	169	(3.11)	765	(1.85)
Other	67	(1.23)	388	(0.94)
Unknown	2,247	(41.34)	14,518	(35.19)
**Body mass index**				
Underweight (<18.5)	278	(5.11)	650	(1.58)
Normal weight (18.5-24.9)	2,255	(41.48)	11,999	(29.09)
Overweight (25.0-29.9)	1,613	(29.67)	13,881	(33.65)
Obese (≥30.0)	796	(14.64)	8,223	(19.93)
Unknown	494	(9.09)	6,500	(15.76)
**Alcohol consumption**				
Non-drinker	600	(11.04)	4,020	(9.74)
Current drinker	2,779	(51.12)	21,536	(52.20)
Ex-drinker	314	(5.78)	1,595	(3.87)

Unknown	1,743	(32.06)	14,102	(34.18)
**Problem drinker**				
	159	(2.92)	1,109	(2.69)
**Smoking status**				
Non-smoker	1,389	(25.55)	13,185	(31.96)
Current smoker	840	(15.45)	7,183	(17.41)
Ex-smoker	2,983	(54.87)	19,465	(47.18)
Unknown	224	(4.12)	1,420	(3.44)
**Prescribed**				
Oral corticosteroids	1,286	(23.66)	7,025	(17.03)
Hormone replacement therapy	514	(9.46)	3,415	(8.28)
Bisphosphonate therapy	202	(3.72)	1,395	(3.38)
**History of**				
Fracture §	1,182	(21.74)	8,534	(20.69)
Osteoporosis	314	(5.78)	1,949	(4.72)
Eating disorder	29	(0.53)	98	(0.24)
**Comorbidities**				
Coeliac disease	31	(0.57)	134	(0.32)
Inflammatory bowel disease	413	(7.60)	1,239	(3.00)
Systemic lupus erythematosus	9	(0.17)	71	(0.17)
Rheumatoid arthritis	151	(2.78)	801	(1.94)
Chronic kidney disease	1,743	(32.06)	10,207	(24.74)
Liver disease	274	(5.04)	361	(0.88)
Epilepsy	118	(2.17)	682	(1.65)

SD = standard deviation; IQR = interquartile range.

* Data are n (%) unless otherwise stated. The characteristics of the study participants were measured prior to or near the index date†. When information on body mass index, height and weight, alcohol consumption, and smoking status was unavailable prior to the index date, we used information recorded at any point in the clinical record. We ran a sensitivity analysis using only pre-index data.

† For cancer survivors, the index date was the date of their cancer diagnosis. In the matched cancer-free cohort, the matched cancer survivor's index date was allocated to each individual. Cancer-free people were individually matched on year of birth (+/- 3 years), sex and general practice to participants in the cancer survivor cohort.

‡ Index date to end of follow-up.

§ In adulthood.

Table S6: Characteristics of cancer survivors and matched controls from the general population (colorectal cancer; ICD-10 C18-20).*

	Cancer survivors		Cancer-free controls	
**All participants**	67,876		384,129	
**Time from index date† to end of follow-up (years)**				
Mean (SD)	5.50 (4.30)		6.83 (4.63)	
Median (IQR)	4.02 (2.11- 7.79)		5.71 (3.03- 9.68)	
Range	1.00-21.07		1.00-21.07	
**Total person-years ‡**				
	372993.82		2624778.6	
**Age (years)**				
Mean (SD)	69.19 (12.19)		69.34 (12.18)	
Median (IQR)	70 (62-78)		71 (62-78)	
**Age group at cancer diagnosis (years)**				
18 - 59	13,616	(20.06)	76,038	(19.79)
60 - 79	40,011	(58.95)	225,693	(58.75)
≥80	14,249	(20.99)	82,398	(21.45)
**Sex**				
Women	29,761	(43.85)	169,123	(44.03)
Men	38,115	(56.15)	215,006	(55.97)
**Patient-level Index of Multiple Deprivation (practice postcode-based)**				
1 (least deprived)	16,381	(24.13)	90,906	(23.67)
2	15,885	(23.40)	88,912	(23.15)
3	13,727	(20.22)	77,925	(20.29)
4	12,069	(17.78)	69,091	(17.99)
5 (most deprived)	9,745	(14.36)	56,967	(14.83)
**Calendar year of cancer diagnosis**				
1999 - 2003	14,462	(21.31)	81,744	(21.28)
2004 - 2008	17,358	(25.57)	99,818	(25.99)
2009 - 2013	18,337	(27.02)	103,761	(27.01)
2014 - 2019	17,719	(26.10)	98,806	(25.72)
**Ethnicity**				
White	41,859	(61.67)	243,935	(63.50)
South Asian	895	(1.32)	8,176	(2.13)
Black	713	(1.05)	5,079	(1.32)
Other	456	(0.67)	2,980	(0.78)
Unknown	23,953	(35.29)	123,959	(32.27)
**Body mass index**				
Underweight (<18.5)	1,433	(2.11)	6,072	(1.58)
Normal weight (18.5-24.9)	23,144	(34.10)	117,858	(30.68)
Overweight (25.0-29.9)	24,429	(35.99)	134,432	(35.00)
Obese (≥30.0)	14,196	(20.91)	79,690	(20.75)
Unknown	4,674	(6.89)	46,077	(12.00)
**Alcohol consumption**				
Non-drinker	6,039	(8.90)	37,150	(9.67)
Current drinker	38,476	(56.69)	204,946	(53.35)
Ex-drinker	2,888	(4.25)	14,929	(3.89)

Unknown	20,473	(30.16)	127,104	(33.09)
**Problem drinker**				
	2,103	(3.10)	10,565	(2.75)
**Smoking status**				
Non-smoker	21,153	(31.16)	124,771	(32.48)
Current smoker	8,387	(12.36)	66,633	(17.35)
Ex-smoker	36,983	(54.49)	182,037	(47.39)
Unknown	1,353	(1.99)	10,688	(2.78)
**Prescribed**				
Oral corticosteroids	13,176	(19.41)	65,765	(17.12)
Hormone replacement therapy	8,477	(12.49)	45,443	(11.83)
Bisphosphonate therapy	2,233	(3.29)	12,690	(3.30)
**History of**				
Fracture §	15,384	(22.66)	83,954	(21.86)
Osteoporosis	3,512	(5.17)	19,755	(5.14)
Eating disorder	227	(0.33)	1,050	(0.27)
**Comorbidities**				
Coeliac disease	300	(0.44)	1,383	(0.36)
Inflammatory bowel disease	5,579	(8.22)	12,584	(3.28)
Systemic lupus erythematosus	137	(0.20)	755	(0.20)
Rheumatoid arthritis	1,415	(2.08)	7,596	(1.98)
Chronic kidney disease	18,618	(27.43)	91,994	(23.95)
Liver disease	2,433	(3.58)	3,815	(0.99)
Epilepsy	1,361	(2.01)	6,554	(1.71)

SD = standard deviation; IQR = interquartile range.
* Data are n (%) unless otherwise stated. The characteristics of the study participants were measured prior to or near the index date†. When information on body mass index, height and weight, alcohol consumption, and smoking status was unavailable prior to the index date, we used information recorded at any point in the clinical record. We ran a sensitivity analysis using only pre-index data.
† For cancer survivors, the index date was the date of their cancer diagnosis. In the matched cancer-free cohort, the matched cancer survivor's index date was allocated to each individual. Cancer-free people were individually matched on year of birth (+/- 3 years), sex and general practice to participants in the cancer survivor cohort.
‡ Index date to end of follow-up.
§ In adulthood.

Table S7: Characteristics of cancer survivors and matched controls from the general population (liver cancer; ICD-10 C22).*

	Cancer survivors		Cancer-free controls	
**All participants**	3,420		25,502	
**Time from index date† to end of follow-up (years)**				
Mean (SD)	3.33 (2.93)		5.96 (4.16)	
Median (IQR)	2.20 (1.44- 4.02)		4.89 (2.65- 8.19)	
Range	1.00-21.01		1.00-21.07	
**Total person-years ‡**				
	11396.18		152071.5	
**Age (years)**				
Mean (SD)	65.90 (12.67)		67.16 (12.62)	
Median (IQR)	67 (58-75)		68 (59-76)	
**Age group at cancer diagnosis (years)**				
18 - 59	1,009	(29.50)	6,707	(26.30)
60 - 79	1,938	(56.67)	14,557	(57.08)
≥80	473	(13.83)	4,238	(16.62)
**Sex**				
Women	1,052	(30.76)	8,512	(33.38)
Men	2,368	(69.24)	16,990	(66.62)
**Patient-level Index of Multiple Deprivation (practice postcode-based)**				
1 (least deprived)	623	(18.22)	5,237	(20.54)
2	653	(19.09)	5,314	(20.84)
3	669	(19.56)	4,938	(19.36)
4	671	(19.62)	5,057	(19.83)
5 (most deprived)	804	(23.51)	4,943	(19.38)
**Calendar year of cancer diagnosis**				
1999 - 2003	390	(11.40)	3,305	(12.96)
2004 - 2008	654	(19.12)	5,516	(21.63)
2009 - 2013	992	(29.01)	7,605	(29.82)
2014 - 2019	1,384	(40.47)	9,076	(35.59)
**Ethnicity**				
White	1,918	(56.08)	16,067	(63.00)
South Asian	210	(6.14)	959	(3.76)
Black	116	(3.39)	713	(2.80)
Other	77	(2.25)	345	(1.35)
Unknown	1,099	(32.13)	7,418	(29.09)
**Body mass index**				
Underweight (<18.5)	80	(2.34)	326	(1.28)
Normal weight (18.5-24.9)	1,103	(32.25)	7,165	(28.10)
Overweight (25.0-29.9)	1,165	(34.06)	8,747	(34.30)
Obese (≥30.0)	849	(24.82)	5,382	(21.10)
Unknown	223	(6.52)	3,882	(15.22)
**Alcohol consumption**				
Non-drinker	302	(8.83)	2,151	(8.43)
Current drinker	1,989	(58.16)	13,817	(54.18)
Ex-drinker	308	(9.01)	1,080	(4.23)

Unknown	821	(24.01)	8,454	(33.15)
**Problem drinker**				
	436	(12.75)	846	(3.32)
**Smoking status**				
Non-smoker	778	(22.75)	8,251	(32.35)
Current smoker	575	(16.81)	4,698	(18.42)
Ex-smoker	2,004	(58.60)	11,896	(46.65)
Unknown	63	(1.84)	657	(2.58)
**Prescribed**				
Oral corticosteroids	1,002	(29.30)	4,260	(16.70)
Hormone replacement therapy	339	(9.91)	2,242	(8.79)
Bisphosphonate therapy	99	(2.89)	683	(2.68)
**History of**				
Fracture §	899	(26.29)	5,590	(21.92)
Osteoporosis	224	(6.55)	1,091	(4.28)
Eating disorder	13	(0.38)	64	(0.25)
**Comorbidities**				
Coeliac disease	24	(0.70)	115	(0.45)
Inflammatory bowel disease	313	(9.15)	816	(3.20)
Systemic lupus erythematosus	17	(0.50)	40	(0.16)
Rheumatoid arthritis	80	(2.34)	433	(1.70)
Chronic kidney disease	1,006	(29.42)	5,257	(20.61)
Liver disease	2,076	(60.70)	292	(1.15)
Epilepsy	110	(3.22)	454	(1.78)

SD = standard deviation; IQR = interquartile range.
* Data are n (%) unless otherwise stated. The characteristics of the study participants were measured prior to or near the index date†. When information on body mass index, height and weight, alcohol consumption, and smoking status was unavailable prior to the index date, we used information recorded at any point in the clinical record. We ran a sensitivity analysis using only pre-index data.
† For cancer survivors, the index date was the date of their cancer diagnosis. In the matched cancer-free cohort, the matched cancer survivor's index date was allocated to each individual. Cancer-free people were individually matched on year of birth (+/- 3 years), sex and general practice to participants in the cancer survivor cohort.
‡ Index date to end of follow-up.
§ In adulthood.

Table S8: Characteristics of cancer survivors and matched controls from the general population (pancreatic cancer; ICD-10 C25).*

	Cancer survivors		Cancer-free controls	
**All participants**	4,198		35,778	
**Time from index date† to end of follow-up (years)**				
Mean (SD)	3.20 (3.15)		6.43 (4.40)	
Median (IQR)	1.88 (1.30- 3.64)		5.26 (2.88- 9.04)	
Range	1.00-20.84		1.00-21.07	
**Total person-years ‡**				
	13421.02		230145.7	
**Age (years)**				
Mean (SD)	67.09 (12.66)		68.75 (12.22)	
Median (IQR)	68 (59-76)		70 (61-77)	
**Age group at cancer diagnosis (years)**				
18 - 59	1,083	(25.80)	7,661	(21.41)
60 - 79	2,433	(57.96)	21,078	(58.91)
≥80	682	(16.25)	7,039	(19.67)
**Sex**				
Women	2,110	(50.26)	17,740	(49.58)
Men	2,088	(49.74)	18,038	(50.42)
**Patient-level Index of Multiple Deprivation (practice postcode-based)**				
1 (least deprived)	979	(23.32)	8,325	(23.27)
2	1,005	(23.94)	8,207	(22.94)
3	841	(20.03)	7,471	(20.88)
4	743	(17.70)	6,674	(18.65)
5 (most deprived)	627	(14.94)	5,079	(14.20)
**Calendar year of cancer diagnosis**				
1999 - 2003	720	(17.15)	6,506	(18.18)
2004 - 2008	1,047	(24.94)	10,108	(28.25)
2009 - 2013	1,105	(26.32)	9,770	(27.31)
2014 - 2019	1,326	(31.59)	9,394	(26.26)
**Ethnicity**				
White	2,175	(51.81)	21,313	(59.57)
South Asian	82	(1.95)	825	(2.31)
Black	74	(1.76)	520	(1.45)
Other	41	(0.98)	271	(0.76)
Unknown	1,826	(43.50)	12,849	(35.91)
**Body mass index**				
Underweight (<18.5)	201	(4.79)	537	(1.50)
Normal weight (18.5-24.9)	1,834	(43.69)	10,318	(28.84)
Overweight (25.0-29.9)	1,210	(28.82)	11,272	(31.51)
Obese (≥30.0)	623	(14.84)	6,921	(19.34)
Unknown	330	(7.86)	6,730	(18.81)
**Alcohol consumption**				
Non-drinker	388	(9.24)	3,421	(9.56)
Current drinker	2,182	(51.98)	18,308	(51.17)
Ex-drinker	235	(5.60)	1,570	(4.39)

Unknown	1,393	(33.18)	12,479	(34.88)
**Problem drinker**				
	141	(3.36)	879	(2.46)
**Smoking status**				
Non-smoker	1,168	(27.82)	12,965	(36.24)
Current smoker	670	(15.96)	5,795	(16.20)
Ex-smoker	2,233	(53.19)	16,063	(44.90)
Unknown	127	(3.03)	955	(2.67)
**Prescribed**				
Oral corticosteroids	1,113	(26.51)	6,085	(17.01)
Hormone replacement therapy	705	(16.79)	4,012	(11.21)
Bisphosphonate therapy	149	(3.55)	1,466	(4.10)
**History of**				
Fracture §	997	(23.75)	7,588	(21.21)
Osteoporosis	228	(5.43)	1,949	(5.45)
Eating disorder	32	(0.76)	151	(0.42)
**Comorbidities**				
Coeliac disease	58	(1.38)	137	(0.38)
Inflammatory bowel disease	421	(10.03)	1,035	(2.89)
Systemic lupus erythematosus	17	(0.40)	69	(0.19)
Rheumatoid arthritis	97	(2.31)	720	(2.01)
Chronic kidney disease	1,048	(24.96)	7,233	(20.22)
Liver disease	381	(9.08)	309	(0.86)
Epilepsy	82	(1.95)	612	(1.71)

SD = standard deviation; IQR = interquartile range.
* Data are n (%) unless otherwise stated. The characteristics of the study participants were measured prior to or near the index date†. When information on body mass index, height and weight, alcohol consumption, and smoking status was unavailable prior to the index date, we used information recorded at any point in the clinical record. We ran a sensitivity analysis using only pre-index data.
† For cancer survivors, the index date was the date of their cancer diagnosis. In the matched cancer-free cohort, the matched cancer survivor's index date was allocated to each individual. Cancer-free people were individually matched on year of birth (+/- 3 years), sex and general practice to participants in the cancer survivor cohort.
‡ Index date to end of follow-up.
§ In adulthood.

Table S9: Characteristics of cancer survivors and matched controls from the general population (lung cancer; ICD-10 C34).*

	Cancer survivors		Cancer-free controls	
**All participants**	32,039		211,485	
**Time from index date† to end of follow-up (years)**				
Mean (SD)	3.22 (2.91)		6.51 (4.46)	
Median (IQR)	2.09 (1.39- 3.81)		5.38 (2.90- 9.11)	
Range	1.00-21.06		1.00-21.07	
**Total person-years ‡**	103267.7		1375849	
**Age (years)**				
Mean (SD)	69.44 (10.63)		69.93 (10.65)	
Median (IQR)	70 (63-77)		71 (63-78)	
**Age group at cancer diagnosis (years)**				
18 - 59	5,446	(17.00)	34,079	(16.11)
60 - 79	21,063	(65.74)	137,621	(65.07)
≥80	5,530	(17.26)	39,785	(18.81)
**Sex**				
Women	15,187	(47.40)	97,588	(46.14)
Men	16,852	(52.60)	113,897	(53.86)
**Patient-level Index of Multiple Deprivation (practice postcode-based)**				
1 (least deprived)	5,126	(16.00)	40,373	(19.09)
2	5,813	(18.14)	43,483	(20.56)
3	6,121	(19.10)	42,185	(19.95)
4	6,936	(21.65)	43,028	(20.35)
5 (most deprived)	8,012	(25.01)	42,238	(19.97)
**Calendar year of cancer diagnosis**				
1999 - 2003	5,860	(18.29)	41,905	(19.81)
2004 - 2008	7,196	(22.46)	52,632	(24.89)
2009 - 2013	8,669	(27.06)	57,441	(27.16)
2014 - 2019	10,314	(32.19)	59,507	(28.14)
**Ethnicity**				
White	18,218	(56.86)	133,765	(63.25)
South Asian	422	(1.32)	4,582	(2.17)
Black	323	(1.01)	3,549	(1.68)
Other	228	(0.71)	1,821	(0.86)
Unknown	12,848	(40.10)	67,768	(32.04)
**Body mass index**				
Underweight (<18.5)	1,644	(5.13)	3,152	(1.49)
Normal weight (18.5-24.9)	12,386	(38.66)	60,808	(28.75)
Overweight (25.0-29.9)	9,822	(30.66)	71,420	(33.77)
Obese (≥30.0)	5,541	(17.29)	45,499	(21.51)
Unknown	2,646	(8.26)	30,606	(14.47)
**Alcohol consumption**				
Non-drinker	2,938	(9.17)	20,584	(9.73)
Current drinker	16,922	(52.82)	115,331	(54.53)
Ex-drinker	1,955	(6.10)	9,722	(4.60)

Unknown	10,224	(31.91)	65,848	(31.14)
**Problem drinker**				
	1,452	(4.53)	5,610	(2.65)
**Smoking status**				
Non-smoker	2,662	(8.31)	68,286	(32.29)
Current smoker	7,849	(24.50)	37,744	(17.85)
Ex-smoker	20,689	(64.57)	100,199	(47.38)
Unknown	839	(2.62)	5,256	(2.49)
**Prescribed**				
Oral corticosteroids	14,913	(46.55)	38,449	(18.18)
Hormone replacement therapy	5,203	(16.24)	26,301	(12.44)
Bisphosphonate therapy	1,355	(4.23)	7,641	(3.61)
**History of**				
Fracture §	8,512	(26.57)	45,536	(21.53)
Osteoporosis	2,389	(7.46)	11,777	(5.57)
Eating disorder	188	(0.59)	662	(0.31)
**Comorbidities**				
Coeliac disease	134	(0.42)	780	(0.37)
Inflammatory bowel disease	2,084	(6.50)	7,015	(3.32)
Systemic lupus erythematosus	150	(0.47)	410	(0.19)
Rheumatoid arthritis	1,249	(3.90)	4,568	(2.16)
Chronic kidney disease	9,321	(29.09)	49,582	(23.44)
Liver disease	1,019	(3.18)	2,225	(1.05)
Epilepsy	835	(2.61)	3,788	(1.79)

SD = standard deviation; IQR = interquartile range.

* Data are n (%) unless otherwise stated. The characteristics of the study participants were measured prior to or near the index date†. When information on body mass index, height and weight, alcohol consumption, and smoking status was unavailable prior to the index date, we used information recorded at any point in the clinical record. We ran a sensitivity analysis using only pre-index data.

† For cancer survivors, the index date was the date of their cancer diagnosis. In the matched cancer-free cohort, the matched cancer survivor's index date was allocated to each individual. Cancer-free people were individually matched on year of birth (+/- 3 years), sex and general practice to participants in the cancer survivor cohort.

‡ Index date to end of follow-up.

§ In adulthood.

Table S10: Characteristics of cancer survivors and matched controls from the general population (malignant melanoma; ICD-10 C43).*

	Cancer survivors		Cancer-free controls	
**All participants**	41,705		219,667	
**Time from index date† to end of follow-up (years)**				
Mean (SD)	6.44 (4.62)		6.86 (4.72)	
Median (IQR)	5.18 (2.69- 9.12)		5.64 (3.01- 9.76)	
Range	1.00-21.07		1.00-21.07	
**Total person-years ‡**	268725.2		1507573	
**Age (years)**				
Mean (SD)	59.92 (16.45)		60.19 (16.37)	
Median (IQR)	61 (48-73)		61 (48-73)	
**Age group at cancer diagnosis (years)**				
18 - 59	19,663	(47.15)	101,848	(46.36)
60 - 79	16,818	(40.33)	89,960	(40.95)
≥80	5,224	(12.53)	27,859	(12.68)
**Sex**				
Women	22,698	(54.43)	119,231	(54.28)
Men	19,007	(45.57)	100,436	(45.72)
**Patient-level Index of Multiple Deprivation (practice postcode-based)**				
1 (least deprived)	12,342	(29.59)	59,148	(26.93)
2	10,660	(25.56)	52,818	(24.04)
3	8,255	(19.79)	43,939	(20.00)
4	6,450	(15.47)	36,801	(16.75)
5 (most deprived)	3,964	(9.50)	26,765	(12.18)
**Calendar year of cancer diagnosis**				
1999 - 2003	6,676	(16.01)	35,393	(16.11)
2004 - 2008	9,900	(23.74)	52,275	(23.80)
2009 - 2013	11,912	(28.56)	62,550	(28.47)
2014 - 2019	13,217	(31.69)	69,449	(31.62)
**Ethnicity**				
White	29,740	(71.31)	145,898	(66.42)
South Asian	72	(0.17)	4,473	(2.04)
Black	68	(0.16)	2,564	(1.17)
Other	122	(0.29)	2,300	(1.05)
Unknown	11,703	(28.06)	64,432	(29.33)
**Body mass index**				
Underweight (<18.5)	546	(1.31)	3,666	(1.67)
Normal weight (18.5-24.9)	14,553	(34.90)	73,927	(33.65)
Overweight (25.0-29.9)	15,118	(36.25)	72,759	(33.12)
Obese (≥30.0)	9,105	(21.83)	46,894	(21.35)
Unknown	2,383	(5.71)	22,421	(10.21)
**Alcohol consumption**				
Non-drinker	3,078	(7.38)	19,910	(9.06)
Current drinker	23,741	(56.93)	114,286	(52.03)
Ex-drinker	1,460	(3.50)	7,559	(3.44)

Unknown	13,426	(32.19)	77,912	(35.47)
**Problem drinker**				
	1,117	(2.68)	6,671	(3.04)
**Smoking status**				
Non-smoker	16,198	(38.84)	76,936	(35.02)
Current smoker	4,869	(11.67)	40,015	(18.22)
Ex-smoker	20,176	(48.38)	97,712	(44.48)
Unknown	462	(1.11)	5,004	(2.28)
**Prescribed**				
Oral corticosteroids	6,576	(15.77)	33,918	(15.44)
Hormone replacement therapy	7,906	(18.96)	36,810	(16.76)
Bisphosphonate therapy	965	(2.31)	4,845	(2.21)
**History of**				
Fracture §	9,414	(22.57)	47,991	(21.85)
Osteoporosis	1,746	(4.19)	9,089	(4.14)
Eating disorder	223	(0.53)	1,014	(0.46)
**Comorbidities**				
Coeliac disease	169	(0.41)	865	(0.39)
Inflammatory bowel disease	1,421	(3.41)	6,722	(3.06)
Systemic lupus erythematosus	80	(0.19)	451	(0.21)
Rheumatoid arthritis	764	(1.83)	3,453	(1.57)
Chronic kidney disease	7,023	(16.84)	35,927	(16.36)
Liver disease	388	(0.93)	1,965	(0.89)
Epilepsy	664	(1.59)	3,681	(1.68)

SD = standard deviation; IQR = interquartile range.
* Data are n (%) unless otherwise stated. The characteristics of the study participants were measured prior to or near the index date†. When information on body mass index, height and weight, alcohol consumption, and smoking status was unavailable prior to the index date, we used information recorded at any point in the clinical record. We ran a sensitivity analysis using only pre-index data.
† For cancer survivors, the index date was the date of their cancer diagnosis. In the matched cancer-free cohort, the matched cancer survivor's index date was allocated to each individual. Cancer-free people were individually matched on year of birth (+/- 3 years), sex and general practice to participants in the cancer survivor cohort.
‡ Index date to end of follow-up.
§ In adulthood.

Table S11: Characteristics of cancer survivors and matched controls from the general population (breast cancer; ICD-10 C50).*

	Cancer survivors		Cancer-free controls	
**All participants**	145,515		742,221	
**Time from index date† to end of follow-up (years)**				
Mean (SD)	6.65 (4.68)		7.35 (4.95)	
Median (IQR)	5.39 (2.84- 9.45)		6.17 (3.24-10.55)	
Range	1.00-21.07		1.00-21.07	
**Total person-years ‡**				
	967671.8		5453912	
**Age (years)**				
Mean (SD)	61.71 (13.84)		61.82 (13.86)	
Median (IQR)	61 (51-71)		61 (51-72)	
**Age group at cancer diagnosis (years)**				
18 - 59	66,828	(45.93)	338,270	(45.58)
60 - 79	61,102	(41.99)	312,857	(42.15)
≥80	17,585	(12.08)	91,094	(12.27)
**Sex**				
Women	145,515	(100.00)	742,221	(100.00)
**Patient-level Index of Multiple Deprivation (practice postcode-based)**				
1 (least deprived)	36,758	(25.26)	182,741	(24.62)
2	33,905	(23.30)	169,926	(22.89)
3	29,003	(19.93)	147,670	(19.90)
4	25,642	(17.62)	132,953	(17.91)
5 (most deprived)	20,100	(13.81)	108,252	(14.58)
**Calendar year of cancer diagnosis**				
1999 - 2003	30,282	(20.81)	155,241	(20.92)
2004 - 2008	36,598	(25.15)	186,638	(25.15)
2009 - 2013	38,646	(26.56)	197,205	(26.57)
2014 - 2019	39,989	(27.48)	203,137	(27.37)
**Ethnicity**				
White	96,994	(66.66)	495,376	(66.74)
South Asian	3,256	(2.24)	20,810	(2.80)
Black	2,230	(1.53)	13,968	(1.88)
Other	1,457	(1.00)	8,977	(1.21)
Unknown	41,578	(28.57)	203,090	(27.36)
**Body mass index**				
Underweight (<18.5)	2,564	(1.76)	15,471	(2.08)
Normal weight (18.5-24.9)	53,447	(36.73)	271,405	(36.57)
Overweight (25.0-29.9)	45,434	(31.22)	221,370	(29.83)
Obese (≥30.0)	35,985	(24.73)	176,316	(23.76)
Unknown	8,085	(5.56)	57,659	(7.77)
**Alcohol consumption**				
Non-drinker	17,206	(11.82)	94,142	(12.68)
Current drinker	76,940	(52.87)	376,380	(50.71)
Ex-drinker	6,286	(4.32)	29,950	(4.04)
Unknown	45,083	(30.98)	241,749	(32.57)

**Problem drinker**				
	3,039	(2.09)	14,917	(2.01)
**Smoking status**				
Non-smoker	57,421	(39.46)	291,054	(39.21)
Current smoker	19,290	(13.26)	129,304	(17.42)
Ex-smoker	66,888	(45.97)	309,085	(41.64)
Unknown	1,916	(1.32)	12,778	(1.72)
**Prescribed**				
Oral corticosteroids	25,778	(17.72)	127,424	(17.17)
Hormone replacement therapy	50,899	(34.98)	238,416	(32.12)
Bisphosphonate therapy	6,061	(4.17)	26,900	(3.62)
**History of**				
Fracture §	29,481	(20.26)	150,532	(20.28)
Osteoporosis	12,491	(8.58)	48,888	(6.59)
Eating disorder	815	(0.56)	4,626	(0.62)
**Comorbidities**				
Coeliac disease	543	(0.37)	3,553	(0.48)
Inflammatory bowel disease	6,028	(4.14)	25,602	(3.45)
Systemic lupus erythematosus	438	(0.30)	2,399	(0.32)
Rheumatoid arthritis	2,992	(2.06)	16,234	(2.19)
Chronic kidney disease	26,257	(18.04)	128,172	(17.27)
Liver disease	1,755	(1.21)	6,345	(0.85)
Epilepsy	2,286	(1.57)	12,404	(1.67)

SD = standard deviation; IQR = interquartile range.
* Data are n (%) unless otherwise stated. The characteristics of the study participants were measured prior to or near the index date†. When information on body mass index, height and weight, alcohol consumption, and smoking status was unavailable prior to the index date, we used information recorded at any point in the clinical record. We ran a sensitivity analysis using only pre-index data.
† For cancer survivors, the index date was the date of their cancer diagnosis. In the matched cancer-free cohort, the matched cancer survivor's index date was allocated to each individual. Cancer-free people were individually matched on year of birth (+/- 3 years), sex and general practice to participants in the cancer survivor cohort.
‡ Index date to end of follow-up.
§ In adulthood.

Table S12: Characteristics of cancer survivors and matched controls from the general population (cervical cancer; ICD-10 C53).*

	Cancer survivors		Cancer-free controls	
**All participants**	6,267		35,698	
**Time from index date† to end of follow-up (years)**				
Mean (SD)	6.49 (5.11)		7.63 (5.26)	
Median (IQR)	4.79 (2.34- 9.36)		6.31 (3.19-11.16)	
Range	1.00-21.02		1.00-21.07	
**Total person-years ‡**				
	40699.33		272450.2	
**Age (years)**				
Mean (SD)	46.74 (16.09)		48.37 (16.66)	
Median (IQR)	43 (34-57)		45 (35-60)	
**Age group at cancer diagnosis (years)**				
18 - 59	4,908	(78.31)	26,521	(74.29)
60 - 79	1,074	(17.14)	7,249	(20.31)
≥80	285	(4.55)	1,928	(5.40)
**Sex**				
Women	6,267	(100.00)	35,698	(100.00)
**Patient-level Index of Multiple Deprivation (practice postcode-based)**				
1 (least deprived)	1,104	(17.62)	6,816	(19.09)
2	1,136	(18.13)	7,082	(19.84)
3	1,175	(18.75)	6,866	(19.23)
4	1,348	(21.51)	7,339	(20.56)
5 (most deprived)	1,499	(23.92)	7,572	(21.21)
**Calendar year of cancer diagnosis**				
1999 - 2003	1,467	(23.41)	8,295	(23.24)
2004 - 2008	1,562	(24.92)	8,991	(25.19)
2009 - 2013	1,579	(25.20)	9,136	(25.59)
2014 - 2019	1,659	(26.47)	9,276	(25.98)
**Ethnicity**				
White	3,946	(62.96)	22,970	(64.35)
South Asian	98	(1.56)	1,280	(3.59)
Black	83	(1.32)	919	(2.57)
Other	94	(1.50)	588	(1.65)
Unknown	2,046	(32.65)	9,941	(27.85)
**Body mass index**				
Underweight (<18.5)	198	(3.16)	844	(2.36)
Normal weight (18.5-24.9)	2,462	(39.29)	14,164	(39.68)
Overweight (25.0-29.9)	1,585	(25.29)	9,508	(26.63)
Obese (≥30.0)	1,450	(23.14)	8,104	(22.70)
Unknown	572	(9.13)	3,078	(8.62)
**Alcohol consumption**				
Non-drinker	693	(11.06)	4,395	(12.31)
Current drinker	2,847	(45.43)	16,816	(47.11)
Ex-drinker	214	(3.41)	1,186	(3.32)
Unknown	2,513	(40.10)	13,301	(37.26)

**Problem drinker**				
	205	(3.27)	858	(2.40)
**Smoking status**				
Non-smoker	1,969	(31.42)	13,590	(38.07)
Current smoker	1,349	(21.53)	7,236	(20.27)
Ex-smoker	2,799	(44.66)	14,171	(39.70)
Unknown	150	(2.39)	701	(1.96)
**Prescribed**				
Oral corticosteroids	918	(14.65)	4,815	(13.49)
Hormone replacement therapy	2,403	(38.34)	9,680	(27.12)
Bisphosphonate therapy	85	(1.36)	631	(1.77)
**History of**				
Fracture §	1,179	(18.81)	6,067	(17.00)
Osteoporosis	176	(2.81)	1,199	(3.36)
Eating disorder	77	(1.23)	380	(1.06)
**Comorbidities**				
Coeliac disease	17	(0.27)	155	(0.43)
Inflammatory bowel disease	356	(5.68)	1,029	(2.88)
Systemic lupus erythematosus	19	(0.30)	98	(0.27)
Rheumatoid arthritis	92	(1.47)	496	(1.39)
Chronic kidney disease	627	(10.00)	2,908	(8.15)
Liver disease	75	(1.20)	194	(0.54)
Epilepsy	100	(1.60)	559	(1.57)

SD = standard deviation; IQR = interquartile range.
* Data are n (%) unless otherwise stated. The characteristics of the study participants were measured prior to or near the index date†. When information on body mass index, height and weight, alcohol consumption, and smoking status was unavailable prior to the index date, we used information recorded at any point in the clinical record. We ran a sensitivity analysis using only pre-index data.
† For cancer survivors, the index date was the date of their cancer diagnosis. In the matched cancer-free cohort, the matched cancer survivor's index date was allocated to each individual. Cancer-free people were individually matched on year of birth (+/- 3 years), sex and general practice to participants in the cancer survivor cohort.
‡ Index date to end of follow-up.
§ In adulthood.

Table S13: Characteristics of cancer survivors and matched controls from the general population (uterine cancer; ICD-10 C54-55).*

	Cancer survivors		Cancer-free controls	
**All participants**	13,970		79,585	
**Time from index date† to end of follow-up (years)**				
Mean (SD)	6.29 (4.60)		6.97 (4.72)	
Median (IQR)	4.95 (2.51- 9.00)		5.88 (3.08- 9.89)	
Range	1.00-21.07		1.00-21.08	
**Total person-years ‡**				
	87814.83		554681	
**Age (years)**				
Mean (SD)	65.64 (11.19)		65.64 (11.28)	
Median (IQR)	66 (58-73)		66 (58-73)	
**Age group at cancer diagnosis (years)**				
18 - 59	4,129	(29.56)	23,712	(29.79)
60 - 79	8,262	(59.14)	46,748	(58.74)
≥80	1,579	(11.30)	9,125	(11.47)
**Sex**				
Women	13,970	(100.00)	79,585	(100.00)
**Patient-level Index of Multiple Deprivation (practice postcode-based)**				
1 (least deprived)	3,110	(22.26)	18,415	(23.14)
2	3,314	(23.72)	18,055	(22.69)
3	2,892	(20.70)	16,419	(20.63)
4	2,635	(18.86)	14,835	(18.64)
5 (most deprived)	2,004	(14.35)	11,792	(14.82)
**Calendar year of cancer diagnosis**				
1999 - 2003	2,503	(17.92)	14,038	(17.64)
2004 - 2008	3,326	(23.81)	18,938	(23.80)
2009 - 2013	3,935	(28.17)	22,558	(28.34)
2014 - 2019	4,206	(30.11)	24,051	(30.22)
**Ethnicity**				
White	9,345	(66.89)	54,059	(67.93)
South Asian	355	(2.54)	2,271	(2.85)
Black	233	(1.67)	1,469	(1.85)
Other	117	(0.84)	825	(1.04)
Unknown	3,920	(28.06)	20,961	(26.34)
**Body mass index**				
Underweight (<18.5)	117	(0.84)	1,479	(1.86)
Normal weight (18.5-24.9)	2,770	(19.83)	26,770	(33.64)
Overweight (25.0-29.9)	3,633	(26.01)	24,592	(30.90)
Obese (≥30.0)	6,798	(48.66)	19,856	(24.95)
Unknown	652	(4.67)	6,888	(8.65)
**Alcohol consumption**				
Non-drinker	2,034	(14.56)	10,329	(12.98)
Current drinker	7,473	(53.49)	40,770	(51.23)
Ex-drinker	810	(5.80)	3,617	(4.54)
Unknown	3,653	(26.15)	24,869	(31.25)

**Problem drinker**				
	198	(1.42)	1,553	(1.95)
**Smoking status**				
Non-smoker	6,086	(43.56)	31,084	(39.06)
Current smoker	1,345	(9.63)	13,327	(16.75)
Ex-smoker	6,360	(45.53)	34,029	(42.76)
Unknown	179	(1.28)	1,145	(1.44)
**Prescribed**				
Oral corticosteroids	2,402	(17.19)	15,411	(19.36)
Hormone replacement therapy	5,232	(37.45)	26,718	(33.57)
Bisphosphonate therapy	340	(2.43)	3,535	(4.44)
**History of**				
Fracture §	2,643	(18.92)	17,246	(21.67)
Osteoporosis	710	(5.08)	6,278	(7.89)
Eating disorder	60	(0.43)	428	(0.54)
**Comorbidities**				
Coeliac disease	31	(0.22)	412	(0.52)
Inflammatory bowel disease	586	(4.19)	2,940	(3.69)
Systemic lupus erythematosus	35	(0.25)	254	(0.32)
Rheumatoid arthritis	309	(2.21)	1,956	(2.46)
Chronic kidney disease	3,537	(25.32)	16,606	(20.87)
Liver disease	231	(1.65)	778	(0.98)
Epilepsy	223	(1.60)	1,311	(1.65)

SD = standard deviation; IQR = interquartile range.

* Data are n (%) unless otherwise stated. The characteristics of the study participants were measured prior to or near the index date†. When information on body mass index, height and weight, alcohol consumption, and smoking status was unavailable prior to the index date, we used information recorded at any point in the clinical record. We ran a sensitivity analysis using only pre-index data.

† For cancer survivors, the index date was the date of their cancer diagnosis. In the matched cancer-free cohort, the matched cancer survivor's index date was allocated to each individual. Cancer-free people were individually matched on year of birth (+/- 3 years), sex and general practice to participants in the cancer survivor cohort.

‡ Index date to end of follow-up.

§ In adulthood.

Table S14: Characteristics of cancer survivors and matched controls from the general population (ovarian cancer; ICD-10 C56).*

	Cancer survivors		Cancer-free controls	
**All participants**	12,475		85,766	
**Time from index date† to end of follow-up (years)**				
Mean (SD)	5.59 (4.58)		7.48 (5.00)	
Median (IQR)	3.96 (2.08- 7.78)		6.35 (3.30-10.71)	
Range	1.00-21.07		1.00-21.08	
**Total person-years ‡**				
	69760.25		641612.8	
**Age (years)**				
Mean (SD)	59.63 (14.74)		61.29 (14.28)	
Median (IQR)	61 (50-70)		62 (52-72)	
**Age group at cancer diagnosis (years)**				
18 - 59	5,887	(47.19)	36,294	(42.32)
60 - 79	5,566	(44.62)	41,488	(48.37)
≥80	1,022	(8.19)	7,984	(9.31)
**Sex**				
Women	12,475	(100.00)	85,766	(100.00)
**Patient-level Index of Multiple Deprivation (practice postcode-based)**				
1 (least deprived)	3,014	(24.16)	20,005	(23.33)
2	2,819	(22.60)	19,158	(22.34)
3	2,520	(20.20)	17,160	(20.01)
4	2,273	(18.22)	16,010	(18.67)
5 (most deprived)	1,835	(14.71)	13,360	(15.58)
**Calendar year of cancer diagnosis**				
1999 - 2003	3,001	(24.06)	19,499	(22.74)
2004 - 2008	3,191	(25.58)	21,408	(24.96)
2009 - 2013	3,293	(26.40)	22,949	(26.76)
2014 - 2019	2,990	(23.97)	21,910	(25.55)
**Ethnicity**				
White	7,298	(58.50)	57,008	(66.47)
South Asian	312	(2.50)	2,607	(3.04)
Black	156	(1.25)	1,816	(2.12)
Other	113	(0.91)	1,033	(1.20)
Unknown	4,596	(36.84)	23,302	(27.17)
**Body mass index**				
Underweight (<18.5)	268	(2.15)	1,787	(2.08)
Normal weight (18.5-24.9)	4,588	(36.78)	30,561	(35.63)
Overweight (25.0-29.9)	3,732	(29.92)	25,408	(29.62)
Obese (≥30.0)	3,003	(24.07)	20,507	(23.91)
Unknown	884	(7.09)	7,503	(8.75)
**Alcohol consumption**				
Non-drinker	1,478	(11.85)	10,759	(12.54)
Current drinker	6,133	(49.16)	43,227	(50.40)
Ex-drinker	517	(4.14)	3,401	(3.97)
Unknown	4,347	(34.85)	28,379	(33.09)

**Problem drinker**				
	226	(1.81)	1,755	(2.05)
**Smoking status**				
Non-smoker	5,009	(40.15)	32,980	(38.45)
Current smoker	1,851	(14.84)	15,341	(17.89)
Ex-smoker	5,349	(42.88)	36,049	(42.03)
Unknown	266	(2.13)	1,396	(1.63)
**Prescribed**				
Oral corticosteroids	2,288	(18.34)	14,680	(17.12)
Hormone replacement therapy	4,802	(38.49)	27,435	(31.99)
Bisphosphonate therapy	372	(2.98)	3,156	(3.68)
**History of**				
Fracture §	2,394	(19.19)	16,884	(19.69)
Osteoporosis	685	(5.49)	5,635	(6.57)
Eating disorder	76	(0.61)	520	(0.61)
**Comorbidities**				
Coeliac disease	56	(0.45)	412	(0.48)
Inflammatory bowel disease	621	(4.98)	2,843	(3.31)
Systemic lupus erythematosus	33	(0.26)	256	(0.30)
Rheumatoid arthritis	280	(2.24)	1,934	(2.25)
Chronic kidney disease	2,300	(18.44)	14,475	(16.88)
Liver disease	284	(2.28)	751	(0.88)
Epilepsy	222	(1.78)	1,344	(1.57)

SD = standard deviation; IQR = interquartile range.
* Data are n (%) unless otherwise stated. The characteristics of the study participants were measured prior to or near the index date†. When information on body mass index, height and weight, alcohol consumption, and smoking status was unavailable prior to the index date, we used information recorded at any point in the clinical record. We ran a sensitivity analysis using only pre-index data.
† For cancer survivors, the index date was the date of their cancer diagnosis. In the matched cancer-free cohort, the matched cancer survivor's index date was allocated to each individual. Cancer-free people were individually matched on year of birth (+/- 3 years), sex and general practice to participants in the cancer survivor cohort.
‡ Index date to end of follow-up.
§ In adulthood.

Table S15: Characteristics of cancer survivors and matched controls from the general population (prostate cancer; ICD-10 C61).*

	Cancer survivors		Cancer-free controls	
**All participants**	117,875		612,935	
**Time from index date† to end of follow-up (years)**				
Mean (SD)	5.80 (4.14)		6.49 (4.41)	
Median (IQR)	4.65 (2.47- 8.10)		5.41 (2.89- 9.15)	
Range	1.00-21.06		1.00-21.08	
**Total person-years ‡**				
	683388.1		3977060	
**Age (years)**				
Mean (SD)	71.04 (9.19)		71.08 (9.15)	
Median (IQR)	71 (65-78)		71 (65-78)	
**Age group at cancer diagnosis (years)**				
18 - 59	13,019	(11.04)	66,809	(10.90)
60 - 79	83,225	(70.60)	432,819	(70.61)
≥80	21,631	(18.35)	113,307	(18.49)
**Sex**				
Men	117,875	(100.00)	612,935	(100.00)
**Patient-level Index of Multiple Deprivation (practice postcode-based)**				
1 (least deprived)	31,802	(26.98)	156,537	(25.54)
2	28,619	(24.28)	144,600	(23.59)
3	23,168	(19.65)	122,561	(20.00)
4	19,244	(16.33)	104,939	(17.12)
5 (most deprived)	14,952	(12.68)	83,808	(13.67)
**Calendar year of cancer diagnosis**				
1999 - 2003	21,470	(18.21)	112,662	(18.38)
2004 - 2008	28,450	(24.14)	148,804	(24.28)
2009 - 2013	32,056	(27.19)	166,440	(27.15)
2014 - 2019	35,899	(30.46)	185,029	(30.19)
**Ethnicity**				
White	77,197	(65.49)	398,850	(65.07)
South Asian	1,596	(1.35)	14,557	(2.37)
Black	3,897	(3.31)	9,997	(1.63)
Other	879	(0.75)	4,923	(0.80)
Unknown	34,306	(29.10)	184,608	(30.12)
**Body mass index**				
Underweight (<18.5)	906	(0.77)	5,588	(0.91)
Normal weight (18.5-24.9)	35,391	(30.02)	172,666	(28.17)
Overweight (25.0-29.9)	52,385	(44.44)	244,604	(39.91)
Obese (≥30.0)	23,664	(20.08)	124,434	(20.30)
Unknown	5,529	(4.69)	65,643	(10.71)
**Alcohol consumption**				
Non-drinker	7,663	(6.50)	41,444	(6.76)
Current drinker	73,766	(62.58)	355,780	(58.05)
Ex-drinker	4,511	(3.83)	21,764	(3.55)
Unknown	31,935	(27.09)	193,947	(31.64)

**Problem drinker**				
	3,738	(3.17)	21,449	(3.50)
**Smoking status**				
Non-smoker	32,977	(27.98)	159,313	(25.99)
Current smoker	14,020	(11.89)	110,334	(18.00)
Ex-smoker	69,513	(58.97)	325,592	(53.12)
Unknown	1,365	(1.16)	17,696	(2.89)
**Prescribed**				
Oral corticosteroids	21,897	(18.58)	99,192	(16.18)
Hormone replacement therapy	694	(0.59)	931	(0.15)
Bisphosphonate therapy	1,596	(1.35)	7,033	(1.15)
**History of**				
Fracture §	24,346	(20.65)	121,568	(19.83)
Osteoporosis	2,459	(2.09)	11,122	(1.81)
Eating disorder	165	(0.14)	778	(0.13)
**Comorbidities**				
Coeliac disease	352	(0.30)	1,908	(0.31)
Inflammatory bowel disease	4,090	(3.47)	18,970	(3.09)
Systemic lupus erythematosus	95	(0.08)	577	(0.09)
Rheumatoid arthritis	1,954	(1.66)	9,137	(1.49)
Chronic kidney disease	31,881	(27.05)	140,529	(22.93)
Liver disease	1,399	(1.19)	6,727	(1.10)
Epilepsy	1,964	(1.67)	11,250	(1.84)

SD = standard deviation; IQR = interquartile range.
* Data are n (%) unless otherwise stated. The characteristics of the study participants were measured prior to or near the index date†. When information on body mass index, height and weight, alcohol consumption, and smoking status was unavailable prior to the index date, we used information recorded at any point in the clinical record. We ran a sensitivity analysis using only pre-index data.
† For cancer survivors, the index date was the date of their cancer diagnosis. In the matched cancer-free cohort, the matched cancer survivor's index date was allocated to each individual. Cancer-free people were individually matched on year of birth (+/- 3 years), sex and general practice to participants in the cancer survivor cohort.
‡ Index date to end of follow-up.
§ In adulthood.

Table S16: Characteristics of cancer survivors and matched controls from the general population (kidney cancer; ICD-10 C64).*

	Cancer survivors		Cancer-free controls	
**All participants**	10,819		64,193	
**Time from index date† to end of follow-up (years)**				
Mean (SD)	5.20 (4.01)		6.50 (4.57)	
Median (IQR)	3.92 (2.16- 7.02)		5.27 (2.88- 8.98)	
Range	1.00-21.06		1.00-21.07	
**Total person-years ‡**				
	56241.835		417174.82	
**Age (years)**				
Mean (SD)	65.29 (13.18)		65.81 (13.06)	
Median (IQR)	66 (56-75)		67 (57-75)	
**Age group at cancer diagnosis (years)**				
18 - 59	3,473	(32.10)	19,632	(30.58)
60 - 79	5,779	(53.42)	34,772	(54.17)
≥80	1,567	(14.48)	9,789	(15.25)
**Sex**				
Women	4,160	(38.45)	24,533	(38.22)
Men	6,659	(61.55)	39,660	(61.78)
**Patient-level Index of Multiple Deprivation (practice postcode-based)**				
1 (least deprived)	2,354	(21.76)	14,547	(22.66)
2	2,363	(21.84)	14,216	(22.15)
3	2,155	(19.92)	12,952	(20.18)
4	2,055	(18.99)	11,860	(18.48)
5 (most deprived)	1,885	(17.42)	10,563	(16.46)
**Calendar year of cancer diagnosis**				
1999 - 2003	1,747	(16.15)	10,736	(16.72)
2004 - 2008	2,120	(19.60)	13,082	(20.38)
2009 - 2013	2,990	(27.64)	17,779	(27.70)
2014 - 2019	3,962	(36.62)	22,596	(35.20)
**Ethnicity**				
White	7,175	(66.32)	42,803	(66.68)
South Asian	279	(2.58)	2,032	(3.17)
Black	229	(2.12)	1,311	(2.04)
Other	116	(1.07)	743	(1.16)
Unknown	3,020	(27.91)	17,304	(26.96)
**Body mass index**				
Underweight (<18.5)	172	(1.59)	898	(1.40)
Normal weight (18.5-24.9)	2,970	(27.45)	19,344	(30.13)
Overweight (25.0-29.9)	3,950	(36.51)	23,140	(36.05)
Obese (≥30.0)	3,217	(29.73)	14,257	(22.21)
Unknown	510	(4.71)	6,554	(10.21)
**Alcohol consumption**				
Non-drinker	1,062	(9.82)	5,623	(8.76)
Current drinker	6,202	(57.33)	35,485	(55.28)
Ex-drinker	541	(5.00)	2,362	(3.68)

Unknown	3,014	(27.86)	20,723	(32.28)
**Problem drinker**				
	324	(2.99)	2,115	(3.29)
**Smoking status**				
Non-smoker	2,953	(27.29)	20,167	(31.42)
Current smoker	1,578	(14.59)	11,951	(18.62)
Ex-smoker	6,146	(56.81)	30,521	(47.55)
Unknown	142	(1.31)	1,554	(2.42)
**Prescribed**				
Oral corticosteroids	2,669	(24.67)	10,681	(16.64)
Hormone replacement therapy	1,545	(14.28)	7,859	(12.24)
Bisphosphonate therapy	299	(2.76)	1,581	(2.46)
**History of**				
Fracture §	2,603	(24.06)	14,383	(22.41)
Osteoporosis	546	(5.05)	2,933	(4.57)
Eating disorder	43	(0.40)	178	(0.28)
**Comorbidities**				
Coeliac disease	53	(0.49)	228	(0.36)
Inflammatory bowel disease	683	(6.31)	2,150	(3.35)
Systemic lupus erythematosus	28	(0.26)	116	(0.18)
Rheumatoid arthritis	307	(2.84)	1,213	(1.89)
Chronic kidney disease	5,510	(50.93)	13,106	(20.42)
Liver disease	402	(3.72)	720	(1.12)
Epilepsy	245	(2.26)	1,113	(1.73)

SD = standard deviation; IQR = interquartile range.
* Data are n (%) unless otherwise stated. The characteristics of the study participants were measured prior to or near the index date†. When information on body mass index, height and weight, alcohol consumption, and smoking status was unavailable prior to the index date, we used information recorded at any point in the clinical record. We ran a sensitivity analysis using only pre-index data.
† For cancer survivors, the index date was the date of their cancer diagnosis. In the matched cancer-free cohort, the matched cancer survivor's index date was allocated to each individual. Cancer-free people were individually matched on year of birth (+/- 3 years), sex and general practice to participants in the cancer survivor cohort.
‡ Index date to end of follow-up.
§ In adulthood.

Table S17: Characteristics of cancer survivors and matched controls from the general population (bladder cancer; ICD-10 C67).*

	Cancer survivors		Cancer-free controls	
**All participants**	36,820		204,026	
**Time from index date† to end of follow-up (years)**				
Mean (SD)	5.89 (4.34)		6.74 (4.58)	
Median (IQR)	4.65 (2.39- 8.31)		5.60 (3.01- 9.48)	
Range	1.00-21.07		1.00-21.08	
**Total person-years ‡**	216956.1		1374139	
**Age (years)**				
Mean (SD)	70.88 (11.67)		71.06 (11.51)	
Median (IQR)	72 (64-79)		72 (64-79)	
**Age group at cancer diagnosis (years)**				
18 - 59	5,853	(15.90)	31,163	(15.27)
60 - 79	22,127	(60.10)	123,280	(60.42)
≥80	8,840	(24.01)	49,583	(24.30)
**Sex**				
Women	9,266	(25.17)	50,594	(24.80)
Men	27,554	(74.83)	153,432	(75.20)
**Patient-level Index of Multiple Deprivation (practice postcode-based)**				
1 (least deprived)	8,300	(22.54)	47,027	(23.05)
2	8,506	(23.10)	46,775	(22.93)
3	7,453	(20.24)	41,830	(20.50)
4	6,826	(18.54)	37,367	(18.31)
5 (most deprived)	5,707	(15.50)	30,867	(15.13)
**Calendar year of cancer diagnosis**				
1999 - 2003	8,318	(22.59)	45,353	(22.23)
2004 - 2008	9,395	(25.52)	51,806	(25.39)
2009 - 2013	9,558	(25.96)	53,403	(26.17)
2014 - 2019	9,549	(25.93)	53,464	(26.20)
**Ethnicity**				
White	23,753	(64.51)	129,193	(63.32)
South Asian	479	(1.30)	4,068	(1.99)
Black	201	(0.55)	2,292	(1.12)
Other	199	(0.54)	1,401	(0.69)
Unknown	12,188	(33.10)	67,072	(32.87)
**Body mass index**				
Underweight (<18.5)	658	(1.79)	2,789	(1.37)
Normal weight (18.5-24.9)	11,471	(31.15)	60,387	(29.60)
Overweight (25.0-29.9)	14,138	(38.40)	74,418	(36.47)
Obese (≥30.0)	8,310	(22.57)	40,378	(19.79)
Unknown	2,243	(6.09)	26,054	(12.77)
**Alcohol consumption**				
Non-drinker	3,206	(8.71)	17,516	(8.59)
Current drinker	21,956	(59.63)	111,448	(54.62)
Ex-drinker	1,743	(4.73)	7,713	(3.78)

Unknown	9,915	(26.93)	67,349	(33.01)
**Problem drinker**				
	1,135	(3.08)	5,844	(2.86)
**Smoking status**				
Non-smoker	7,754	(21.06)	59,606	(29.21)
Current smoker	6,505	(17.67)	35,922	(17.61)
Ex-smoker	21,932	(59.57)	102,157	(50.07)
Unknown	629	(1.71)	6,341	(3.11)
**Prescribed**				
Oral corticosteroids	7,649	(20.77)	34,908	(17.11)
Hormone replacement therapy	2,899	(7.87)	13,320	(6.53)
Bisphosphonate therapy	1,083	(2.94)	5,194	(2.55)
**History of**				
Fracture §	8,006	(21.74)	43,121	(21.14)
Osteoporosis	1,531	(4.16)	8,096	(3.97)
Eating disorder	84	(0.23)	454	(0.22)
**Comorbidities**				
Coeliac disease	134	(0.36)	670	(0.33)
Inflammatory bowel disease	1,682	(4.57)	6,619	(3.24)
Systemic lupus erythematosus	70	(0.19)	278	(0.14)
Rheumatoid arthritis	805	(2.19)	3,757	(1.84)
Chronic kidney disease	12,112	(32.90)	51,685	(25.33)
Liver disease	637	(1.73)	2,040	(1.00)
Epilepsy	658	(1.79)	3,617	(1.77)

SD = standard deviation; IQR = interquartile range.
* Data are n (%) unless otherwise stated. The characteristics of the study participants were measured prior to or near the index date†. When information on body mass index, height and weight, alcohol consumption, and smoking status was unavailable prior to the index date, we used information recorded at any point in the clinical record. We ran a sensitivity analysis using only pre-index data.
† For cancer survivors, the index date was the date of their cancer diagnosis. In the matched cancer-free cohort, the matched cancer survivor's index date was allocated to each individual. Cancer-free people were individually matched on year of birth (+/- 3 years), sex and general practice to participants in the cancer survivor cohort.
‡ Index date to end of follow-up.
§ In adulthood.

Table S18: Characteristics of cancer survivors and matched controls from the general population (central nervous system cancer; ICD-10 C71-72).*

	Cancer survivors		Cancer-free controls	
**All participants**	6,236		38,949	
**Time from index date† to end of follow-up (years)**				
Mean (SD)	4.42 (4.04)		7.14 (4.90)	
Median (IQR)	2.74 (1.52- 5.87)		5.96 (3.08-10.19)	
Range	1.00-21.07		1.00-21.07	
**Total person-years ‡**				
	27550.77		278037.8	
**Age (years)**				
Mean (SD)	51.06 (15.47)		53.89 (16.19)	
Median (IQR)	52 (39-62)		55 (42-66)	
**Age group at cancer diagnosis (years)**				
18 - 59	4,236	(67.93)	23,462	(60.24)
60 - 79	1,833	(29.39)	13,558	(34.81)
≥80	167	(2.68)	1,929	(4.95)
**Sex**				
Women	2,708	(43.43)	16,998	(43.64)
Men	3,528	(56.57)	21,951	(56.36)
**Patient-level Index of Multiple Deprivation (practice postcode-based)**				
1 (least deprived)	1,473	(23.62)	9,018	(23.15)
2	1,360	(21.81)	8,627	(22.15)
3	1,213	(19.45)	7,673	(19.70)
4	1,163	(18.65)	7,327	(18.81)
5 (most deprived)	1,022	(16.39)	6,265	(16.09)
**Calendar year of cancer diagnosis**				
1999 - 2003	1,177	(18.87)	7,694	(19.75)
2004 - 2008	1,451	(23.27)	9,739	(25.00)
2009 - 2013	1,705	(27.34)	10,717	(27.52)
2014 - 2019	1,903	(30.52)	10,799	(27.73)
**Ethnicity**				
White	3,418	(54.81)	23,483	(60.29)
South Asian	206	(3.30)	1,365	(3.50)
Black	90	(1.44)	781	(2.01)
Other	61	(0.98)	547	(1.40)
Unknown	2,461	(39.46)	12,773	(32.79)
**Body mass index**				
Underweight (<18.5)	91	(1.46)	542	(1.39)
Normal weight (18.5-24.9)	2,054	(32.94)	12,101	(31.07)
Overweight (25.0-29.9)	2,066	(33.13)	12,236	(31.42)
Obese (≥30.0)	1,291	(20.70)	7,996	(20.53)
Unknown	734	(11.77)	6,074	(15.59)
**Alcohol consumption**				
Non-drinker	598	(9.59)	3,475	(8.92)
Current drinker	2,866	(45.96)	19,478	(50.01)
Ex-drinker	251	(4.03)	1,193	(3.06)

Unknown	2,521	(40.43)	14,803	(38.01)
**Problem drinker**				
	174	(2.79)	1,327	(3.41)
**Smoking status**				
Non-smoker	2,345	(37.60)	14,241	(36.56)
Current smoker	978	(15.68)	7,569	(19.43)
Ex-smoker	2,711	(43.47)	15,728	(40.38)
Unknown	202	(3.24)	1,411	(3.62)
**Prescribed**				
Oral corticosteroids	3,369	(54.03)	4,879	(12.53)
Hormone replacement therapy	901	(14.45)	4,696	(12.06)
Bisphosphonate therapy	90	(1.44)	622	(1.60)
**History of**				
Fracture §	1,396	(22.39)	7,932	(20.37)
Osteoporosis	159	(2.55)	995	(2.55)
Eating disorder	33	(0.53)	174	(0.45)
**Comorbidities**				
Coeliac disease	22	(0.35)	121	(0.31)
Inflammatory bowel disease	240	(3.85)	905	(2.32)
Systemic lupus erythematosus	6	(0.10)	59	(0.15)
Rheumatoid arthritis	81	(1.30)	432	(1.11)
Chronic kidney disease	498	(7.99)	3,303	(8.48)
Liver disease	123	(1.97)	287	(0.74)
Epilepsy	1,729	(27.73)	660	(1.69)

SD = standard deviation; IQR = interquartile range.
* Data are n (%) unless otherwise stated. The characteristics of the study participants were measured prior to or near the index date†. When information on body mass index, height and weight, alcohol consumption, and smoking status was unavailable prior to the index date, we used information recorded at any point in the clinical record. We ran a sensitivity analysis using only pre-index data.
† For cancer survivors, the index date was the date of their cancer diagnosis. In the matched cancer-free cohort, the matched cancer survivor's index date was allocated to each individual. Cancer-free people were individually matched on year of birth (+/- 3 years), sex and general practice to participants in the cancer survivor cohort.
‡ Index date to end of follow-up.
§ In adulthood.

Table S19: Characteristics of cancer survivors and matched controls from the general population (thyroid cancer; ICD-10 C73).*

	Cancer survivors		Cancer-free controls	
**All participants**	5,867		30,776	
**Time from index date† to end of follow-up (years)**				
Mean (SD)	6.46 (4.62)		6.59 (4.68)	
Median (IQR)	5.16 (2.77- 9.04)		5.31 (2.82- 9.28)	
Range	1.00-21.03		1.00-21.05	
**Total person-years ‡**				
	37926.02		202888.1	
**Age (years)**				
Mean (SD)	50.62 (16.00)		51.12 (16.15)	
Median (IQR)	49 (38-62)		50 (39-63)	
**Age group at cancer diagnosis (years)**				
18 - 59	4,153	(70.79)	21,363	(69.41)
60 - 79	1,447	(24.66)	7,904	(25.68)
≥80	267	(4.55)	1,509	(4.90)
**Sex**				
Women	4,442	(75.71)	23,162	(75.26)
Men	1,425	(24.29)	7,614	(24.74)
**Patient-level Index of Multiple Deprivation (practice postcode-based)**				
1 (least deprived)	1,342	(22.87)	6,836	(22.21)
2	1,217	(20.74)	6,386	(20.75)
3	1,189	(20.27)	5,866	(19.06)
4	1,122	(19.12)	6,110	(19.85)
5 (most deprived)	993	(16.93)	5,558	(18.06)
**Calendar year of cancer diagnosis**				
1999 - 2003	812	(13.84)	4,397	(14.29)
2004 - 2008	1,179	(20.10)	6,163	(20.03)
2009 - 2013	1,679	(28.62)	8,780	(28.53)
2014 - 2019	2,197	(37.45)	11,436	(37.16)
**Ethnicity**				
White	3,832	(65.31)	19,709	(64.04)
South Asian	427	(7.28)	1,698	(5.52)
Black	158	(2.69)	930	(3.02)
Other	196	(3.34)	668	(2.17)
Unknown	1,254	(21.37)	7,771	(25.25)
**Body mass index**				
Underweight (<18.5)	106	(1.81)	603	(1.96)
Normal weight (18.5-24.9)	2,136	(36.41)	11,323	(36.79)
Overweight (25.0-29.9)	1,826	(31.12)	9,089	(29.53)
Obese (≥30.0)	1,492	(25.43)	6,848	(22.25)
Unknown	307	(5.23)	2,913	(9.47)
**Alcohol consumption**				
Non-drinker	771	(13.14)	3,324	(10.80)
Current drinker	2,853	(48.63)	14,585	(47.39)
Ex-drinker	224	(3.82)	988	(3.21)

Unknown	2,019	(34.41)	11,879	(38.60)
**Problem drinker**				
	141	(2.40)	958	(3.11)
**Smoking status**				
Non-smoker	2,401	(40.92)	11,954	(38.84)
Current smoker	666	(11.35)	5,879	(19.10)
Ex-smoker	2,738	(46.67)	12,245	(39.79)
Unknown	62	(1.06)	698	(2.27)
**Prescribed**				
Oral corticosteroids	1,003	(17.10)	4,285	(13.92)
Hormone replacement therapy	1,542	(26.28)	7,037	(22.87)
Bisphosphonate therapy	108	(1.84)	481	(1.56)
**History of**				
Fracture §	1,178	(20.08)	5,800	(18.85)
Osteoporosis	244	(4.16)	1,017	(3.30)
Eating disorder	37	(0.63)	263	(0.85)
**Comorbidities**				
Coeliac disease	20	(0.34)	114	(0.37)
Inflammatory bowel disease	252	(4.30)	927	(3.01)
Systemic lupus erythematosus	22	(0.37)	77	(0.25)
Rheumatoid arthritis	108	(1.84)	419	(1.36)
Chronic kidney disease	633	(10.79)	2,728	(8.86)
Liver disease	78	(1.33)	240	(0.78)
Epilepsy	106	(1.81)	527	(1.71)

SD = standard deviation; IQR = interquartile range.
* Data are n (%) unless otherwise stated. The characteristics of the study participants were measured prior to or near the index date†. When information on body mass index, height and weight, alcohol consumption, and smoking status was unavailable prior to the index date, we used information recorded at any point in the clinical record. We ran a sensitivity analysis using only pre-index data.
† For cancer survivors, the index date was the date of their cancer diagnosis. In the matched cancer-free cohort, the matched cancer survivor's index date was allocated to each individual. Cancer-free people were individually matched on year of birth (+/- 3 years), sex and general practice to participants in the cancer survivor cohort.
‡ Index date to end of follow-up.
§ In adulthood.

Table S20: Characteristics of cancer survivors and matched controls from the general population (non-Hodgkin lymphoma; ICD-10 C82-85).*

	Cancer survivors		Cancer-free controls	
**All participants**	23,801		143,890	
**Time from index date† to end of follow-up (years)**				
Mean (SD)	5.71 (4.27)		6.95 (4.70)	
Median (IQR)	4.44 (2.30- 8.02)		5.83 (3.08- 9.86)	
Range	1.00-21.07		1.00-21.08	
**Total person-years ‡**				
	135926.7		999528.5	
**Age (years)**				
Mean (SD)	63.09 (15.14)		63.31 (15.26)	
Median (IQR)	65 (53-74)		65 (54-75)	
**Age group at cancer diagnosis (years)**				
18 - 59	8,779	(36.89)	51,927	(36.09)
60 - 79	11,854	(49.80)	72,047	(50.07)
≥80	3,168	(13.31)	19,916	(13.84)
**Sex**				
Women	11,135	(46.78)	66,021	(45.88)
Men	12,666	(53.22)	77,869	(54.12)
**Patient-level Index of Multiple Deprivation (practice postcode-based)**				
1 (least deprived)	5,584	(23.46)	33,739	(23.45)
2	5,382	(22.61)	31,829	(22.12)
3	4,671	(19.63)	28,856	(20.05)
4	4,380	(18.40)	26,500	(18.42)
5 (most deprived)	3,762	(15.81)	22,854	(15.88)
**Calendar year of cancer diagnosis**				
1999 - 2003	4,541	(19.08)	27,060	(18.81)
2004 - 2008	6,016	(25.28)	36,237	(25.18)
2009 - 2013	6,634	(27.87)	40,157	(27.91)
2014 - 2019	6,610	(27.77)	40,436	(28.10)
**Ethnicity**				
White	14,518	(61.00)	90,941	(63.20)
South Asian	649	(2.73)	4,464	(3.10)
Black	647	(2.72)	3,144	(2.19)
Other	307	(1.29)	1,784	(1.24)
Unknown	7,680	(32.27)	43,557	(30.27)
**Body mass index**				
Underweight (<18.5)	512	(2.15)	2,169	(1.51)
Normal weight (18.5-24.9)	8,617	(36.20)	45,442	(31.58)
Overweight (25.0-29.9)	8,221	(34.54)	48,910	(33.99)
Obese (≥30.0)	4,861	(20.42)	30,458	(21.17)
Unknown	1,590	(6.68)	16,911	(11.75)
**Alcohol consumption**				
Non-drinker	2,566	(10.78)	13,850	(9.63)
Current drinker	12,486	(52.46)	74,764	(51.96)
Ex-drinker	1,023	(4.30)	4,983	(3.46)

Unknown	7,726	(32.46)	50,293	(34.95)
**Problem drinker**				
	626	(2.63)	4,455	(3.10)
**Smoking status**				
Non-smoker	8,110	(34.07)	48,775	(33.90)
Current smoker	3,488	(14.65)	26,437	(18.37)
Ex-smoker	11,775	(49.47)	64,512	(44.83)
Unknown	428	(1.80)	4,166	(2.90)
**Prescribed**				
Oral corticosteroids	5,513	(23.16)	22,362	(15.54)
Hormone replacement therapy	3,841	(16.14)	19,624	(13.64)
Bisphosphonate therapy	799	(3.36)	3,651	(2.54)
**History of**				
Fracture §	5,209	(21.89)	30,774	(21.39)
Osteoporosis	1,209	(5.08)	6,239	(4.34)
Eating disorder	87	(0.37)	477	(0.33)
**Comorbidities**				
Coeliac disease	161	(0.68)	487	(0.34)
Inflammatory bowel disease	1,606	(6.75)	4,416	(3.07)
Systemic lupus erythematosus	151	(0.63)	258	(0.18)
Rheumatoid arthritis	809	(3.40)	2,390	(1.66)
Chronic kidney disease	5,492	(23.07)	26,673	(18.54)
Liver disease	617	(2.59)	1,399	(0.97)
Epilepsy	505	(2.12)	2,451	(1.70)

SD = standard deviation; IQR = interquartile range.
* Data are n (%) unless otherwise stated. The characteristics of the study participants were measured prior to or near the index date†. When information on body mass index, height and weight, alcohol consumption, and smoking status was unavailable prior to the index date, we used information recorded at any point in the clinical record. We ran a sensitivity analysis using only pre-index data.
† For cancer survivors, the index date was the date of their cancer diagnosis. In the matched cancer-free cohort, the matched cancer survivor's index date was allocated to each individual. Cancer-free people were individually matched on year of birth (+/- 3 years), sex and general practice to participants in the cancer survivor cohort.
‡ Index date to end of follow-up.
§ In adulthood.

Table S21: Characteristics of cancer survivors and matched controls from the general population (multiple myeloma; ICD-10 C90).*

	Cancer survivors		Cancer-free controls	
**All participants**	10,916		61,228	
**Time from index date† to end of follow-up (years)**				
Mean (SD)	4.38 (3.30)		6.58 (4.55)	
Median (IQR)	3.38 (1.97- 5.70)		5.42 (2.90- 9.22)	
Range	1.00-20.92		1.00-21.08	
**Total person-years ‡**	47864.58		402932.1	
**Age (years)**				
Mean (SD)	69.03 (12.05)		69.27 (12.02)	
Median (IQR)	70 (61-78)		71 (62-78)	
**Age group at cancer diagnosis (years)**				
18 - 59	2,297	(21.04)	12,632	(20.63)
60 - 79	6,405	(58.68)	35,725	(58.35)
≥80	2,214	(20.28)	12,871	(21.02)
**Sex**				
Women	5,035	(46.12)	27,923	(45.60)
Men	5,881	(53.88)	33,305	(54.40)
**Patient-level Index of Multiple Deprivation (practice postcode-based)**				
1 (least deprived)	2,551	(23.37)	14,010	(22.88)
2	2,448	(22.43)	13,561	(22.15)
3	2,264	(20.74)	12,222	(19.96)
4	1,961	(17.96)	11,535	(18.84)
5 (most deprived)	1,688	(15.46)	9,852	(16.09)
**Calendar year of cancer diagnosis**				
1999 - 2003	2,104	(19.27)	12,114	(19.79)
2004 - 2008	2,531	(23.19)	14,472	(23.64)
2009 - 2013	2,918	(26.73)	16,551	(27.03)
2014 - 2019	3,363	(30.81)	18,091	(29.55)
**Ethnicity**				
White	5,989	(54.86)	38,793	(63.36)
South Asian	290	(2.66)	1,802	(2.94)
Black	511	(4.68)	1,481	(2.42)
Other	105	(0.96)	612	(1.00)
Unknown	4,021	(36.84)	18,540	(30.28)
**Body mass index**				
Underweight (<18.5)	244	(2.24)	979	(1.60)
Normal weight (18.5-24.9)	3,667	(33.59)	18,775	(30.66)
Overweight (25.0-29.9)	3,966	(36.33)	21,033	(34.35)
Obese (≥30.0)	2,229	(20.42)	13,078	(21.36)
Unknown	810	(7.42)	7,363	(12.03)
**Alcohol consumption**				
Non-drinker	1,117	(10.23)	6,022	(9.84)
Current drinker	5,632	(51.59)	32,459	(53.01)
Ex-drinker	550	(5.04)	2,454	(4.01)

Unknown	3,617	(33.13)	20,293	(33.14)
**Problem drinker**				
	247	(2.26)	1,716	(2.80)
**Smoking status**				
Non-smoker	3,754	(34.39)	20,219	(33.02)
Current smoker	1,292	(11.84)	10,667	(17.42)
Ex-smoker	5,646	(51.72)	28,678	(46.84)
Unknown	224	(2.05)	1,664	(2.72)
**Prescribed**				
Oral corticosteroids	2,931	(26.85)	10,695	(17.47)
Hormone replacement therapy	1,580	(14.47)	7,763	(12.68)
Bisphosphonate therapy	702	(6.43)	2,041	(3.33)
**History of**				
Fracture §	3,233	(29.62)	13,355	(21.81)
Osteoporosis	1,092	(10.00)	3,411	(5.57)
Eating disorder	45	(0.41)	172	(0.28)
**Comorbidities**				
Coeliac disease	56	(0.51)	223	(0.36)
Inflammatory bowel disease	1,036	(9.49)	2,053	(3.35)
Systemic lupus erythematosus	42	(0.38)	113	(0.18)
Rheumatoid arthritis	314	(2.88)	1,315	(2.15)
Chronic kidney disease	4,630	(42.41)	14,970	(24.45)
Liver disease	256	(2.35)	662	(1.08)
Epilepsy	233	(2.13)	998	(1.63)

SD = standard deviation; IQR = interquartile range.
* Data are n (%) unless otherwise stated. The characteristics of the study participants were measured prior to or near the index date†. When information on body mass index, height and weight, alcohol consumption, and smoking status was unavailable prior to the index date, we used information recorded at any point in the clinical record. We ran a sensitivity analysis using only pre-index data.
† For cancer survivors, the index date was the date of their cancer diagnosis. In the matched cancer-free cohort, the matched cancer survivor's index date was allocated to each individual. Cancer-free people were individually matched on year of birth (+/- 3 years), sex and general practice to participants in the cancer survivor cohort.
‡ Index date to end of follow-up.
§ In adulthood.

Table S22: Characteristics of cancer survivors and matched controls from the general population (leukaemia; ICD-10 C91-95).*

	Cancer survivors		Cancer-free controls	
**All participants**	19,611		111,149	
**Time from index date† to end of follow-up (years)**				
Mean (SD)	5.48 (4.15)		6.84 (4.66)	
Median (IQR)	4.20 (2.20- 7.63)		5.67 (3.03- 9.74)	
Range	1.00-21.07		1.00-21.07	
**Total person-years ‡**				
	107546.4		760387.2	
**Age (years)**				
Mean (SD)	66.62 (14.44)		66.94 (14.39)	
Median (IQR)	68 (58-77)		69 (59-77)	
**Age group at cancer diagnosis (years)**				
18 - 59	5,343	(27.24)	29,429	(26.48)
60 - 79	10,561	(53.85)	60,020	(54.00)
≥80	3,707	(18.90)	21,700	(19.52)
**Sex**				
Women	8,273	(42.19)	46,462	(41.80)
Men	11,338	(57.81)	64,687	(58.20)
**Patient-level Index of Multiple Deprivation (practice postcode-based)**				
1 (least deprived)	4,735	(24.14)	26,356	(23.71)
2	4,487	(22.88)	25,236	(22.70)
3	3,931	(20.04)	22,282	(20.05)
4	3,487	(17.78)	20,404	(18.36)
5 (most deprived)	2,949	(15.04)	16,760	(15.08)
**Calendar year of cancer diagnosis**				
1999 - 2003	4,181	(21.32)	23,724	(21.34)
2004 - 2008	4,935	(25.16)	28,455	(25.60)
2009 - 2013	5,061	(25.81)	28,978	(26.07)
2014 - 2019	5,434	(27.71)	29,992	(26.98)
**Ethnicity**				
White	12,104	(61.72)	69,463	(62.50)
South Asian	402	(2.05)	2,964	(2.67)
Black	241	(1.23)	1,764	(1.59)
Other	138	(0.70)	1,074	(0.97)
Unknown	6,726	(34.30)	35,884	(32.28)
**Body mass index**				
Underweight (<18.5)	321	(1.64)	1,794	(1.61)
Normal weight (18.5-24.9)	6,498	(33.13)	33,986	(30.58)
Overweight (25.0-29.9)	7,117	(36.29)	38,334	(34.49)
Obese (≥30.0)	4,255	(21.70)	22,701	(20.42)
Unknown	1,420	(7.24)	14,334	(12.90)
**Alcohol consumption**				
Non-drinker	1,885	(9.61)	10,476	(9.43)
Current drinker	10,626	(54.18)	58,306	(52.46)
Ex-drinker	901	(4.59)	4,091	(3.68)

Unknown	6,199	(31.61)	38,276	(34.44)
**Problem drinker**				
	493	(2.51)	3,286	(2.96)
**Smoking status**				
Non-smoker	6,387	(32.57)	36,584	(32.91)
Current smoker	2,604	(13.28)	19,853	(17.86)
Ex-smoker	10,234	(52.18)	51,365	(46.21)
Unknown	386	(1.97)	3,347	(3.01)
**Prescribed**				
Oral corticosteroids	4,120	(21.01)	18,191	(16.37)
Hormone replacement therapy	2,638	(13.45)	12,624	(11.36)
Bisphosphonate therapy	621	(3.17)	3,268	(2.94)
**History of**				
Fracture §	4,427	(22.57)	24,241	(21.81)
Osteoporosis	977	(4.98)	5,190	(4.67)
Eating disorder	70	(0.36)	311	(0.28)
**Comorbidities**				
Coeliac disease	73	(0.37)	413	(0.37)
Inflammatory bowel disease	1,286	(6.56)	3,583	(3.22)
Systemic lupus erythematosus	58	(0.30)	197	(0.18)
Rheumatoid arthritis	496	(2.53)	2,053	(1.85)
Chronic kidney disease	5,460	(27.84)	24,319	(21.88)
Liver disease	418	(2.13)	1,050	(0.94)
Epilepsy	389	(1.98)	1,887	(1.70)

SD = standard deviation; IQR = interquartile range.
* Data are n (%) unless otherwise stated. The characteristics of the study participants were measured prior to or near the index date†. When information on body mass index, height and weight, alcohol consumption, and smoking status was unavailable prior to the index date, we used information recorded at any point in the clinical record. We ran a sensitivity analysis using only pre-index data.
† For cancer survivors, the index date was the date of their cancer diagnosis. In the matched cancer-free cohort, the matched cancer survivor's index date was allocated to each individual. Cancer-free people were individually matched on year of birth (+/- 3 years), sex and general practice to participants in the cancer survivor cohort.
‡ Index date to end of follow-up.
§ In adulthood.

Table S23: Bone fracture and major osteoporotic fracture incidence in survivors of the 20 most common types of cancers and cancer-free individuals.

					Bone fractures				Major osteoporotic fracture			
	Cancer survivors		Cancer-free controls		Cancer survivors		Cancer-free controls		Cancer survivors		Cancer-free controls	
**Cancer type or site (ICD-10)**	**No. in analysis**	**PY at risk**	**No. in analysis**	**PY at risk**	**No. of events**	**Rate per 1000 PY (95% CI)**	**No. of events**	**Rate per 1000 PY (95% CI)**	**No. of events**	**Rate per 1000 PY (95% CI)**	**No. of events**	**Rate per 1000 PY (95% CI)**
Oral cavity (C00-06)	5,266	24.55	31,631	188.76	297	12.10 (10.80-13.56)	1,926	10.20 (9.76-10.67)	140	5.70 (4.83-6.73)	777	4.12 (3.84-4.42)
Oesophagus (C15)	8,048	20.96	66,543	387.73	274	13.07 (11.61-14.72)	4,489	11.58 (11.24-11.92)	141	6.73 (5.70-7.94)	2,140	5.52 (5.29-5.76)
Stomach (C16)	5,436	17.73	41,253	242.44	272	15.34 (13.62-17.28)	3,154	13.01 (12.56-13.47)	160	9.03 (7.73-10.54)	1,558	6.43 (6.11-6.75)
Colorectal (C18-20)	67,876	305.12	384,129	2,240.65	4,028	13.20 (12.80-13.62)	28,676	12.80 (12.65-12.95)	2,139	7.01 (6.72-7.31)	14,223	6.35 (6.24-6.45)
Liver (C22)	3,420	7.98	25,502	126.57	122	15.30 (12.81-18.27)	1,408	11.12 (10.56-11.72)	54	6.77 (5.19-8.84)	675	5.33 (4.95-5.75)
Pancreas (C25)	4,198	9.22	35,778	194.37	149	16.16 (13.76-18.97)	2,797	14.39 (13.87-14.93)	69	7.48 (5.91-9.47)	1,361	7.00 (6.64-7.38)
Lung (C34)	32,039	71.23	211,485	1164.36	1,236	17.35 (16.41-18.35)	15,662	13.45 (13.24-13.66)	646	9.07 (8.40-9.80)	7,703	6.62 (6.47-6.77)
Malignant melanoma (C43)	41,705	227.02	219,667	1,287.91	2,478	10.92 (10.49-11.35)	13,454	10.45 (10.27-10.62)	1,025	4.52 (4.25-4.80)	5,827	4.52 (4.41-4.64)
Breast (C50)	145,515	822.16	742,221	4,711.69	13,424	16.33 (16.05-16.61)	65,783	13.96 (13.86-14.07)	5,734	6.97 (6.80-7.16)	28,577	6.07 (6.00-6.14)
Cervix (C53)	6,267	34.43	35,698	236.75	375	10.89 (9.84-12.05)	2,483	10.49 (10.08-10.91)	157	4.56 (3.90-5.33)	924	3.90 (3.66-4.16)
Uterus (C54-55)	13,970	73.84	79,585	475.1	991	13.42 (12.61-14.28)	7,154	15.06 (14.71-15.41)	509	6.89 (6.32-7.52)	3,396	7.15 (6.91-7.39)
Ovary (C56)	12,475	57.29	85,766	555.85	788	13.76 (12.83-14.75)	7,969	14.34 (14.03-14.65)	331	5.78 (5.19-6.44)	3,591	6.46 (6.25-6.68)
Prostate (C61)	117,875	565.51	612,935	3,364.13	6,956	12.30 (12.01-12.59)	30,190	8.97 (8.87- 9.08)	3,569	6.31 (6.11-6.52)	14,604	4.34 (4.27-4.41)
Kidney (C64)	10,819	45.42	64,193	352.98	617	13.58 (12.55-14.70)	3,955	11.20 (10.86-11.56)	283	6.23 (5.55-7.00)	1,852	5.25 (5.01-5.49)
Bladder (C67)	36,820	180.14	204,026	1170.11	2,142	11.89 (11.40-12.41)	13,382	11.44 (11.24-11.63)	1,148	6.37 (6.01-6.75)	6,596	5.64 (5.50-5.77)
CNS (C71-72)	6,236	21.31	38,949	239.09	264	12.39 (10.98-13.97)	2,235	9.35 (8.97- 9.74)	105	4.93 (4.07-5.96)	808	3.38 (3.15-3.62)
Thyroid (C73)	5,867	32.06	30,776	172.11	269	8.39 (7.45-9.46)	1,638	9.52 (9.07- 9.99)	122	3.81 (3.19-4.54)	599	3.48 (3.21-3.77)
NHL (C82-85)	23,801	112.13	143,890	855.64	1,326	11.83 (11.21-12.48)	9,508	11.11 (10.89-11.34)	669	5.97 (5.53-6.44)	4,294	5.02 (4.87-5.17)
Multiple myeloma (C90)	10,916	36.95	61,228	341.7	799	21.62 (20.18-23.18)	4,311	12.62 (12.25-13.00)	398	10.77 (9.76-11.88)	2,179	6.38 (6.11-6.65)
Leukaemia (C91-95)	19,611	87.94	111,149	649.24	1,077	12.25 (11.54-13.00)	7,760	11.95 (11.69-12.22)	561	6.38 (5.87-6.93)	3,679	5.67 (5.49-5.85)

CNS = Central Nervous System; NHL = Non-Hodgkin Lymphoma; PY = person-years; CI = confidence interval.

Table S24: Major osteoporotic pelvic fracture incidence in survivors of the 20 most common types of cancers and cancer-free controls.

	Cancer survivors		Cancer-free controls		Cancer survivors		Cancer-free controls	
**Cancer type or site (ICD-10)**	**No. in analysis**	**PY at risk***	**No. in analysis**	**PY at risk**	**No. of events**	**Rate per 1,000 PY (95% CI)**	**No. of events**	**Rate per 1,000 PY (95% CI)**
Oral cavity (C00-06)	5,266	24.55	31,631	188.76	10	0.41 (0.22-0.76)	95	0.50 (0.41-0.62)
Oesophagus (C15)	8,048	20.96	66,543	387.73	11	0.52 (0.29-0.95)	233	0.60 (0.53-0.68)
Stomach (C16)	5,436	17.73	41,253	242.44	18	1.02 (0.64-1.61)	157	0.65 (0.55-0.76)
Colorectal (C18-20)	67,876	305.12	384,129	2,240.65	221	0.72 (0.63-0.83)	1,376	0.61 (0.58-0.65)
Liver (C22)	3,420	7.98	25,502	126.57	8	1.00 (0.50-2.01)	66	0.52 (0.41-0.66)
Pancreas (C25)	4,198	9.22	35,778	194.37	5	0.54 (0.23-1.30)	127	0.65 (0.55-0.78)
Lung (C34)	32,039	71.23	211,485	1,164.36	57	0.80 (0.62-1.04)	683	0.59 (0.54-0.63)
Malignant melanoma (C43)	41,705	227.02	219,667	1,287.91	87	0.38 (0.31-0.47)	504	0.39 (0.36-0.43)
Breast (C50)	145,515	822.16	742,221	4,711.69	525	0.64 (0.59-0.70)	2,513	0.53 (0.51-0.55)
Cervix (C53)	6,267	34.43	35,698	236.75	28	0.81 (0.56-1.18)	75	0.32 (0.25-0.40)
Uterus (C54-55)	13,970	73.84	79,585	475.1	53	0.72 (0.55-0.94)	302	0.64 (0.57-0.71)
Ovary (C56)	12,475	57.29	85,766	555.85	25	0.44 (0.29-0.65)	353	0.64 (0.57-0.70)
Prostate (C61)	117,875	565.51	612,935	3,364.13	311	0.55 (0.49-0.61)	1,258	0.37 (0.35-0.40)
Kidney (C64)	10,819	45.42	64,193	352.98	31	0.68 (0.48-0.97)	190	0.54 (0.47-0.62)
Bladder (C67)	36,820	180.14	204,026	1,170.11	123	0.68 (0.57-0.81)	630	0.54 (0.50-0.58)
CNS (C71-72)	6,236	21.31	38,949	239.09	13	0.61 (0.35-1.05)	57	0.24 (0.18-0.31)
Thyroid (C73)	5,867	32.06	30,776	172.11	11	0.34 (0.19-0.62)	43	0.25 (0.19-0.34)
NHL (C82-85)	23,801	112.13	143,890	855.64	90	0.80 (0.65-0.99)	365	0.43 (0.38-0.47)
Multiple myeloma (C90)	10,916	36.95	61,228	341.7	39	1.06 (0.77-1.44)	212	0.62 (0.54-0.71)
Leukaemia (C91-95)	19,611	87.94	111,149	649.24	73	0.83 (0.66-1.04)	361	0.56 (0.50-0.62)

CNS = Central Nervous System; NHL = Non-Hodgkin Lymphoma; PY = person-years; CI = confidence interval.
* in millions.

Table S25: Major osteoporotic hip fracture incidence in survivors of the 20 most common types of cancers and cancer-free controls.

	Cancer survivors		Cancer-free controls		Cancer survivors		Cancer-free controls	
**Cancer type or site (ICD-10)**	**No. in analysis**	**PY at risk***	**No. in analysis**	**PY at risk**	**No. of events**	**Rate per 1,000 PY (95% CI)**	**No. of events**	**Rate per 1,000 PY (95% CI)**
Oral cavity (C00-06)	5,266	24.55	31,631	188.76	53	2.16 (1.65- 2.83)	272	1.44 (1.28- 1.62)
Oesophagus (C15)	8,048	20.96	66,543	387.73	50	2.39 (1.81- 3.15)	920	2.37 (2.22- 2.53)
Stomach (C16)	5,436	17.73	41,253	242.44	64	3.61 (2.83- 4.61)	727	3.00 (2.79- 3.22)
Colorectal (C18-20)	67,876	305.12	384,129	2,240.65	803	2.63 (2.46- 2.82)	6,026	2.69 (2.62- 2.76)
Liver (C22)	3,420	7.98	25,502	126.57	22	2.76 (1.82- 4.19)	281	2.22 (1.98- 2.50)
Pancreas (C25)	4,198	9.22	35,778	194.37	34	3.69 (2.63- 5.16)	627	3.23 (2.98- 3.49)
Lung (C34)	32,039	71.23	211,485	1,164.36	271	3.80 (3.38- 4.29)	3,368	2.89 (2.80- 2.99)
Malignant melanoma (C43)	41,705	227.02	219,667	1,287.91	311	1.37 (1.23- 1.53)	1,961	1.52 (1.46- 1.59)
Breast (C50)	145,515	822.16	742,221	4,711.69	1,573	1.91 (1.82- 2.01)	8,753	1.86 (1.82- 1.90)
Cervix (C53)	6,267	34.43	35,698	236.75	44	1.28 (0.95- 1.72)	246	1.04 (0.92- 1.18)
Uterus (C54-55)	13,970	73.84	79,585	475.1	123	1.67 (1.40- 1.99)	1,061	2.23 (2.10- 2.37)
Ovary (C56)	12,475	57.29	85,766	555.85	95	1.66 (1.36- 2.03)	1,080	1.94 (1.83- 2.06)
Prostate (C61)	117,875	565.51	612,935	3,364.13	1,538	2.72 (2.59- 2.86)	7,011	2.08 (2.04- 2.13)
Kidney (C64)	10,819	45.42	64,193	352.98	103	2.27 (1.87- 2.75)	670	1.90 (1.76- 2.05)
Bladder (C67)	36,820	180.14	204,026	1,170.11	490	2.72 (2.49- 2.97)	2,932	2.51 (2.42- 2.60)
CNS (C71-72)	6,236	21.31	38,949	239.09	26	1.22 (0.83- 1.79)	297	1.24 (1.11- 1.39)
Thyroid (C73)	5,867	32.06	30,776	172.11	25	0.78 (0.53- 1.15)	166	0.96 (0.83- 1.12)
NHL (C82-85)	23,801	112.13	143,890	855.64	247	2.20 (1.94- 2.50)	1,601	1.87 (1.78- 1.97)
Multiple myeloma (C90)	10,916	36.95	61,228	341.7	150	4.06 (3.46- 4.76)	894	2.62 (2.45- 2.79)
Leukaemia (C91-95)	19,611	87.94	111,149	649.24	198	2.25 (1.96- 2.59)	1,523	2.35 (2.23- 2.47)

CNS = Central Nervous System; NHL = Non-Hodgkin Lymphoma; PY = person-years; CI = confidence interval.
* in millions.

Table S26: Major osteoporotic wrist fracture incidence in survivors of the 20 most common types of cancers and cancer-free controls.

	Cancer survivors		Cancer-free controls		Cancer survivors		Cancer-free controls	
**Cancer type or site (ICD-10)**	**No. in analysis**	**PY at risk***	**No. in analysis**	**PY at risk**	**No. of events**	**Rate per 1,000 PY (95% CI)**	**No. of events**	**Rate per 1,000 PY (95% CI)**
Oral cavity (C00-06)	5,266	24.55	31,631	188.76	39	1.59 (1.16- 2.17)	249	1.32 (1.17- 1.49)
Oesophagus (C15)	8,048	20.96	66,543	387.73	24	1.15 (0.77- 1.71)	514	1.33 (1.22- 1.45)
Stomach (C16)	5,436	17.73	41,253	242.44	47	2.65 (1.99- 3.53)	366	1.51 (1.36- 1.67)
Colorectal (C18-20)	67,876	305.12	384,129	2,240.65	607	1.99 (1.84- 2.15)	3,830	1.71 (1.66- 1.76)
Liver (C22)	3,420	7.98	25,502	126.57	9	1.13 (0.59- 2.17)	177	1.40 (1.21- 1.62)
Pancreas (C25)	4,198	9.22	35,778	194.37	16	1.73 (1.06- 2.83)	365	1.88 (1.69- 2.08)
Lung (C34)	32,039	71.23	211,485	1,164.36	140	1.97 (1.67- 2.32)	2,040	1.75 (1.68- 1.83)
Malignant melanoma (C43)	41,705	227.02	219,667	1,287.91	393	1.73 (1.57- 1.91)	2,039	1.58 (1.52- 1.65)
Breast (C50)	145,515	822.16	742,221	4,711.69	2,431	2.96 (2.84- 3.08)	11,672	2.48 (2.43- 2.52)
Cervix (C53)	6,267	34.43	35,698	236.75	52	1.51 (1.15- 1.98)	432	1.82 (1.66- 2.01)
Uterus (C54-55)	13,970	73.84	79,585	475.10	219	2.97 (2.60- 3.39)	1,333	2.81 (2.66- 2.96)
Ovary (C56)	12,475	57.29	85,766	555.85	149	2.60 (2.22- 3.05)	1,477	2.66 (2.53- 2.80)
Prostate (C61)	117,875	565.51	612,935	3,364.13	711	1.26 (1.17- 1.35)	2,435	0.72 (0.70- 0.75)
Kidney (C64)	10,819	45.42	64,193	352.98	71	1.56 (1.24- 1.97)	573	1.62 (1.50- 1.76)
Bladder (C67)	36,820	180.14	204,026	1,170.11	258	1.43 (1.27- 1.62)	1,508	1.29 (1.23- 1.36)
CNS (C71-72)	6,236	21.31	38,949	239.09	40	1.88 (1.38- 2.56)	297	1.24 (1.11- 1.39)
Thyroid (C73)	5,867	32.06	30,776	172.11	51	1.59 (1.21- 2.09)	263	1.53 (1.35- 1.72)
NHL (C82-85)	23,801	112.13	143,890	855.64	174	1.55 (1.34- 1.80)	1,370	1.60 (1.52- 1.69)
Multiple myeloma (C90)	10,916	36.95	61,228	341.70	34	0.92 (0.66- 1.29)	597	1.75 (1.61- 1.89)
Leukaemia (C91-95)	19,611	87.94	111,149	649.24	136	1.55 (1.31- 1.83)	1,023	1.58 (1.48- 1.68)

CNS = Central Nervous System; NHL = Non-Hodgkin Lymphoma; PY = person-years; CI = confidence interval.
* in millions.

Table S27: Major osteoporotic spine fracture incidence in survivors of the 20 most common types of cancers and cancer-free controls.

	Cancer survivors		Cancer-free controls		Cancer survivors		Cancer-free controls	
**Cancer type or site (ICD-10)**	**No. in analysis**	**PY at risk**	**No. in analysis**	**PY at risk**	**No. of events**	**Rate per 1,000 PY (95% CI)**	**No. of events**	**Rate per 1,000 PY (95% CI)**
Oral cavity (C00-06)	5,266	24.55	31,631	188.76	27	1.10 (0.75- 1.60)	128	0.68 (0.57- 0.81)
Oesophagus (C15)	8,048	20.96	66,543	387.73	47	2.24 (1.69- 2.98)	379	0.98 (0.88- 1.08)
Stomach (C16)	5,436	17.73	41,253	242.44	24	1.35 (0.91- 2.02)	235	0.97 (0.85- 1.10)
Colorectal (C18-20)	67,876	305.12	384,129	2240.65	408	1.34 (1.21- 1.47)	2,275	1.02 (0.97- 1.06)
Liver (C22)	3,420	7.98	25,502	126.57	12	1.50 (0.85- 2.65)	117	0.92 (0.77- 1.11)
Pancreas (C25)	4,198	9.22	35,778	194.37	14	1.52 (0.90- 2.56)	212	1.09 (0.95- 1.25)
Lung (C34)	32,039	71.23	211,485	1164.36	144	2.02 (1.72- 2.38)	1,321	1.13 (1.07- 1.20)
Malignant melanoma (C43)	41,705	227.02	219,667	1287.91	172	0.76 (0.65- 0.88)	980	0.76 (0.71- 0.81)
Breast (C50)	145,515	822.16	742,221	4711.69	900	1.09 (1.03- 1.17)	3,930	0.83 (0.81- 0.86)
Cervix (C53)	6,267	34.43	35,698	236.75	23	0.67 (0.44- 1.01)	113	0.48 (0.40- 0.57)
Uterus (C54-55)	13,970	73.84	79,585	475.1	69	0.93 (0.74- 1.18)	491	1.03 (0.95- 1.13)
Ovary (C56)	12,475	57.29	85,766	555.85	47	0.82 (0.62- 1.09)	465	0.84 (0.76- 0.92)
Prostate (C61)	117,875	565.51	612,935	3364.13	836	1.48 (1.38- 1.58)	3,205	0.95 (0.92- 0.99)
Kidney (C64)	10,819	45.42	64,193	352.98	61	1.34 (1.04- 1.73)	328	0.93 (0.83- 1.04)
Bladder (C67)	36,820	180.14	204,026	1170.11	211	1.17 (1.02- 1.34)	1,208	1.03 (0.98- 1.09)
CNS (C71-72)	6,236	21.31	38,949	239.09	22	1.03 (0.68- 1.57)	129	0.54 (0.45- 0.64)
Thyroid (C73)	5,867	32.06	30,776	172.11	28	0.87 (0.60- 1.26)	93	0.54 (0.44- 0.66)
NHL (C82-85)	23,801	112.13	143,890	855.64	129	1.15 (0.97- 1.37)	751	0.88 (0.82- 0.94)
Multiple myeloma (C90)	10,916	36.95	61,228	341.7	163	4.41 (3.78- 5.14)	384	1.12 (1.02- 1.24)
Leukaemia (C91-95)	19,611	87.94	111,149	649.24	125	1.42 (1.19- 1.69)	586	0.90 (0.83- 0.98)

CNS = Central Nervous System; NHL = Non-Hodgkin Lymphoma; PY = person-years; CI = confidence interval.
* in millions.

Table S28: Major osteoporotic proximal humerus fracture incidence in survivors of the 20 most common types of cancers and cancer-free controls.

	Cancer survivors		Cancer-free controls		Cancer survivors		Cancer-free controls	
**Cancer type or site (ICD-10)**	**No. in analysis**	**PY at risk***	**No. in analysis**	**PY at risk**	**No. of events**	**Rate per 1,000 PY (95% CI)**	**No. of events**	**Rate per 1,000 PY (95% CI)**
Oral cavity (C00-06)	5,266	24.55	31,631	188.76	11	0.45 (0.25- 0.81)	50	0.26 (0.20- 0.35)
Oesophagus (C15)	8,048	20.96	66,543	387.73	13	0.62 (0.36- 1.07)	138	0.36 (0.30- 0.42)
Stomach (C16)	5,436	17.73	41,253	242.44	9	0.51 (0.26- 0.98)	103	0.42 (0.35- 0.52)
Colorectal (C18-20)	67,876	305.12	384,129	2,240.65	129	0.42 (0.36- 0.50)	950	0.42 (0.40- 0.45)
Liver (C22)	3,420	7.98	25,502	126.57	4	0.50 (0.19- 1.34)	43	0.34 (0.25- 0.46)
Pancreas (C25)	4,198	9.22	35,778	194.37	3	0.33 (0.10- 1.01)	69	0.35 (0.28- 0.45)
Lung (C34)	32,039	71.23	211,485	1,164.36	38	0.53 (0.39- 0.73)	459	0.39 (0.36- 0.43)
Malignant melanoma (C43)	41,705	227.02	219,667	1,287.91	83	0.37 (0.29- 0.45)	423	0.33 (0.30- 0.36)
Breast (C50)	145,515	822.16	742,221	4,711.69	402	0.49 (0.44- 0.54)	2188	0.46 (0.45- 0.48)
Cervix (C53)	6,267	34.43	35,698	236.75	11	0.32 (0.18- 0.58)	76	0.32 (0.26- 0.40)
Uterus (C54-55)	13,970	73.84	79,585	475.10	51	0.69 (0.52- 0.91)	270	0.57 (0.50- 0.64)
Ovary (C56)	12,475	57.29	85,766	555.85	22	0.38 (0.25- 0.58)	278	0.50 (0.44- 0.56)
Prostate (C61)	117,875	565.51	612,935	3,364.13	219	0.39 (0.34- 0.44)	860	0.26 (0.24- 0.27)
Kidney (C64)	10,819	45.42	64,193	352.98	19	0.42 (0.27- 0.66)	123	0.35 (0.29- 0.42)
Bladder (C67)	36,820	180.14	204,026	1,170.11	74	0.41 (0.33- 0.52)	421	0.36 (0.33- 0.40)
CNS (C71-72)	6,236	21.31	38,949	239.09	5	0.23 (0.10- 0.56)	55	0.23 (0.18- 0.30)
Thyroid (C73)	5,867	32.06	30,776	172.11	9	0.28 (0.15- 0.54)	39	0.23 (0.17- 0.31)
NHL (C82-85)	23,801	112.13	143,890	855.64	42	0.37 (0.28- 0.51)	272	0.32 (0.28- 0.36)
Multiple myeloma (C90)	10,916	36.95	61,228	341.70	14	0.38 (0.22- 0.64)	124	0.36 (0.30- 0.43)
Leukaemia (C91-95)	19,611	87.94	111,149	649.24	36	0.41 (0.30- 0.57)	243	0.37 (0.33- 0.42)

CNS = Central Nervous System; NHL = Non-Hodgkin Lymphoma; PY = person-years; CI = confidence interval.
* in millions.

Table S29: Estimated cumulative risk of any bone fracture and major osteoporotic fracture in cancer survivors at 5 and 10 years after cancer diagnosis. Follow up started at 1 year.

		Cumulative incidence (%)			
		Any fracture		Major osteoporotic fracture	
**Cancer type or site (ICD-10)**	**Years after diagnosis**	**Men**	**Women**	**Men**	**Women**
Oral cavity (C00-06)	5	3.3	6.1	1.3	2.9
	10	8.0	14.5	3.4	7.5
Oesophagus (C15)	5	3.7	7.3	1.8	3.6
	10	9.5	18.0	5.3	10.3
Stomach (C16)	5	4.6	8.3	2.6	4.9
	10	11.2	19.6	7.2	13.3
Colorectal (C18-20)	5	3.3	6.1	1.6	3.0
	10	8.4	15.2	4.5	8.6
Liver (C22)	5	5.3	10.1	2.5	4.7
	10	12.6	22.6	6.7	12.2
Pancreas (C25)	5	4.5	8.9	2.3	4.6
	10	11.0	20.6	5.8	11.3
Lung (C34)	5	4.7	9.4	2.6	5.0
	10	11.7	22.3	7.1	13.5
Malignant melanoma (C43)	5	2.9	5.2	1.1	2.2
	10	7.1	12.4	3.1	5.8
Breast (C50)	5	-	5.6	-	2.2
	10	-	13.8	-	6.2
Cervix (C53)	5	-	4.6	-	2.3
	10	-	10.4	-	5.0
Uterus (C54-55)	5	-	4.6	-	2.2
	10	-	11.2	-	6.0
Ovary (C56)	5	-	5.2	-	2.2
	10	-	12.6	-	5.9
Prostate (C61)	5	4.2	-	2.0	-
	10	11.1	-	6.1	-
Kidney (C64)	5	4.0	7.3	1.7	3.3
	10	9.6	17.1	4.8	8.7
Bladder (C67)	5	3.4	6.4	1.7	3.3
	10	8.9	16.1	5.1	9.6
CNS (C71-72)	5	4.0	7.2	1.4	3.1
	10	9.1	15.9	3.9	8.3
Thyroid (C73)	5	1.8	3.2	0.8	1.4
	10	4.6	7.9	2.2	3.9
NHL (C82-85)	5	3.2	5.8	1.5	2.7
	10	8.0	14.1	4.2	7.7
Multiple myeloma (C90)	5	5.6	9.7	2.7	5.0
	10	14.3	23.8	7.8	13.9
Leukaemia (C91-95)	5	3.3	6.3	1.6	3.1
	10	8.2	15.4	4.4	8.6

Cumulative incidence predicted from a Royston-Parmar model including age, sex (when applicable), BMI (cubic spline), index of multiple deprivation, smoking, problematic alcohol use, use of corticosteroids, bisphosphonates, and hormone replacement therapy, and history of chronic kidney disease, liver disease, epilepsy, and autoimmune conditions, with the baseline hazard parametrised as a three-degrees-of-freedom cubic spline; predictions standardised to the covariate distribution of the cancer survivor group.

Table S30: Results from systematic review

Study (author, year)	Study Design	Data source/ setting	Cancer sites	Control group	Start follow-up	Exclusions	Age profile in years	Fracture outcomes	Adjustment covariates	Handling of prior fractures	Study size	Average follow-up	Main results
Rees-Punia, 2023	Cohort	Prospective study of cancer patients, linked with Medicare claims, 1999-2017, United States	Any	No cancer history at start follow-up, selected from Medicare	Latest of receipt of survey sent in 1999- and 1-year following cancer diagnosis	Non-invasive cancer, unverified cancer	Mean [SD] 69.4 [6.0]	Any fracture and site-specific fracture	Age, sex, race, ethnicity, region, physical activity, BMI, alcohol consumption, smoking, comorbidity score, diet quality, age at menopause, HRT, self-reported fracture history prior to baseline, radiotherapy, and chemotherapy	In adjustment set	No cancer history: 78 272 Any cancer: 14 159	Unclear	Cancer survivors diagnosed 1 to less than 5 years earlier with advanced stage cancer had higher risk of fracture (HR, 2.12; 95% CI, 1.75-2.58). The higher fracture risk in cancer survivors with recent advanced stage diagnosis (vs no cancer) was driven largely by vertebral (HR, 2.46; 95% CI, 1.93-3.13) and hip (HR, 2.46; 95% CI, 1.84-3.29) fracture sites. Evidence of increased risk fracture in 4 years following diagnosis, but not thereafter.
Hsieh, 2018	Cross-sectional	Cross-sectional study, China	Breast	Healthy community-dwelling postmenopausal women from Beijing, age- and BMI-matched	N/A - cross-sectional study	1) initiated breast cancer therapy in last 5 years, 2) history of bone metastases, 2) osteoporosis or osteoporosis therapy prior to breast cancer diagnosis, 3) metabolic or inherited bone disease, 4) corticosteroid or anticonvulsive therapy for > 6 months or within the last 12 months, 5) conditions leading to secondary osteoporosis.	50–70 years at enrolment	Vertebral	Age, BMI, Parity, Age of Menarche, 25OHD level, Education, Personal History of Fracture, Calcium Supplement Use	In adjustment set	Breast cancer: 200 Controls: 200	N/A - cross-sectional study	OR 4.16 (95%CI 1.69–10.21)

Study (author, year)	Study Design	Data source/ setting	Cancer sites	Control group	Start follow-up	Exclusions	Age profile in years	Fracture outcomes	Adjustment covariates	Handling of prior fractures	Study size	Average follow-up	Main results
Fraenkel, 2015	Cohort	Medical records among women undergoing bone mineral density (BMD) tests between 2003 and 2011 at a medical centre, Israel	Breast cancer at BMD test	Women without breast cancer at BMD test	BMD test	Participants under 18 years at BMD test	Age at first fracture: Breast cancer: 68.8 (±8.8) No breast cancer: 68.8 (±10.2)	Osteoporotic fracture	Age, BMI and BMD	Not reported	17110	Median 4.9 years	Any fracture: HR 1.34 (95% CI 1.04-1.73)
Kanis, 1999	Cohort	Data from breast cancer trials assessing effects of clodronate	Breast, incident, prevalent or recurrent	Community based controls, age-matched	Study enrolment	Skeletal metastases	Controls aged 45-69 years	Vertebral	Age, duration of follow-up and prevalent fracture	In adjustment set	Controls: 776	Mean Incident breast cancer: 2.1 Soft-tissue relapse: 1.8 Controls 2.9	Incident breast cancer: OR 4.7 (95% CI 2.3–9.9) Women with soft-tissue metastases without evidence of skeletal metastases: OR 22.7 (95% CI, 9.1–57.1)
Melton, 2012	Cohort	All women who resided in Olmsted County when first diagnosed with tissue-confirmed, invasive breast cancer in 1990to 1999, USA	Breast	Expected numbers were derived from local calendar year-, age- and sex-specific incidence rates	Breast cancer diagnosis	Non-invasive cancer	mean age61.6 (14.8) year	Any fracture and site-specific fracture	Calendar year, age and sex	Not reported	608 with breast cancer	median 10.4 year	Any site: standardized incidence ratio 1.8 (1.6–2.1)
Tsa, 2013	Cohort	Claims data 2000-2009, Taiwan	Breast, diagnosed between 2000-2003	Women without cancer, frequency matched with age and index date (the date for a health care visit).	Breast cancer diagnosis	Age under 20 years, diagnosis of other cancer, history of hip, distal forearm, and vertebral fracture.	≥ 20 years	Any fracture and site-specific fracture	Age, area, fracture history	Excluded women with history of major fracture at baseline and included other fractures in adjustment set	Breast cancer: 22,076 Controls: 88,304	Not specified	Any fracture: HR 1.16 (95% CI 1.07–1.27) Hip: HR 1.18 (95% CI 1.03-1.35) Vertebral: HR 1.24 (95% CI 1.04-1.48)

Study (author, year)	Study Design	Data source/ setting	Cancer sites	Control group	Start follow-up	Exclusions	Age profile in years	Fracture outcomes	Adjustment covariates	Handling of prior fractures	Study size	Average follow-up	Main results
Chen, 2005	Cohort	Prospective cohort study within the Women's Health Initiative, using questionnaire data from post-menopausal women enrolled 1994-1998, United States	Breast (history of at enrolment)	Cancer-free individuals at enrolment	Enrolment in cohort study	Women with history of cancer other than breast cancer at enrolment	50-79 at enrolment	Any fracture and site-specific fracture	Age, weight, ethnicity, and geographic region of enrolment	Further adjusted for fracture history in secondary analyses; results were unchanged.	Prevalent breast cancer: 5298 Controls: 80848	5.1	Any: HR 1.31 (95% CI, 1.21-1.41) Hip: HR 0.93 (95%CI 0.64-1.33) Forearm or wrist: HR 1.36 (95% CI, 1.16-1.59) Other: HR 1.31(95% CI, 1.19-1.43)
Pawloski, 2013	Cohort	Multi-site prospective cohort study, United States	Breast cancer, diagnosed 5 years previously	Cancer-free individuals, matched on age, study site, and enrolment year	5 years following breast cancer diagnosis	breast cancer recurrence in the 5 years preceding the study period	≥ 65 years	Any fracture and site-specific fracture	Study site, age, race, BMI, tumour, treatment variables at diagnosis, smoking status, comorbid conditions, drug exposure associated with an effect on bone health	Data on history of fracture not available	Breast cancer: 1286 Controls: 1286	Median, cancer survivors: 6.0 years; controls: 6.5 years	Any fracture: HR 1.1 (95% 0.9–1.3)
Lamont, 2003	Cohort	National Cancer Institute's Surveillance Epidemiology and End Results (SEER) Medicare program and a 5% sample of Medicare, post-menopausal women, United States	Breast cancer, diagnosed aged 55 and 64 years	Women without breast cancer	Age 67 years	Patients with stage III or IV disease or active breast cancer at ages 65 or 66 years	≥ 67 years	Hip	Age, race, socioeconomic status, geographic location, cohort entry year, and medical comorbidity.	Not reported	Breast cancer: 5980 Controls: 23165	Maximum 5 years	RR 0.63 (95% CI: 0.43–0.94)

Study (author, year)	Study Design	Data source/ setting	Cancer sites	Control group	Start follow-up	Exclusions	Age profile in years	Fracture outcomes	Adjustment covariates	Handling of prior fractures	Study size	Average follow-up	Main results
Chen, 2009	Cohort and trial data	Women's Health Initiative, post-menopausal women enrolled 1994-1998 in clinical trial or observational study, United States	Breast or other cancer	Cancer-free individuals at enrolment	Enrolment in cohort study or trial	Women with history of cancer at enrolment	50-79 years at enrolment	Any fracture and site-specific fracture	Age, years since menopause, ethnicity, education, living with partner, smoking, general health, parity, hormone therapy, sedatives or psychotics or hypnotic drug use, depressive symptoms or anti-depressant use, arthritis, corticosteroid use, weight, height, physical activity, total calcium intake, vitamin D intake, energy intake, alcohol, caffeine, parent broke bone after age 40, family history of cancer, Gail score, HT trial assignment, DM trial assignment, CaD trial assignment, study participation, hysterectomy status and history of fracture after 55 years of age.	History of fracture after 55 years of age in adjustment set	Incident invasive BC: 4,804<br>Incident other cancer: N=8,242<br>No-cancer: 132,840	7.8 (1.7)	Any fracture<br>Breast: HR 1.02 (0.89, 1.18)<br>Other cancer: HR 1.33 (1.18, 1.49)<br><br>Hip<br>Breast: HR 1.55 (1.13, 2.11)<br>Other cancer: HR 2.09 (1.65, 2.65)
Choi, 2021	Cohort	Population-based survey (baseline and follow-up survey) among female urban resident participants 40-79 years at baseline, 2006-2016, Korea	Cervical (obtained at baseline)	Women without cervical cancer	2012	Participants with history of cancer; missing data on height, weight, smoking, alcohol use, hypertension, diabetes, dyslipidaemia, arthritis, thyroid disease, osteoporosis, oophorectomy.	Cancer cases: 55.0 (8.1)<br>Controls: 52.6 (8.1)	Osteoporotic fracture	BMI, the number of children, the summation of oral contraceptive medication year, hypertension, diabetes mellitus, dyslipidaemia, arthritis, thyroid disease, smoking, drinking alcohol, history of hysterectomy, history of oophorectomy, and osteoporosis	Prior fractures may be included in the outcome	cervical cancer (n=493) and control participants (n= 77,571)		OR 1.06 (95% CI 0.82–1.38)

Study (author, year)	Study Design	Data source/ setting	Cancer sites	Control group	Start follow-up	Exclusions	Age profile in years	Fracture outcomes	Adjustment covariates	Handling of prior fractures	Study size	Average follow-up	Main results
Shin, 2019	Cohort	Korean national health insurance data	Gastric cancer after gastrectomy	General population, propensity score matched	Date of gastrectomy	Other cancers, treated with medications for osteoporosis, previous admission for osteoporotic fracture	mean age at gastrectomy was 58.4 (611.8).	Osteoporotic fracture	Propensity score matched on baseline age, sex, residence, income, and disability.	Excluded individuals with history of osteoporotic fractures	133179 cancer survivors 133179 controls	mean 4.50 years	Any gastric cancer: HR 1.61 (95% CI 1.53–1.70)<br>Total gastrectomy: HR 2.18 (95% CI 1.96–2.44)<br>Subtotal gastrectomy: HR 1.48 (95% CI 1.40–1.57)<br>Adjuvant chemotherapy: HR 2.01 (95% CI 1.81–2.23)<br>(all compared to cancer-free comparators)
Melton, 2005	Cohort	All women who resided in Olmsted County when first diagnosed with myeloma in 1945 to 2001, US	Multiple myeloma	Expected numbers of fractures were derived from local calendar year-, age- and sex-specific incidence rates	Myeloma diagnosis	None reported	mean age, 70.7 ± 11.1 years	Any fracture and site-specific fracture	Calendar year, age and sex	Not accounted for in analysis	165 with myeloma	Total follow-up reported: 537 person-years (mean 3.3 years)	Any fracture: SIR 10.88.9 (7.2–11)
Oortgiesen, 2020	Case-control	Administrative data, 1996-2011, split into three cohorts according to date of fracture was: 1996–2000, 2001–2006 and 2007–2011, Denmark	Multiple myeloma	Individuals without a fracture	N/A - case-control study	None	Mean age in each time cohort was 53 (SD 21.9), 54 (SD 21.1), and 55 (SD 20.6) years,	Any fracture and site-specific fracture	Use of antidepressants, bisphosphonates, antiepileptics, hypnotics, proton pump inhibitors in the past 6 months, malignancies, and fractures prior to 1996.	In adjustment set	925,341 cases 925,341 controls	N/A - case-control study	Any fracture:<br>1996–2000: OR 1.7 (95% CI 1.3–2.3)<br>2001–2006: OR 1.3 (95% CI 1.1–1.6)<br>2007–2011: OR 1.7 (95% CI 1.4–2.2)

Study (author, year)	Study Design	Data source/ setting	Cancer sites	Control group	Start follow-up	Exclusions	Age profile in years	Fracture outcomes	Adjustment covariates	Handling of prior fractures	Study size	Average follow-up	Main results
Nelson, 2023	Cohort and trial data	Women's Health Initiative, post-menopausal women enrolled 1994-1998 in clinical trial or observational study, United States	Pelvic	Women without pelvic cancer	Enrolment in cohort study or trial	Women with history of cancer at enrolment	50-79 years at enrolment	Pelvic	Race, ethnicity, education, physical activity, bisphosphonate use, FRAX 10-year hip fracture risk, history of treated diabetes, hysterectomy, bilateral oophorectomy, calcium intake, and vitamin D intake	Adjusted for 10-year hip fracture risk, which includes fracture history	Pelvic cancer: 4451 No pelvic cancer: 131,292	17.7 years from enrolment	Pelvic fracture: HR=1.26 (95% CI 1.11 to 1.43) Risk only observed in those undergoing pelvic radiation. No increased risk of radial fracture
Abrahamsen, 2007	Case-control	Registry data 1977-2000, Denmark	Prostate	Men without fracture, age matched.	N/A - case-control study	None reported	>50 years	Any fracture and site-specific fracture	Age, prior fracture, single living and income band	In adjustment set	Fracture cases: 15716 Controls: 47149	N/A - case-control study	All fractures: OR 2.2 (95% CI 1.9–2.5) Hip: OR 2.4 (95% CI 2.0–2.9) Vertebral: OR 1.1 (95% CI 0.7–1.6) Risk was similar according to time since diagnosis.
Shin, 2020	Cohort	National health records, Korea	Prostate	General population controls	Follow-up started 1-year post-cancer	age < 40 years, history of disability registration, osteoporotic fracture, and follow-up < 1 year	≥ 40 years	Any fracture and site-specific fracture	Age, income, diabetes mellitus, hypertension, dyslipidaemia, chronic kidney disease, osteoporosis, rheumatoid arthritis, and Charlson comorbidity index	Excluded those with history of osteoporotic fracture	Prostate cancer: 41,733 Controls	4.3 and 4.6 years for the prostate cancer survivors and matched control subjects, respectiv ely	Any fracture: HR 1.39 (95% CI 1.33–1.45) Among those with ADT treatment: HR 1.92 (95% CI 1.82, 2.02) Among those with surgery: HR 0.89 (95% CI 0.82, 0.96) Similar patterns seen for vertebral, hip and other fractures
Papaleontiou, 2019	Cohort	Veterans Health Database 2004-2013, United States	Thyroid	Individuals without thyroid cancer, matched on age, sex, weight, steroid use	Unclear	Patients with traumatic or pathological fractures were excluded from the analyses. Thyroid cancer patients not on HRT or without at least 2 outpatient TSH measurements after cancer diagnosis.	≥ 18 years	Any fracture	median household income, Charlson/Deyo comorbidity score, and androgen use	Not accounted for.	thyroid cancer: 10,370 controls: 10,370	52 months	Any fracture: OR 0.72 (95% CI 0.60–0.86)

Study (author, year)	Study Design	Data source/ setting	Cancer sites	Control group	Start follow-up	Exclusions	Age profile in years	Fracture outcomes	Adjustment covariates	Handling of prior fractures	Study size	Average follow-up	Main results
Vesterguaard, 2009	Case-control	Registry data for all inhabitants 1977 to 2000, Denmark,	Various	Age and gender matched controls	N/A - case-control study	None reported	Any age	Any fracture and site-specific fracture	Other cancers, use of corticosteroids, alcoholism, use of pain medication (morphine and other opioid agonists, NSAIDs, Acetylsalicylic acid, and acetaminophen), and prior fracture	In adjustment set	Fracture cases: 124 655 Controls: 373 962	N/A - case-control study	Lung cancer, prostate cancer, multiple myeloma, bone metastases, and metastases in other locations were associated with an increased overall risk of fractures. The other cancer types were not associated with risk of any fracture.<br><br>Some evidence of high risk of fracture early after diagnosis, which then declined.

Database and interface: Ovid MEDLINE(R) ALL 1946 to May 11, 2023; **Date of search:** 15-05-2023.

Search expression:

1.	exp Neoplasms/
2.	(cancer* or neoplas* or malignanc*).mp. [mp=title, book title, abstract, original title, name of substance word, subject heading word, floating sub-heading word, keyword heading word, organism supplementary concept word, protocol supplementary concept word, rare disease supplementary concept word, unique identifier, synonyms, population supplementary concept word, anatomy supplementary concept word]
3.	1 or 2
4.	(survivor* or recover*).mp. [mp=title, book title, abstract, original title, name of substance word, subject heading word, floating sub-heading word, keyword heading word, organism supplementary concept word, protocol supplementary concept word, rare disease supplementary concept word, unique identifier, synonyms, population supplementary concept word, anatomy supplementary concept word]
5.	3 and 4
6.	cancer survivor.mp. or exp Cancer Survivors/
7.	5 or 6
8.	bone fracture.mp. or exp Fractures, Bone/
9.	7 and 8

Results: 427

Figure S1A: Flowchart of the selection of the cohorts used in analysis: CPRD GOLD primary care dataset.

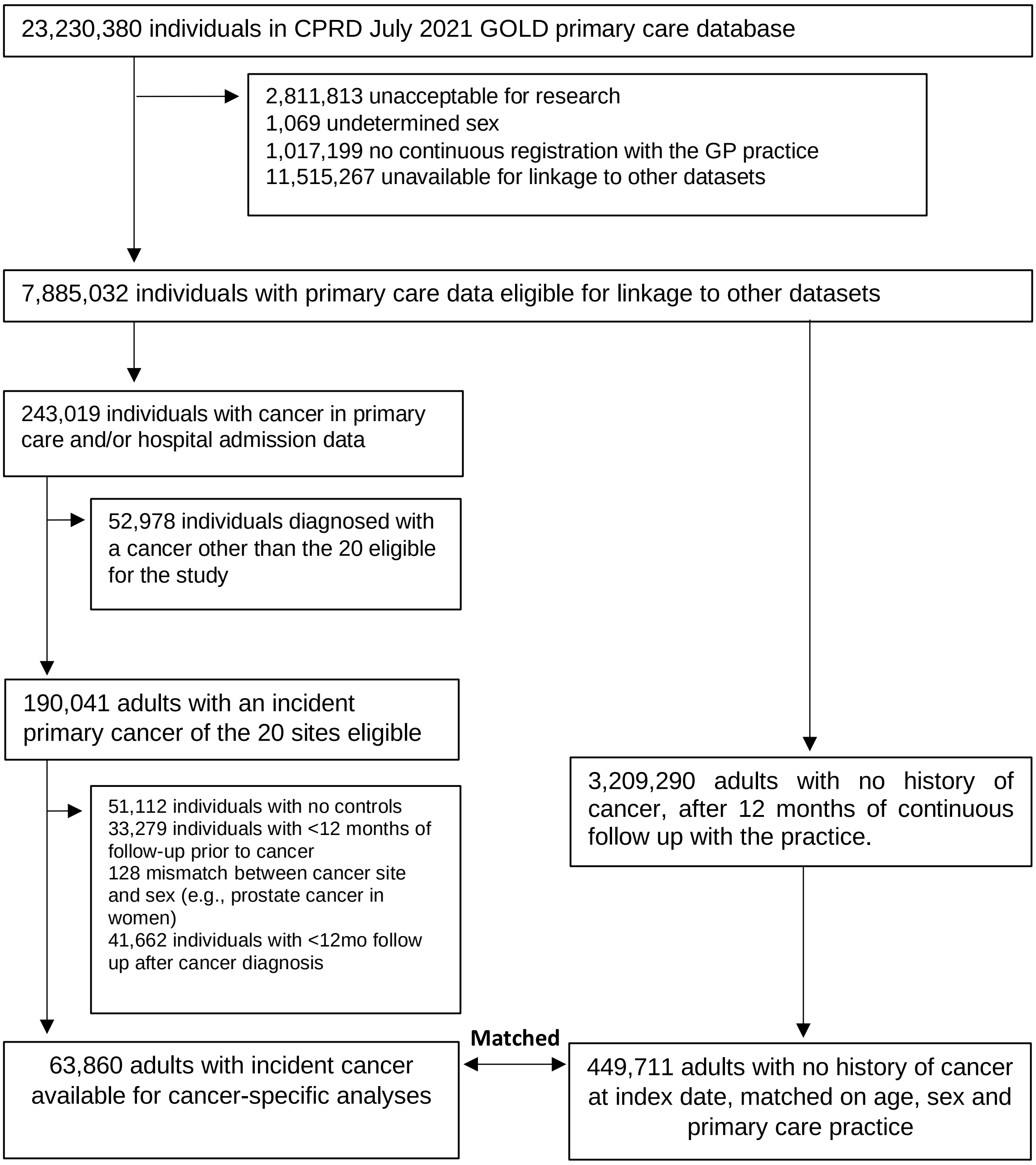

Figure S1B: Flowchart of the selection of the cohorts used in analysis: CPRD Aurum primary care dataset.

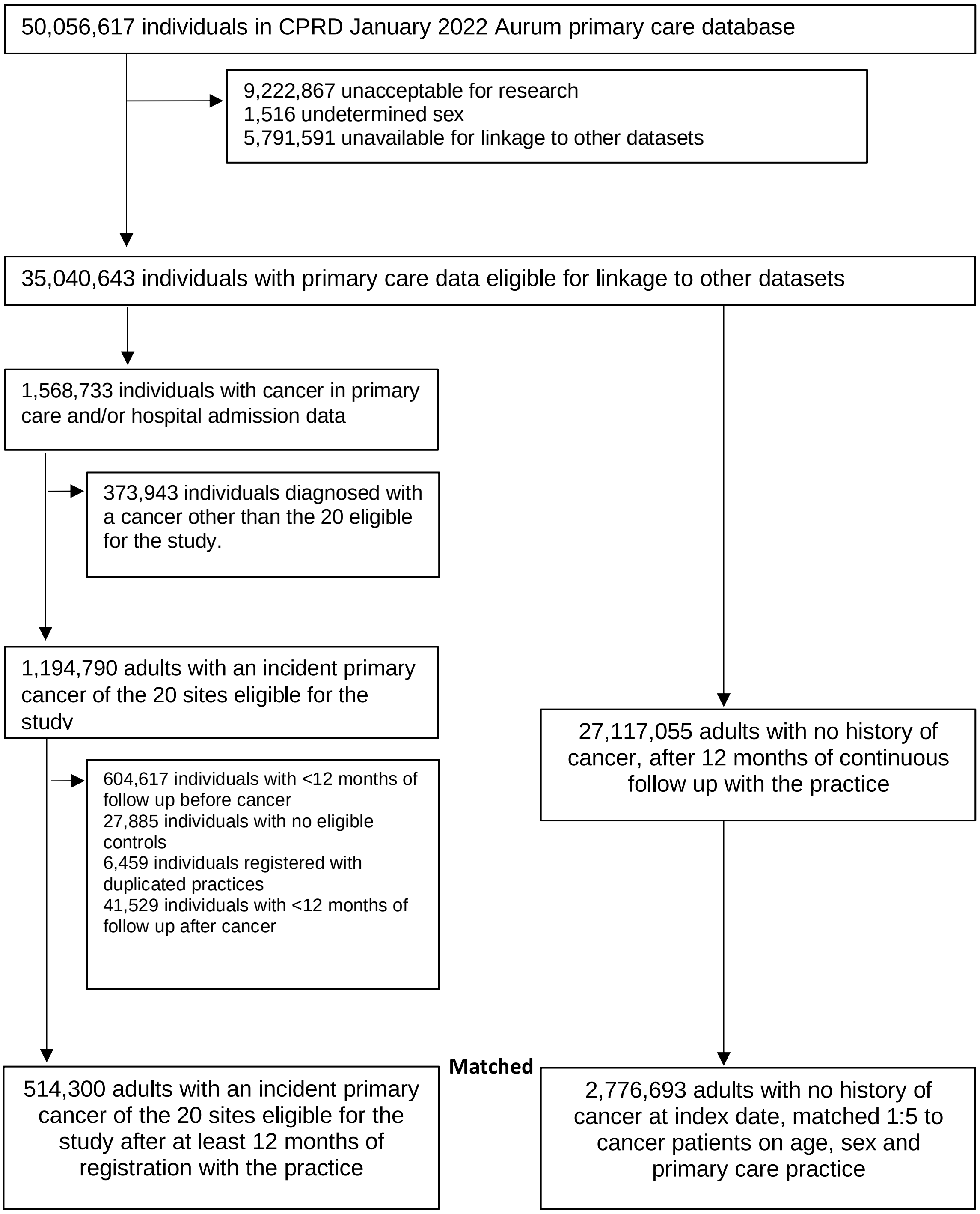

NB. In the main analysis, follow up started 1-year post-index date (i.e. the date of cancer diagnosis in the exposed cohort; the date of cancer diagnosis of the matched pair in the unexposed cohort). In sensitivity analysis, we started follow up at index date.

Figure S2: Associations between cancer survivorship and fractures of the pelvis, hip, spine, wrist and proximal humerus.

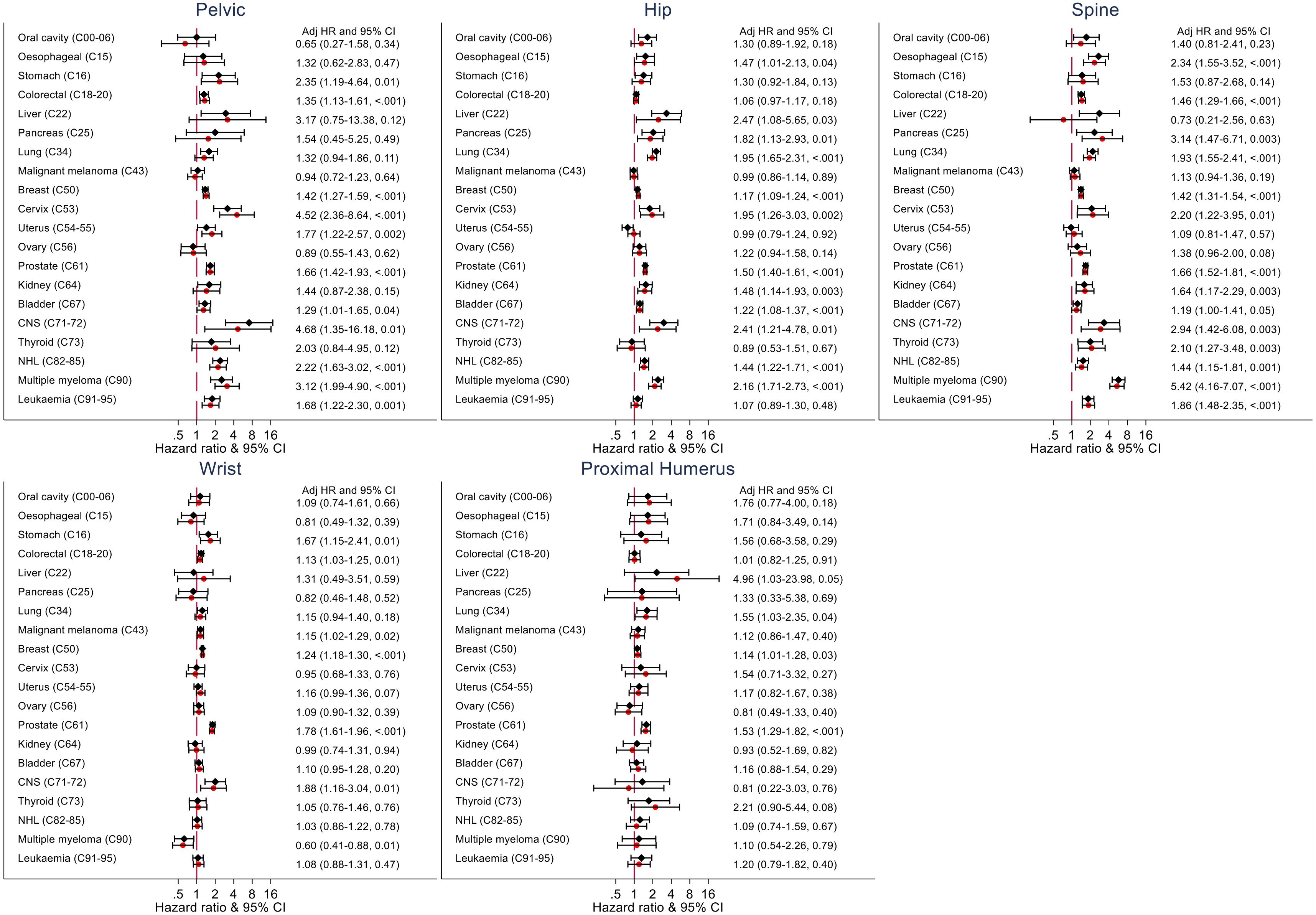

Black diamonds = Results from minimally adjusted models; Red circles = Results from fully adjusted models. Right-hand column within each panel shows HRs, 95% CIs, p-values. Cox proportional hazards regression model was used to estimate the hazard ratios. Minimally adjusted estimates were controlled for the matching factors only (age, sex and general practice). Fully adjusted models also included problem drinking, smoking status, body mass index, patient-level postcode-based Index of Multiple Deprivation, chronic kidney disease, autoimmune disorders, liver disease, epilepsy, and use of corticosteroids, hormone replacement therapy, and bisphosphonates.

Figure S3: Cumulative incidence of major fractures during the study period in cancer survivors and non-cancer controls by sex, with 95% confidence intervals.

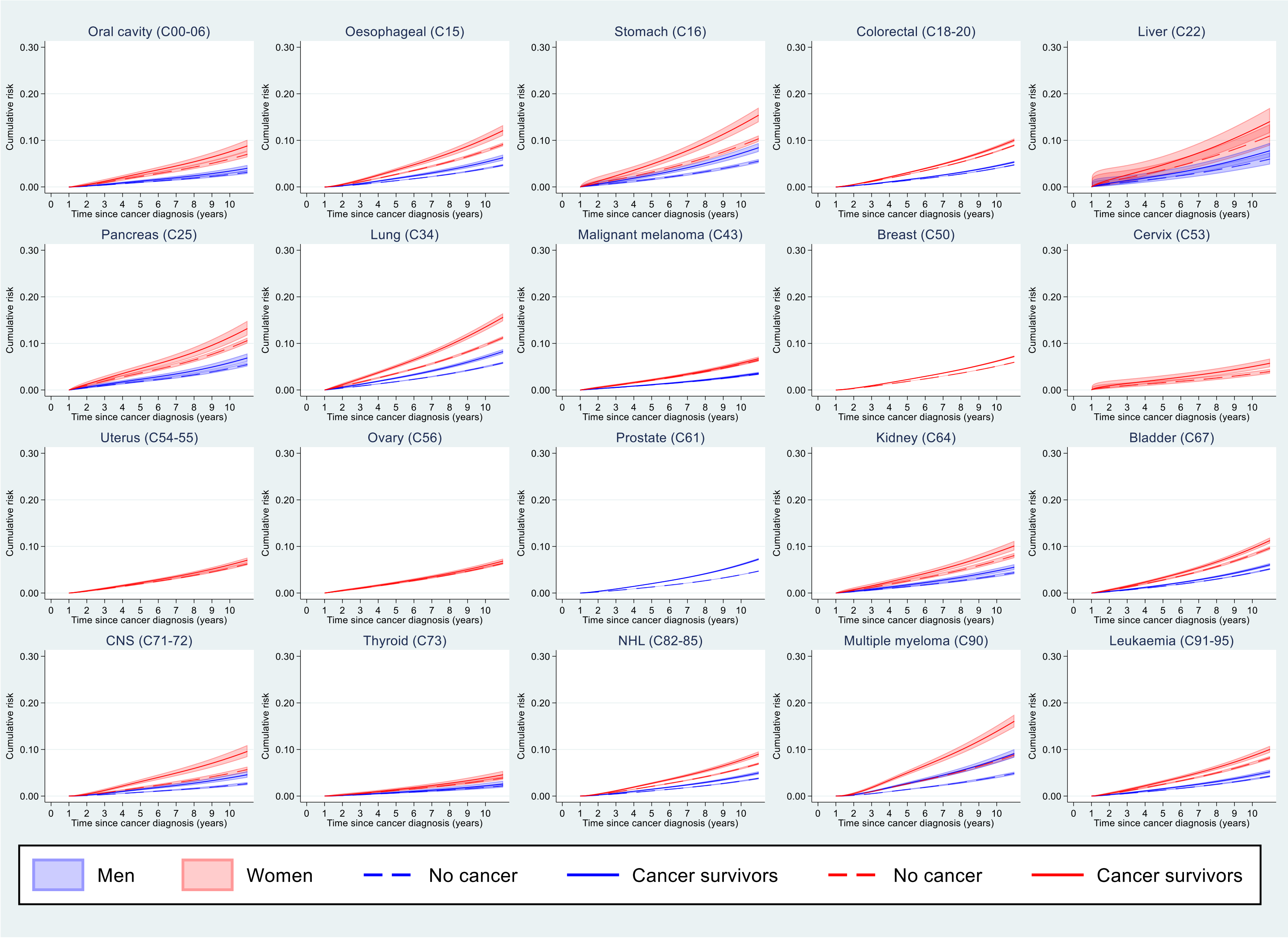

Cumulative incidence predicted from a Royston-Parmar model including age, sex (when applicable), BMI (cubic spline), index of multiple deprivation, smoking, problematic alcohol use, use of corticosteroids, bisphosphonates, and hormone replacement therapy, and history of chronic kidney disease, liver disease, epilepsy, and autoimmune conditions, with the baseline hazard parametrised as a three-degrees-of-freedom cubic spline; predictions standardised to the covariate distribution of the cancer survivor group.

Figure S4: Forest plots of the association between cancer survivorship and any fracture, stratified by potential effect modifiers.

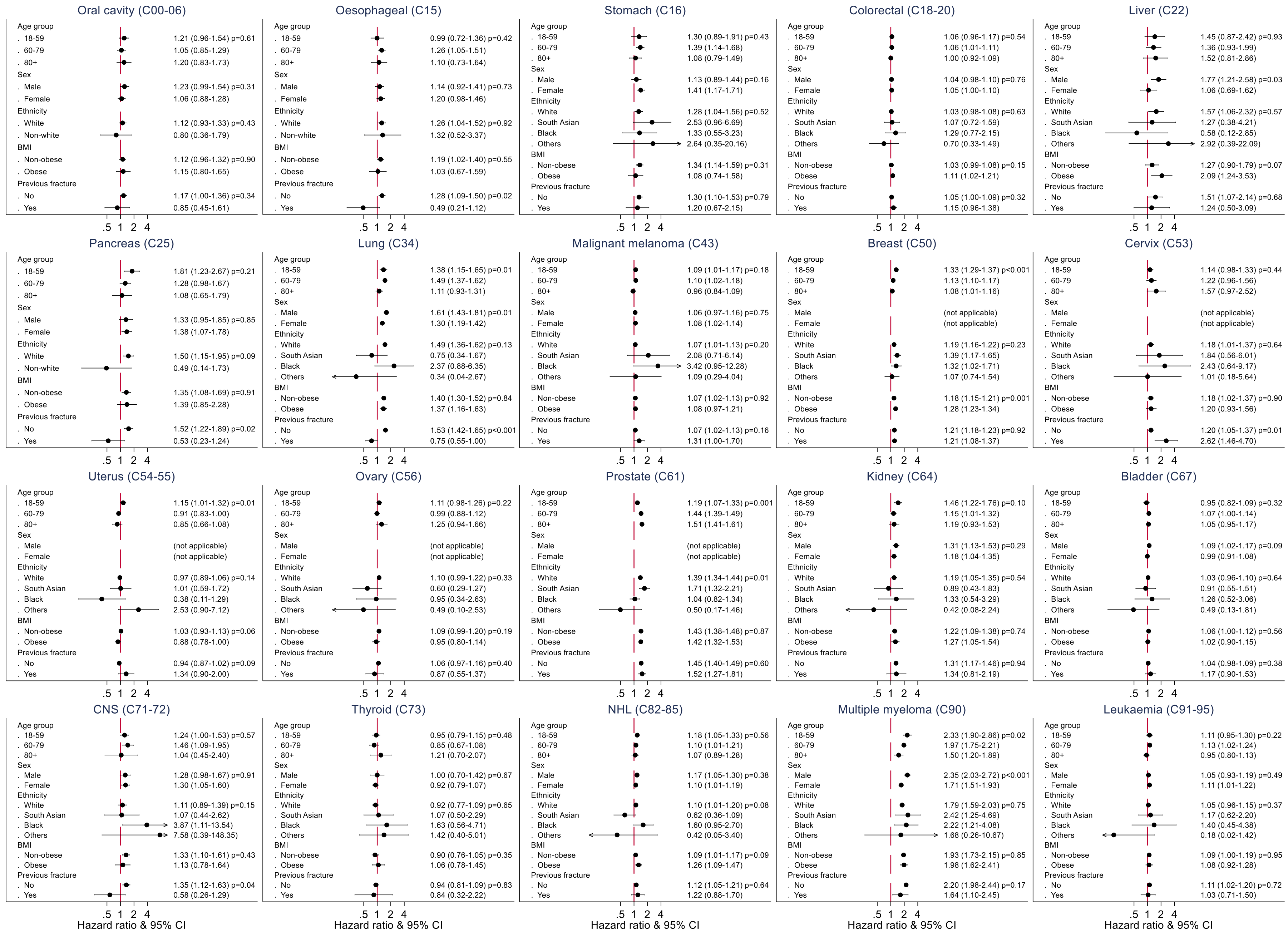

Figure S5: Forest plots of the association between cancer survivorship and major osteoporotic fractures, stratified by potential effect modifiers.

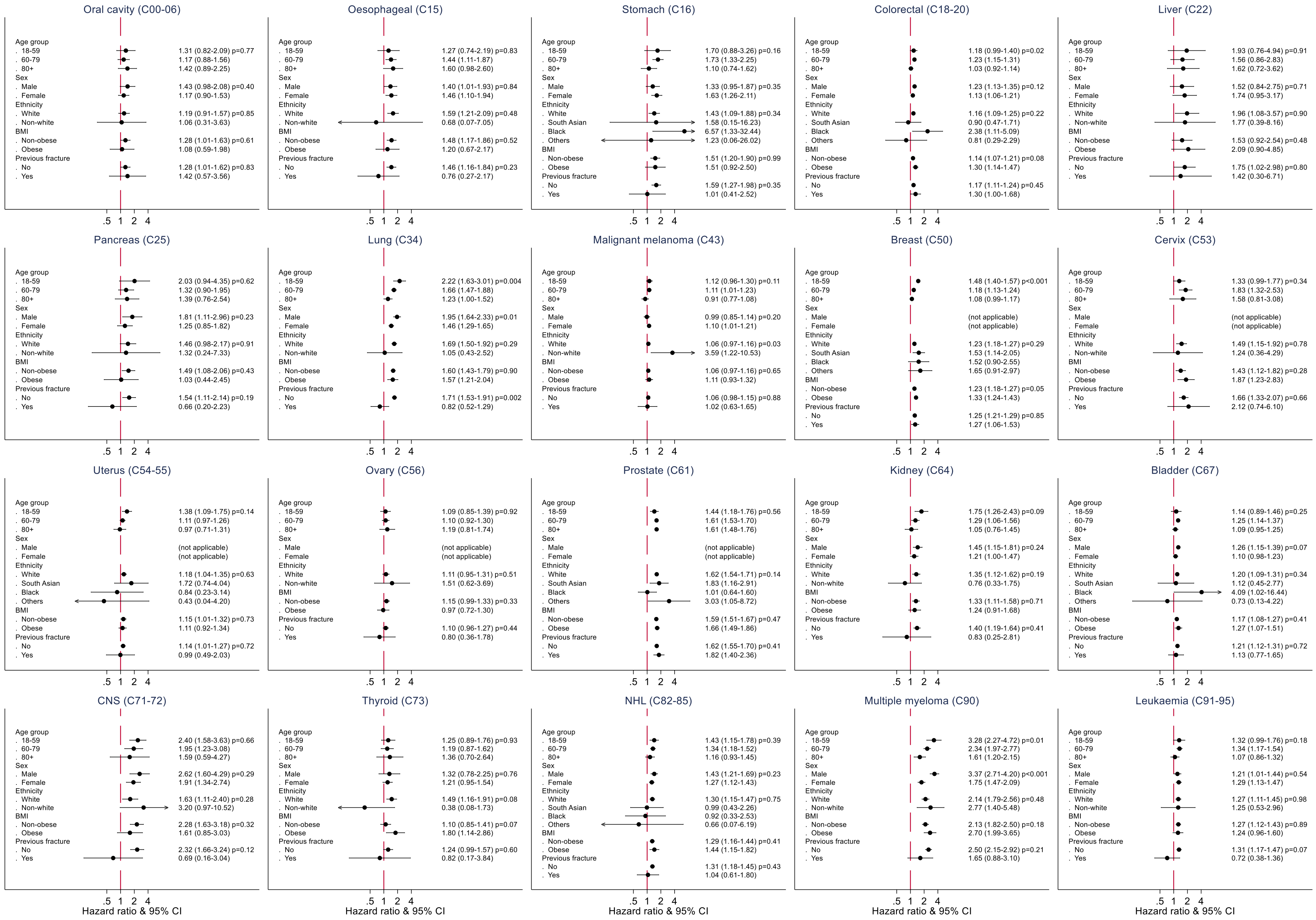

Figure S6: Results of sensitivity analysis on the association between cancer survivorship and any fracture.

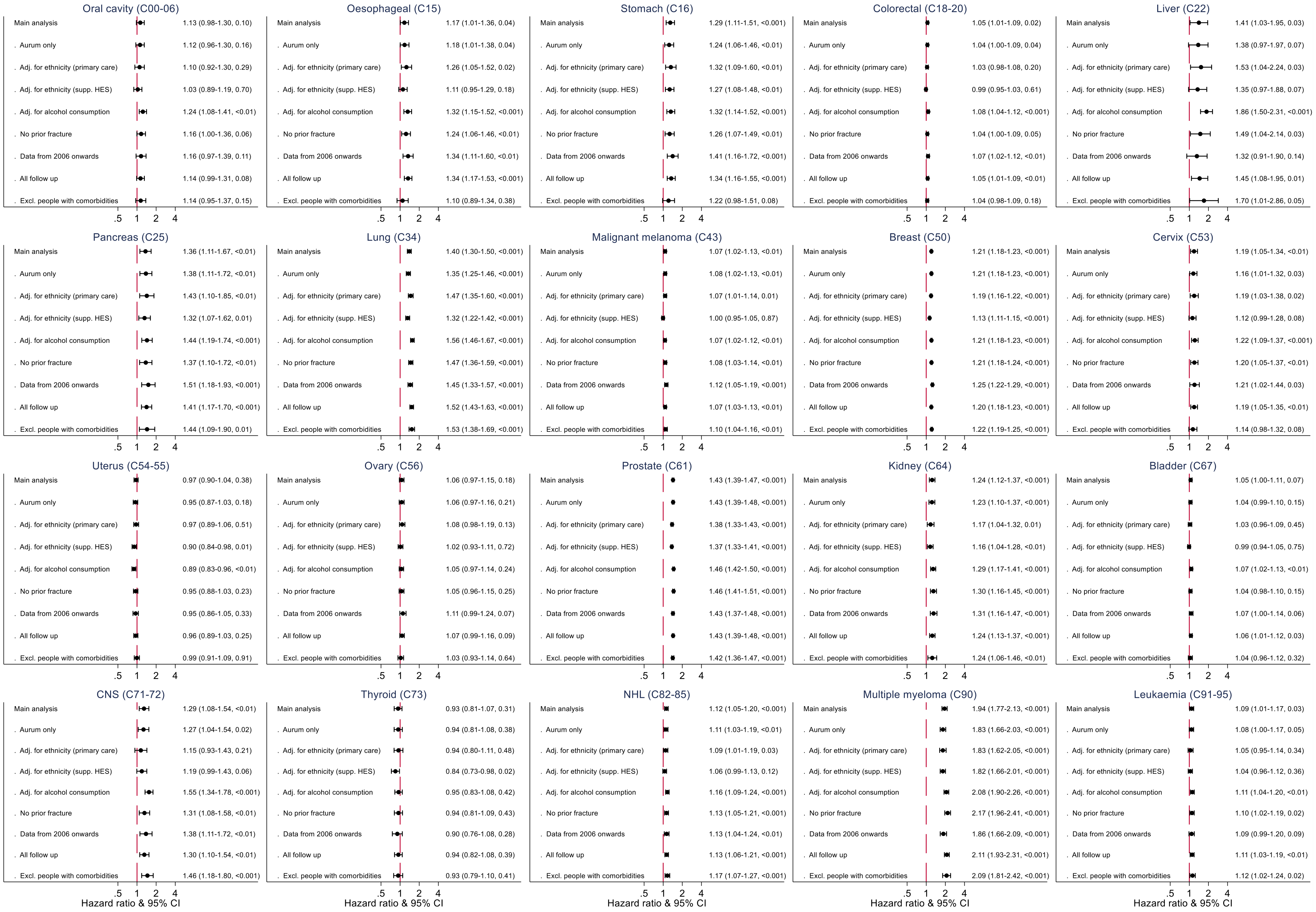

Figure S7: Results of sensitivity analysis on the association between cancer survivorship and major osteoporotic fractures.

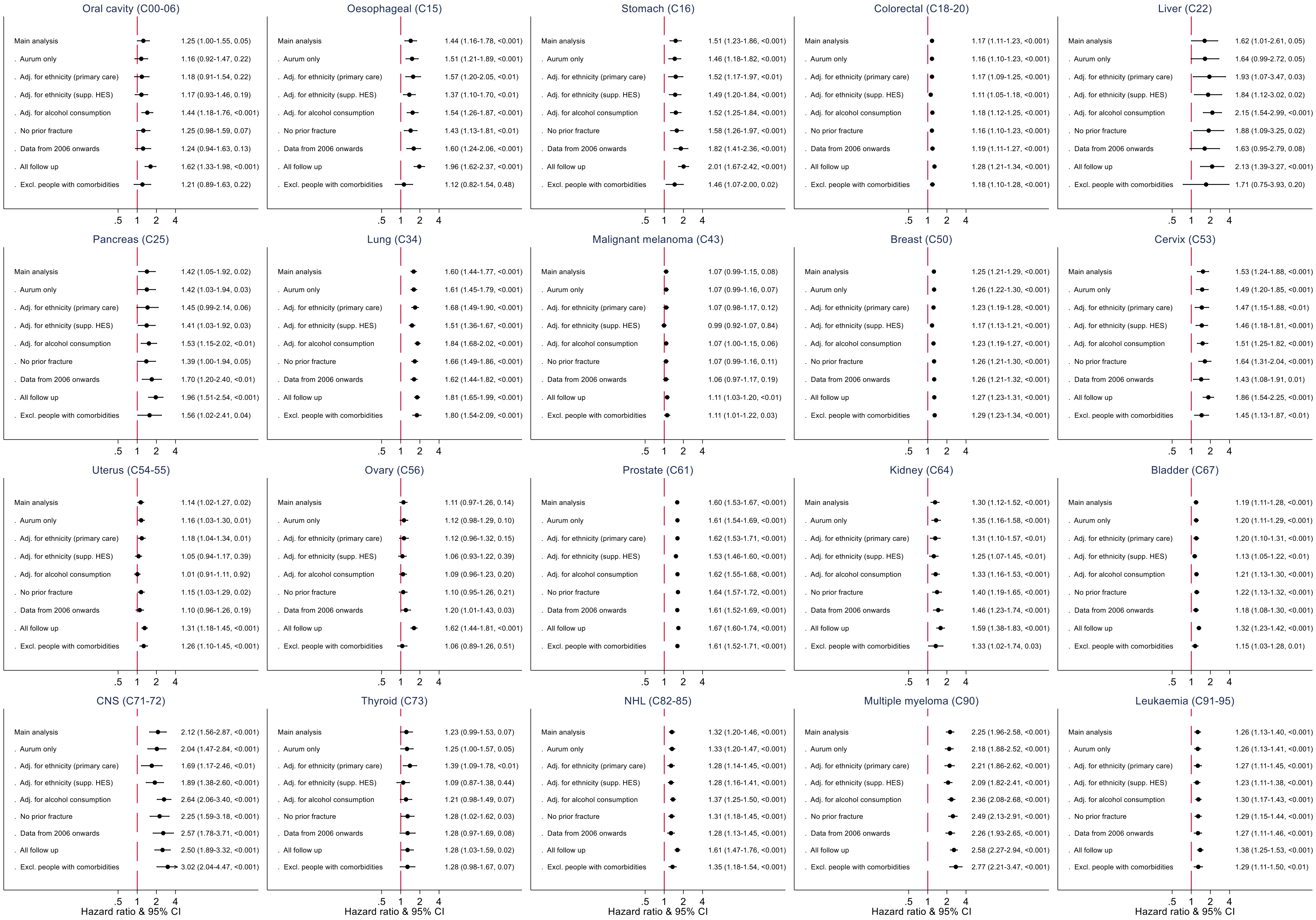

Supplement: Supplementary appendix [file mmc1.pdf]
